# Planet-compatible pathways for transitioning the chemical industry

**DOI:** 10.1073/pnas.2218294120

**Published:** 2023-02-14

**Authors:** Fanran Meng, Andreas Wagner, Alexandre B. Kremer, Daisuke Kanazawa, Jane J. Leung, Peter Goult, Min Guan, Sophie Herrmann, Eveline Speelman, Pim Sauter, Shajeeshan Lingeswaran, Martin M. Stuchtey, Katja Hansen, Eric Masanet, André C. Serrenho, Naoko Ishii, Yasunori Kikuchi, Jonathan M. Cullen

**Affiliations:** ^a^Department of Engineering, University of Cambridge, Cambridge CB2 1PZ, UK; ^b^Systemiq, London EC4V 5EQ, UK; ^c^Center for Global Commons, Institute for Future Initiatives, The University of Tokyo, Bunkyo-ku, Tokyo 113-8654, Japan; ^d^Faculty of Business and Management, Innsbruck University, Innsbruck 6020, Austria; ^e^Institute of Energy Efficient and Sustainable Design and Building, Technical University ounich 80333, München, Germany; ^f^Bren School of Environmental Science and Management, University of California, Santa Barbara, CA 93117; ^g^Department of Mechanical Engineering, University of California, Santa Barbara, CA 93117; ^h^Department of Chemical System Engineering, The University of Tokyo, Bunkyo-ku, Tokyo 113-8656, Japan; ^i^Institute for Future Initiatives, The University of Tokyo, Bunkyo-ku, Tokyo 113-8654, Japan

**Keywords:** plastics, fertilizers, chemicals, climate change, circularity

## Abstract

Chemical products, such as plastics, solvents, and fertilizers, are essential for supporting modern lifestyles. Yet, producing, using, and disposing of chemicals creates adverse environmental impacts which threaten the industry’s license to operate. This study presents seven planet-compatible pathways toward 2050 employing demand-side and supply-side interventions with cumulative total investment costs of US$1.2–3.7 trillion. Resource efficiency and circularity interventions reduce global chemicals demand by 23 to 33% and are critical for mitigating risks associated with using fossil feedstocks and carbon capture and sequestration, and constraints on available biogenic and recyclate feedstocks. Replacing fossil feedstocks with biogenic/air-capture sources, shifting carbon destinations from the atmosphere to ground, and electrifying/decarbonizing energy supply for production technologies could enable net negative emissions of 0.5 GtCO_2eq_ y^−1^ across non-ammonia chemicals, while still delivering essential chemical-based services to society.

## Significance

The chemical industry underpins modern society across manufactured goods, food production and energy security via the production of plastics, solvents, fertilizers and more. However, it simultaneously presents multiple threats to the planetary boundaries that will undermine the industry's license to operate, requiring major and rapid system transformation. Our study presents seven planet-compatible pathways for transitioning the industry towards net-zero, employing both demand- and supply-side interventions to chemicals representing over 70% of the sector’s emissions. The pathways rely on circular strategies and suggest that the chemical industry has the option to become a carbon steward ultimately providing much-needed negative emissions to society. Imminent action and implementation are required globally to enable this radical transformation and unlock the presented pathways.

The modern chemical industry stands at a crossroads. Following decades of fast-paced growth, chemicals and their derivatives are now ubiquitous in society (1, 2) and essential for supporting modern lifestyles, contributing over 7% of the global domestic product in 2017 (3). Moreover, in the future, chemicals will likely play a significant role in delivering net-zero targets (e.g., ammonia for shipping).

While we use thousands of chemicals in our lives (4), most of them are derived from eight primary chemicals, namely ammonia, methanol, ethylene, propylene, benzene, toluene, and mixed xylenes. Ammonia is the base chemical for all nitrogen fertilizers, which are critical for improving agricultural yields in food production (5). Methanol—the simplest alcohol—is a chemical building block for adhesives, paints, and construction materials (6). Approximately 60% of methanol is used as precursor chemicals in production, such as acetic acid (or vinegar) and formaldehyde, used in the production of particle boards and coatings (7). Ethylene, propylene, and butadiene (the most important olefins) are used as raw materials in the production of chemical and polymer products such as plastics, detergents, adhesives, and rubber. Benzene, toluene, and xylene (known as aromatics) are key building block chemicals for consumer products like aspirin, refrigerants, and textiles (8). About 45% of benzene is used in the production of polystyrene plastics, used in foam insulations and single-use cups, while 82% of xylenes are used to produce polyethene terephthalate plastics, used in plastic bottles (9).

However, the industry faces multiple planet-wide environmental dilemmas. Energy-intensive chemical production processes consumed 14% of global oil and 9% of global gas and released 13% of global industrial direct CO_2_ emissions in 2020 (10, 11). More greenhouse gases (GHGs) are released upstream and downstream of production, including fugitive methane from upstream extraction processes (12–15), CO_2_ from end-of-life plastics incineration (16), nitrous oxide (N_2_O) from fertilizer application (17), and indirect GHGs from the generation of electricity and heat (18). Predominantly linear value chains, agricultural inputs, and expanding land-use generate further environmental impact. For example, approximately 57% of the nitrogen in fertilizers is not absorbed by plants but instead finds its way into waterways and oceans (19–21), where it is joined by an estimated 11 Mt y^−1^ of plastics leaked from ineffective waste management systems (21–24).

At the heart of these issues is the uncontrolled leakage of chemicals to the environment via GHG emissions to the air and products to land, waterways, and marine environments (21, 25–28). This leakage has adverse effects on climate, biodiversity, ecosystems, and human health. Safeguarding planetary boundaries requires a transition toward more sustainable production and consumption of chemicals, where the leakage is eliminated or made harmless to the environment and emissions are reduced across all lifecycle stages (29).

Yet, the road ahead for chemicals is divided. The industry’s preferred pathway to net-zero is to retrofit current chemical facilities with supply-based emissions mitigation solutions while leaving demand for chemicals unconstrained. Supply-side technology options include carbon capture and storage (CCS) and utilization (CCU), bio-based/green hydrogen-based feedstocks, direct air capture (DAC), and electrification (30). Given these technologies are yet to be deployed at a meaningful scale (31), there is a significant risk of missing targets and locking in polluting systems by relying exclusively on this pathway. Opposing pathways, for instance, call for bans on plastics (32) or a move to 100% material circularity (33). These routes are equally risky, as there is often no better alternative to plastics, making bans impractical (1), and full material recovery is limited by thermodynamics laws and thus unattainable (34) (e.g., for fertilizer dissipation in the environment).

Given the risks in pursuing these pathways, new options must be found between the extremes that are feasible and able to mitigate planetary boundary risks to acceptable levels. This study aims to overcome the limitations of previously developed pathways (1, 31, 33, 35), by exploring net-zero emissions pathways that employ both demand-side and supply-side mitigation strategies, while applying constraints on technology (e.g., CCS) and feedstock availability (e.g., bio-based and recyclates) based on planetary boundaries. The result is lower-risk, technologically and financially feasible pathways which extend the solution frontier and place the chemical industry on a trajectory to meet 1.5 °C, 2050 targets, while respecting planetary boundaries.

## Results

### Future Projected Demand for Chemicals.

**Fig. 1.**
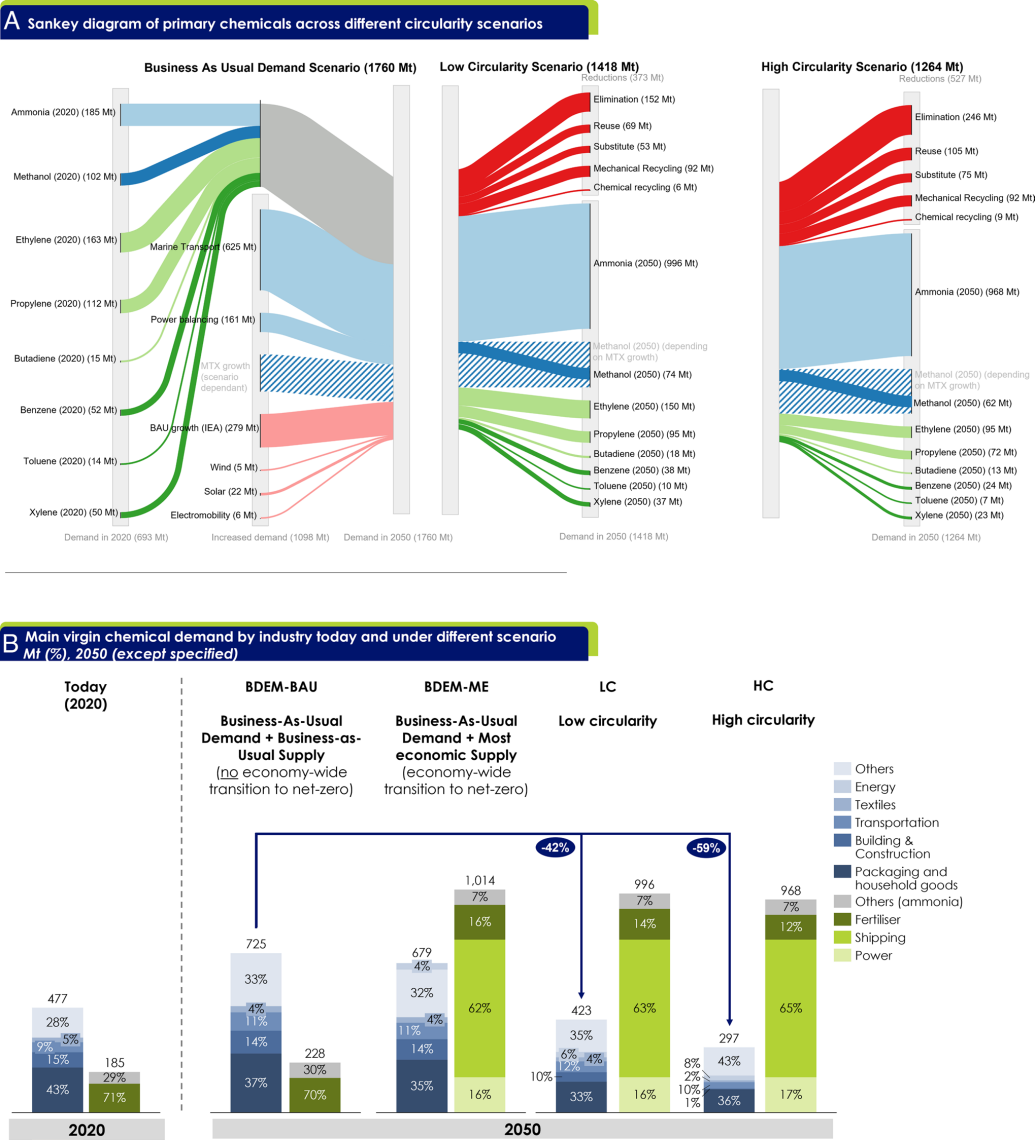
(*A*) Material demand changes (in Mt) of eight primary chemicals (ammonium nitrate and urea shown as part of ammonia) between 2020 and 2050 under LC, HC and Business as Usual Demand (BDEM) scenarios, respectively. MTX growth is dependent on supply scenario and illustrated for the low circularity demand scenario coupled with the most economic (ME) supply scenario (further details in text below). Flow width indicates the masses of chemicals; flow color is used to distinguish different types of chemicals; grey indicates the Business-as-usual (BAU) demand in 2020; red shows demand reduction through resource efficiency and circularity strategies, with improvements in agricultural practices included in the elimination wedge. Yearly volumes of each chemical over time including their supply technology mix can be found in *SI Appendix*, Figs. S27–S96. Chemical recycling in this study represents depolymerization and degradation (*SI Appendix*, section 4.10). (*B*) Key industries for virgin chemical demand under different scenarios. The major difference between BDEM-BAU and BDEM-ME is that under the former, it is assumed that the economy does not transition to net-zero. Hence, it is assumed in BDEM-BAU that there is no transition of the shipping industry from heavy fuel oil to ammonia, no rollout of renewable energy production, electric vehicles, or any improvement in mechanical recycling rates. The figure includes ammonia (& derivatives ammonium nitrate, urea) ethylene, propylene, methanol (but excludes MTX), benzene, toluene, xylene, and butadiene.

Projected demand for the analyzed chemicals increases rapidly between 2020 and 2050, assuming no toxicity regulatory changes for chemical uses (*SI Appendix*, section 4). This is primarily driven by new demand for chemical products, which is partially offset by resource efficiency and circularity strategies (Fig. 1). Demand (excluding methanol demand for methanol-to-olefins/propylene/aromatics (MTX)) rises from 693 Mt in 2020 to 1692 Mt in 2050 in the business-as-usual (BDEM) scenario but is reduced to 1418 Mt (19% reduction) in the low circularity (LC) scenario and to 1264 Mt (34% reduction) in the high circularity (HC) scenario.

Today’s ammonia production of 185 Mt represents 27% of the total chemicals produced, with ~70% used for nitrogen fertilizers. Ammonia for fertilizer use increases from 132 Mt (2020) to 158 Mt (2050) in the business-as-usual (BAU) scenario, while improved agricultural practices, diet shift, and food waste reduction lower demand to 140 Mt (2050, LC) and 112 Mt (2050, HC) respectively. While precise ammonia demand forecasts remain uncertain, a significant increase due to new demand is expected as ammonia is a promising pathway for decarbonizing long-distance marine transport and power in countries with highly limited renewable resources (5, 21, 22, 24). New demand means ammonia production accounts for ~900 Mt of the total primary chemicals (~ 60%, BDEM) in 2050.

Today, methanol provides an alternative feedstock for diversified end-products: 28% for polymers [i.e., polyethylene and polypropylene via methanol-to-olefin (MTO) route], 22% for predominate resins (via formaldehyde derivatives), 27% for fuel, and the remainder for other chemical products. In the presented pathways, methanol emerges as a new carbon platform chemical, with 200 to 900 Mt being converted to olefins via methanol-to-olefins/propylene (MTO/P) and to aromatics via methanol-to-aromatics (MTA). Hence, total demand including MTX varies from 2587 Mt (BDEM-ME) to 1480 Mt (HC-ME).

Three major olefins, accounting for 290 Mt in 2020 [ethylene (56%), propylene (39%), and butadiene (5%)], are used in a wide range of products, dominated by polymers (~84%). Resource efficiency and circular strategies are leveraged by four downstream industries—transportation, construction, packaging, and textiles—reducing overall demand for olefins by 26 to 42% between 2020 and 2050. However, demand for ethylene in the energy sector increases by 27 Mt, driven by deployment of wind and solar technologies. Solar panels require specialty chemicals such as ethylvinylacetate or polyvinylfluoride, while wind turbine blades require polyethylene terephthalate for core structural components and chemicals for composite materials (e.g., epoxy, polyvinyl chloride, polyurethane) (36). Current demand for aromatics is 116 Mt, including benzene (36%), toluene (23%), and xylenes (40%), and is expected to remain flat until 2050 with downstream mechanical recycling providing an important feedstock.

### Alternative Feedstock, Technology, and Process Energy Options.

Reducing emissions from the chemical sector requires the combined use of alternative feedstocks, renewable energy for direct electrification and green hydrogen, CCS of CO_2_ emissions (37), and efficiency improvements for heating processes to reduce the final energy demand.

**Fig. 2.**
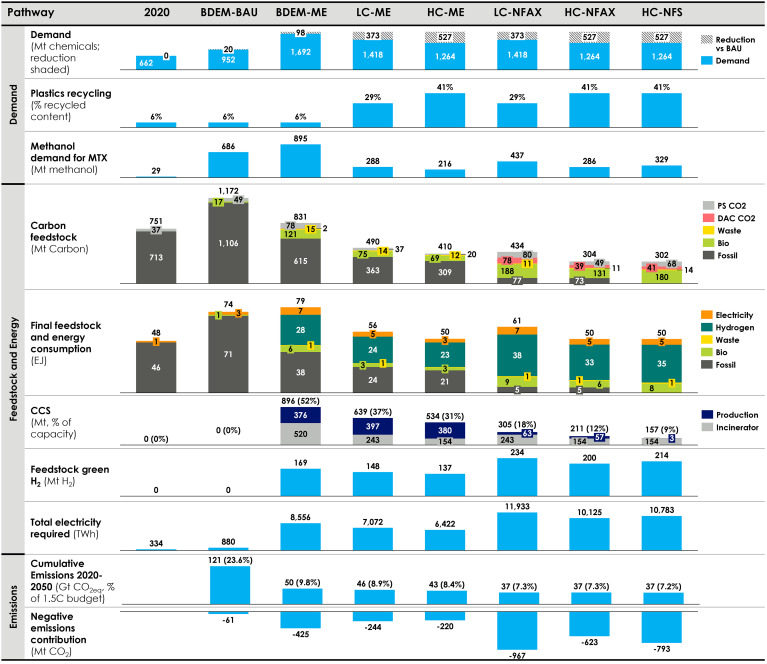
Emission mitigation strategies for all pathways. Demand in the first row excludes methanol demand for MTX to avoid double counting. Reduction of demand in the first row is shaded. Recycled content is different from recycling rates (see *SI Appendix*, section 4.8 for definitions). Electricity for all scenarios besides BDEM-BAU is 100% renewable electricity. BDEM-BAU does not account for new demand associated with energy transition (e.g., ammonia for shipping).

Four supply scenarios are developed: BAU, most economic (ME), no fossil new-build after 2030 (NFAX) and no fossil strict (NFS no fossil new-build after 2025; see *Methods* section and *SI Appendix*, Fig. S14 for more details). Fig. 2 illustrates key results across the presented pathways. Cumulative emissions account for 7 to 24% of the 1.5°C global carbon budget by 2050 (510 GtCO_2eq_, 50% probability of limiting the global temperature below 1.5 °C) (27). The ME pathways (BDEM-ME, LC-ME, and HC-ME) show the chemical industry using 8 to 9% of the 1.5 °C global CO_2_ budget available by 2050 (cumulative GHG emissions of 42 to 47 GtCO_2eq_), the difference resulting from higher levels of resource efficiency and circularity, and more aggressive abatement. This requires 0.45 to 0.85 GtCO2 to be captured in 2050, equivalent to 25 to 50% of the regionally and timely constrained CCS storage capacity available in the model. This constraint was added based on the limited CCS availability today and long timelines for new CCS storage development (see *SI Appendix*, *CO_2_ Storage* section for more details). In the early phase of the transition, the limited availability of CCS severely limits its deployment (*SI Appendix*, Fig. S26). The LC/HC-NFAX and HC-NFS pathways produce only 36 GtCO_2eq_ of cumulative emissions (~7% of the global CO_2_ budget), while lowering the demand for CCS to 0.16 to 0.31 GtCO_2eq_/year.

Today’s chemical system uses >99% fossil feedstocks, but by 2050, this declines to 42% (LC-ME) or more radically to <10% (LC-NFAX and HC-NFAX) or 0% (HC-NFS), reducing cumulative emissions by about 20% (39 to 41 GtCO_2eq_). Naphtha consumption drops most steeply (75 to 95% in LC-ME and LC-NFAX), due to the electrification of road transport and the consequent retirement of catalytic reforming, which produces approximately 85% of aromatics today. This is closely followed by coal (70 to 93% in LC-ME and LC-NFAX), which is disadvantaged in the model due to its higher emissions intensity and costs.

A large fraction (about 90%) of renewable energy used in the chemical industry in 2050 is for the production of green hydrogen, of which the large majority (>98%) is used as feedstock for ammonia and methanol production. Most methanol is converted to other products through MTO/P and MTA. We expect the chemical industry to remain the major producer and consumer of green hydrogen, with up to 250 Mt needed in the no-fossil pathways, equal to 40 to 50% of global consumption in the 1.5 °C aligned 2050 scenario (35, 38). Approximately 250 Mt of sustainable biomass and municipal solid waste are needed in the no-fossil pathways, roughly equal to 40 to 50% of global consumption in the 1.5 °C aligned pathway by 2050 (35, 38), but play a minor role as overall energy carriers ( ≤15% in HC-NFS).

CCU from point source emissions of CO_2_ in industrial plants (e.g., steel, cement) plays a role in all pathways in methanol production. DAC provides a carbon source for the NFAX and NFS pathways and is favored due to its negative emissions footprint and higher abatement potential. Specific technology makeups for each scenario are shown in *SI Appendix*, Figs. S27–S96.

### Emissions and the Opportunity of Carbon Vector Inversion.

**Fig. 3.**
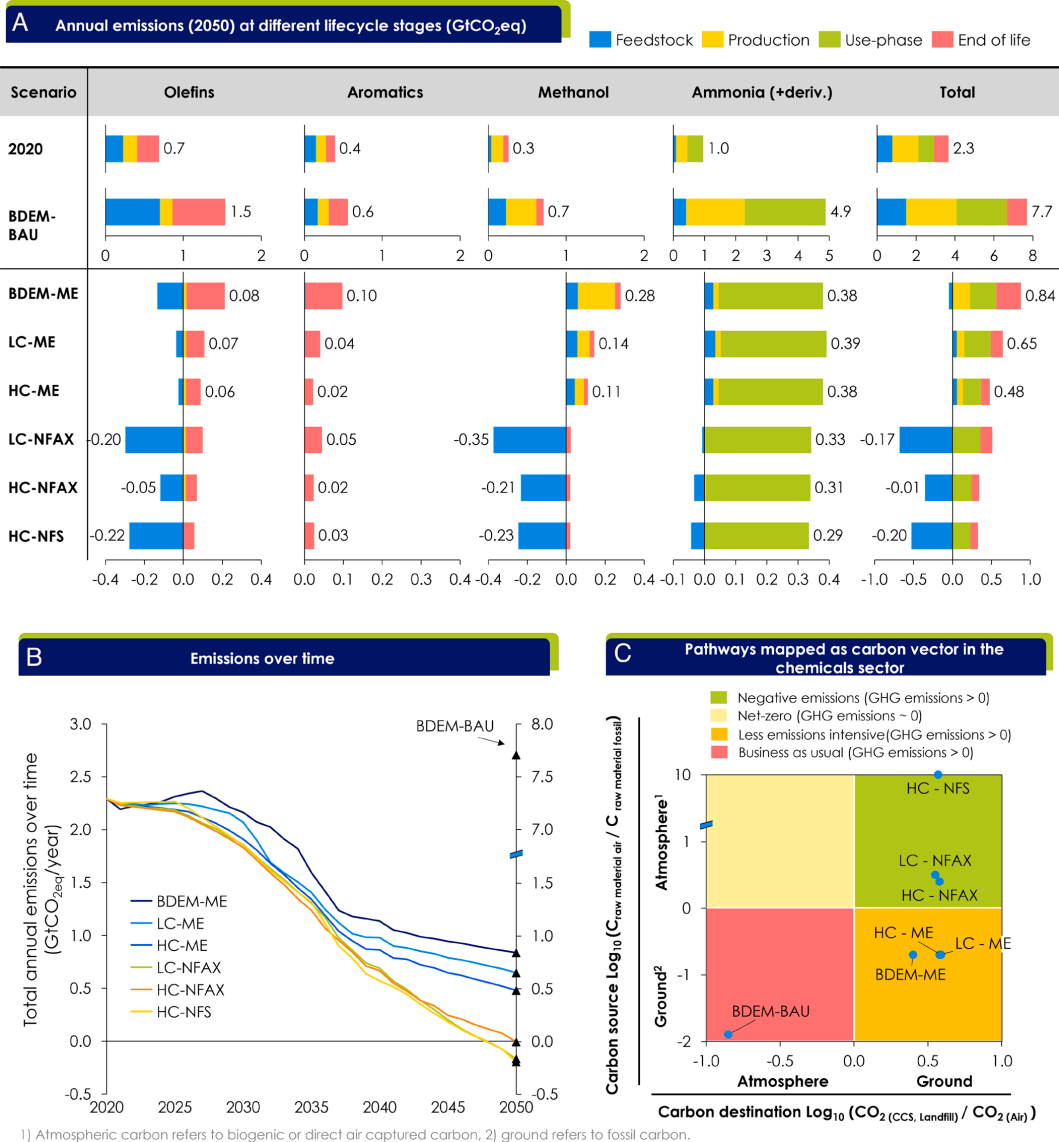
(*A*) Annual GHG emissions of the analyzed chemicals in 2050 over the life cycles for all pathways considered in this study. Use-phase emissions are included for fertilizer but not for plastics; (*B*) Total annual emissions from 2020 to 2050 including GHG emissions associated with fertilizer use-phase. Lower embodied carbon emissions in methanol used in MTA constructed in 2040s lead to diversion of the pathways after 2035. (*C*) Carbon vectors between the ground (fossil) and atmosphere (biogenic or direct air captured carbon) with represented pathways. No pathway is in the top left box because of the assumption on 100% CCS of waste incineration.

Fig. 3*A* shows the shares of GHG emissions across the lifecycle stages in 2050 (feedstock extraction, production, use, and end-of-life) for the four main chemical groups (olefins, aromatics, ammonia, and methanol) and seven featured pathways, compared with 2020. The lifecycle emissions profiles vary significantly across the chemical groups: For example, the use-phase emissions from fertilizer use dominate for ammonia (>90% across all emission reduction pathways), while for olefins, feedstock and end-of-life emissions are most important.

In 2020, the total contributions to annual emissions are: 36% (0.8 GtCO_2eq_ y^−1^) from the production phase; 21% (0.5 GtCO_2eq_ y^−1^) from the feedstock phase; 42% (1.0 GtCO_2eq_ y^−1^) from the use- and end-of-life phases. Fugitive methane emissions are potentially underestimated (39), with some recent studies estimating fugitive emissions to be 25 to 40% larger, presenting a significant climate risk if unmitigated. End-of-life emissions across the industry would increase by up to 70% (2.3 GtCO_2eq_ in 2020) if all plastic waste were incinerated, with or without energy recovery. In this study, it is assumed that by 2050 existing and new waste incineration is abated with CCU or CCS technology.

By 2050, the share of global GHG emissions attributed to chemicals has changed: production emissions are abated by new supply technologies and now contribute only 15 to 24% of lifecycle emissions in the ME pathways (remaining residues due to CCS) and 0 to 2% in the NFAX/NFS pathways. Use-phase emissions of fertilizers cannot be abated fully and contribute the largest share of positive emissions in each pathway in 2050 (27 to 52%)^*^. However, the NFAX and NFS pathways have negative emissions contributions from biomass and DAC, enabling an overall net-negative emissions profile by compensating for the unabated fertilizer use-phase emissions. For example, olefins production today depends heavily on naphtha and ethane for steam cracking. A switch to bio-oils and bioethanol dehydration renders emissions for feedstock production negative for olefins production, with up to 0.2 GtCO_2eq_ removed every year.

Fig. 3*B* shows the trajectories of annual emissions from 2020 to 2050 for all pathways. NFAX and NFS pathways move away from fossil feedstocks and shift end-of-life carbon from atmospheric emissions to sequestration to achieve up to ~0.20 Gt of carbon sequestration per year by 2050. Other pathways, however, are not able to reach full net-zero, largely due to the unmitigated GHG emissions from the fertilizer use-phase.

Fig. 3*C* shows a 2×2 matrix that illustrates the direction of carbon flow (the carbon vector) between the ground and air. Today’s business-as-usual trajectory for the chemical industry (BDEM-BAU) results in carbon flowing from the ground (as fossil fuels) to the atmosphere (as CO_2_ and fugitive methane), as shown in the red square. The most economic pathways (-ME), commonly discussed in the industry today, rely on technologies that return carbon back to the ground (CCS, landfill) to lower emissions intensity. However, the more ambitious pathways (-NFAX and -NFS) require an inversion of the carbon vector, taking carbon from the atmosphere (DAC, bio) and sequestering it in the ground (CCS, landfill) to go beyond net-zero toward carbon negativity.

### Investment Costs.

**Fig. 4:**
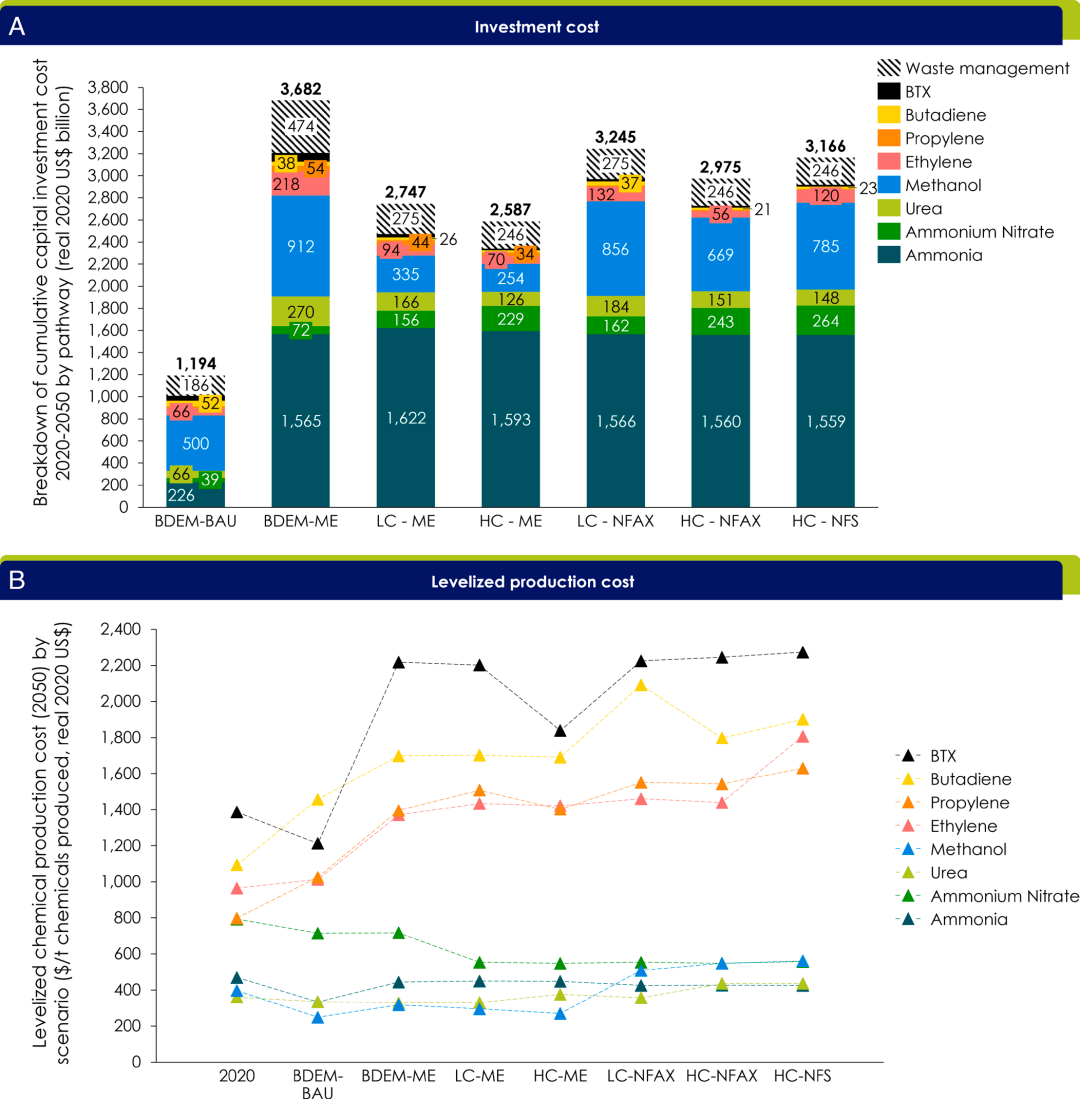
(*A*) Breakdown of capital investment cost of all chemicals and pathways (US$ billion). (*B*) Average levelized production costs of plant stack in 2050 (US$/t).

The cumulative capital expenditure by 2050 to finance the LC-ME and LC-NFAX pathways is anticipated to be US$2.7 trillion and US$3.2 trillion respectively, a 125 to 170% increase above BAU requirements (US$1.5–2.0 trillion). This includes investment costs for renewables for green hydrogen production, which contribute a large share (approximately 50 to 70%) of the total investment costs for green ammonia and methanol. In addition, $0.2 to 0.5 trillion is required in both pathways to manage production and end-of-life waste streams, including collection, sorting, recycling, landfill, incineration, and associated CCS (Fig. 4*A*).

Under the LC-NFAX pathway, the levelized production cost will almost double by 2050. For example, the average cost of olefins will rise from $800 to 1,000 to about $1,400 to 2,000 per metric ton (Fig. 4*B*).

## Discussion

### Balancing Carbon Flows.

**Fig. 5.**
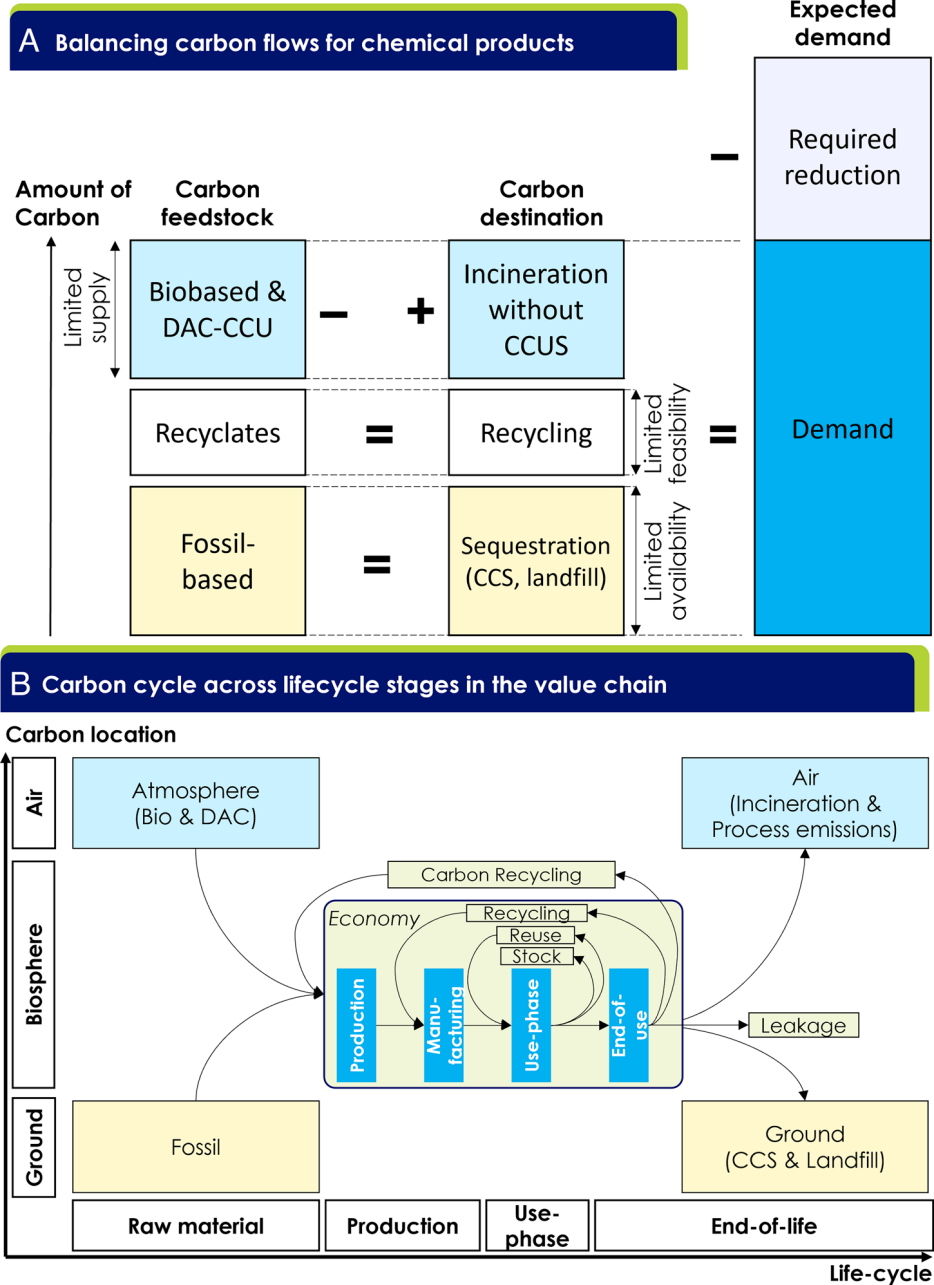
(*A*) Net-zero and carbon balance require the carbon amount in corresponding boxes in feedstock and destination be the same, and together set a limit in the demand. Only CO2 emissions related to embedded carbon are considered (i.e., carbons that go into chemical products of demand). Process emissions are outside the scope of the diagram and need to be dealt with separately by renewable energy, CCS, etc. (*B*) Simplified carbon flows across lifecycle stages in the value chain.

Across all pathways, the key to delivering net-zero emissions lies in balancing carbon flows, a challenge which is unique to the chemical industry, where carbon is embedded in products. Fig. 5*A* shows the comparison of embedded carbon in feedstocks (sourcing and production) and carbon destinations (end-of-life treatments) for chemical products. At each of the three levels, carbon flows need to be balanced to achieve net-zero emissions (*SI Appendix*, section 3). First, the use of fossil-based feedstocks must be balanced with sequestration technologies (CCS of emissions or landfilling of products at end-of-life). CCS allocation to the chemicals industry is limited (assumed to be 470 Mt C or 1.75 GtCO_2eq_ in 2050), placing a cap on the use of fossil-based feedstocks. Second, carbon in discarded chemical products can be recycled as a feedstock for new products. However, collection, sorting, and recycling processes for plastics are imperfect; some plastics are challenging to recycle, and plastic material is degraded during recycling, limiting the available carbon from recyclates (~30 Mt C via pyrolysis, 74 Mt C via municipal waste gasification, 139 Mt C via reuse and recycling). Third, chemical products incinerated at end-of-life release carbon to the atmosphere, so incineration must be discontinued or abated with carbon captured from the air using bio-based feedstocks or DAC/CCU technologies. However, biomaterials are limited in supply (~350 Mt C) and DAC/CCU technologies are costly and both energy- and resource-intensive.

The sum of these limits constrains available carbon for new chemical products (~1,070 Mt C). However, considering practical, technical, and economic requirements to utilize 100% of all these resources, suggest demand reduction to forecasted BAU demand (650 Mt C) to balance the carbon flows may be required. This sets up an explicit tradeoff between bio-based products, recycling, CCS, and demand reduction. Under the assumptions in this study, a 21 to 29% demand reduction for chemical products from BAU facilitates a 9 to 12% reduction in cumulative emissions from 2020 to 2050 (LC, HC).

### Carbon Vector Change—From Air to Ground.

The carbon balance also reveals a unique opportunity for the chemical industry to provide negative emissions, which may offset difficult-to-abate emissions in other sectors. Today’s chemical processes mostly take carbon from the ground and release it to the atmosphere as emissions, creating a ground-to-air carbon vector (Fig. 3*C*). However, the carbon cycle shows a possible route for inverting the carbon vector to air-to-ground by combining bio-based and DAC/CCU technologies with sequestration using CCS or landfill. This offers a new dual value proposition for the chemical industry: utility from chemical products and potentially an additional revenue stream from negative emissions. This unique path for the chemical industry adds significant value to society compared to, for example, direct air capture with carbon storage without the utilization of the carbon atom.

However, relying solely on carbon sequestration technologies to mitigate emissions is risky given their slow development and deployment to date [only ~40 Mt CCS capacity in 2020 (40)]. To mitigate this risk, the industry should also pursue resource efficiency and material circularity solutions (in the center of Fig. 5*B*) to reduce the overall demand for chemical products. Avoiding new virgin production solves several environmental impacts at once, mitigating emissions from extraction, production, and unabated incineration alongside leakage to waterways and marine environments.

### Staying within the Other Planetary Boundaries.

Climate change is only one of the nine planetary boundaries, so the influence of our pathways’ pursuit of net-zero on other processes is examined here. Land-system change is measured by the area (%) of forest land (39, 41), and our pathways set a cap in the biomass availability such that no net conversion of forest takes place. Biochemical flow (nitrogen) is measured by industrial and biological fixation of nitrogen (39, 41). If we assume that the use of ammonia as a fuel returns the fixated nitrogen back to molecular nitrogen, the net fixation of nitrogen in the LC (210Mt y^−1^ as ammonia) and HC- (182 Mt y^−1^) demand pathways in 2050 is less than that in the BDEM pathways (228 Mt y^−1^). Persson et al. highlighted that novel entities, especially plastic pollution, are now considered to have exceeded the planetary boundary (26). Our demand pathways (LC and HC) release 22.1 Mt and 16.3 Mt per year of mismanaged plastic waste into oceans and land respectively, less than BDEM pathways at 35.0 Mt y^−1^. We conclude that the pursuit of net-zero pathways outlined in this study is not at the expense of other planetary boundary processes and is beneficial, especially where demand reduction levers are deployed. The chemical industry could even have a positive impact on planetary boundaries through levers not studied here (e.g., reduced ecosystem exposure to toxic chemicals).

### The Impact on the Cost of Chemicals.

Transformation of the chemical industry will require changes to every single existing asset (decommissioning, retrofit, upgrade) to achieve net-zero. Resulting cost increases in basic chemicals, such as olefins and aromatics, will invariably impact the costs of end-user products, such as automotive and food products. This impact is estimated using an input-output price model and the Leontief inverse matrix for the USA in 2012 (42) and Japan in 2015 (43). The analysis assumes that upstream price increases will be passed downstream and reflected in the price of purchased products without absorbing or inflating the price change, and without changing the production volume or input materials. A 100% cost increase (double the cost) of olefins and aromatics (excluding ammonia and methanol) would cause the production cost of an automobile to increase by 1.1% and 1.0%, and (frozen) food products to increase by 0.9% and 0.6% in the USA and Japan, respectively. Doubling the cost of olefins and aromatics does not translate to significantly higher costs of end-user products due to inputs and value added from all sectors in the supply chain (44). The implication is that the chemical industry should not delay the transition to net-zero, as any cost increase in primary chemicals will have an almost negligible impact on the production cost of end-user products. (*SI Appendix*, section 7).

### Recommendations.

This study has demonstrated that achieving net-zero emissions across the lifecycle stages for chemical products is possible but requires the deployment of both supply- and demand-side interventions. The chemical industry should accelerate the development of technologies highlighted in this study (non-fossil feedstocks as well as CCS, MTO/P, and MTA), and collaborate with other industries to drive resource efficiency and material circularity, reduce demand, and balance the carbon flows. Policies such as carbon pricing and mandates to bridge the cost gap across the supply chain are essential to accelerate investment in the chemical industry. Additional revenue streams from negative emissions may offer a new value proposition for the industry. Demand-side coalitions and changes to government purchasing can help drive future demand for net-zero chemicals. In conclusion, this work highlights planet-compatible pathways for the chemical industry based on circular strategies and a new system service for sequestering carbon, while continuing to provide essential services to society.

## Materials and Methods

This study aims to identify and outline the key conditions for the chemical industry to reach net-zero GHG emissions along planet-compatible pathways between 2020 and 2050. Emissions from feedstock sourcing, production, use phase, and end-of-life emissions are considered (*SI Appendix*, Table S1). We construct a dynamic production facility supply model, based almost exclusively on open-source data, which responds to different inputs (e.g., cost, carbon abatement, feedstock) and 50 production technologies (*SI Appendix*, Fig. S19), across 10 geographic regions. We focus on eight primary chemicals: ammonia, methanol, olefins (ethylene, propylene, butadiene), aromatics (benzene, toluene, xylene), and two derivatives (ammonium nitrate, urea) that currently account for ~72% of global chemical industry production GHG emissions, two-thirds of energy used, and ~82% of primary chemical production by mass.

### Planetary Boundaries and Resource Availability.

The planetary boundaries framework identifies human perturbations in nine Earth system features that if transgressed will threaten the safe operating space for global societal development (41). This study selects climate change (GHG emissions) as a key modeling constraint, and considers available biomass (at 10 EJ or 720 Mt dry biomass for chemical use in 2050, ~20% of global availability) to safeguard land-system change and biodiversity (45). Impacts on novel entities [e.g., plastic pollution into environment (26)] and biochemical flows (nitrogen) are assessed outside the main model.

For each chemical, we define planet-compatible pathways to minimize demand for new chemical production and life cycle GHG emissions by 2050, with demand and supply strategies modeled concurrently. Resource efficiency and materials circularity (i.e., elimination, reuse, recycling, and substitution)^†^ are presumed as key enablers for reducing demand for virgin chemicals and related emissions.

### Demand Scenarios.

The business-as-usual demand (BDEM) scenario for chemical products is based on IEA (International Energy Agency) Reference Technology Scenario (RTS) projections (1, 5) plus additional demands for chemical products (ammonia for shipping and power generation; olefins for solar and wind deployment and electric mobility) required for net-zero pathways in other sectors (38, 46).

Two demand scenarios are created based on BDEM with more ambitious shifts in end-use chemical demand: HC based on the maximum potential implementation of resource efficiency and circularity levers^‡^, LC at 50% of the maximum potential (except mechanical recycling using same absolute market size). Increases in resource efficiency and material circularity are considered for fertilizers, packaging, household goods, transportation, construction, and textiles, covering 87% of the chemical production studied here. For example, mechanical recycling is capped at optimistic 20 to 60% rates depending on plastics and sectors; see *SI Appendix*, section 4, Figs. S2–S12, and Tables S2–S8.

HC and LC scenarios assume significant improvements in waste management (collection, sorting), end-of-life infrastructure (landfill, incinerators, CCS for incineration), and fertilizer application and use efficiency. End-of-life emissions are calculated based on the demand scenarios (21).

### Supply Scenarios.

Only technically mature (>Technology Readiness Level 6) chemical production technologies are modeled to ensure pathways are technologically and financially realistic and immediately implementable. Evolving caps are applied to feedstocks and resources, including sustainable biomass (45) (increasing from 2 to 12 EJ in 2050 including municipal solid waste; 25% of share for chemical industry of global availability); polymer waste as feedstock for pyrolysis oil (~40 Mt y^−1^); treated wastewater (155 Gt y^−1^); and CO_2_ storage (increasing from 0.04 to 1.75 Gt in 2050; 25% share for chemical industry of seven Gt global CCS demand (47)). These limits are developed alongside projected demand in other sectors (e.g., aviation and steel) based on the IEA RTS scenarios and analysis by the Mission Possible Partnership. CCS is limited by a lack of current commercial-scale storage facilities and CO_2_ transport network, both of which have significant development timelines. In addition, localized geographical availability of underground CO_2_ storage hinders widespread deployment, limiting build-out especially in the next 10 to 15 y (*SI Appendix*, Fig. S26). Electrification, decarbonization of the power system and green hydrogen as a feedstock/storage medium underpin net-zero pathways for all sectors, and are not capped in the model due to shorter development timelines and no inherent physical or geographical limitations which could hamper rollout (e.g., minerals, land availability) (35, 38, 46, 47).

BAU selects the ME technologies (including unabated), based on levelized cost of production, to meet demand, without considering emissions reduction. For all scenarios, unabated fossil technologies can be built until 2025, beyond which only abated technologies (ME, NFAX, NFS) are allowed. NFAX builds no new fossil feedstock or energy plants after 2030 (even with CCS), while NFS, in addition, prevents any fossil-based feedstock or energy plant after 2025 and retires all fossil-based plants by 2050. An assumed fixed retrofit rate (5% y^−1^ globally) is applied to convert or retire existing assets, with new plants optimized for the lowest cost (ME) or largest GHG lifecycle emissions abatement (NFAX, NFS; see *SI Appendix*, section 5 and Figs. S13–S26).

### Supply and Demand Pathways.

**Fig. 6.**
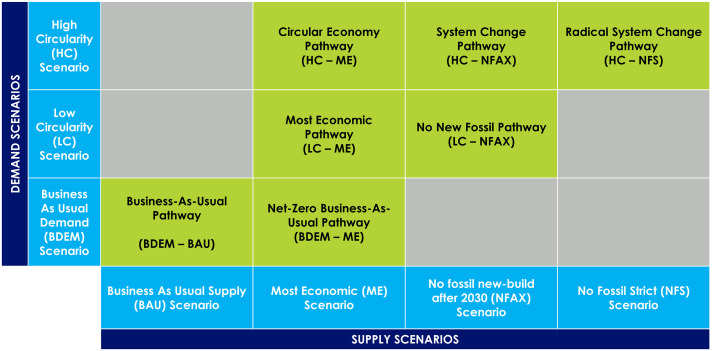
The seven featured planet-compatible pathways (green) combining demand and supply scenarios (blue). Detailed descriptions of each scenario can be found in *SI Appendix*, Fig. S14.

This study features seven planet-compatible pathways (shown in Fig. 6) selected as the most interesting pathways to compare and contrast. These pathways are not forecasts, but describe what needs to happen, based upon the best available data today, to shift toward net-zero using different approaches. The pathways do not consider national infrastructure, trade, or energy security. Cumulative investment costs and levelized production costs (capital expenditures and operating expenses discounted to a present value per unit of production) are calculated using plant-level modeling for each pathway between 2020 and 2050.

## Supplementary Material

Appendix 01 (PDF)

## Data Availability

All data are publicly available (48). All data and codes that do not require an additional license are archived at GitHub: https://github.com/systemiqofficial/Pathways-Chemical-Industry.

## Footnotes

^*^Strategies for reducing use-phase fertilizer emissions include precision agriculture, using nitrification and urease inhibitors and substituting urea, urea ammonium nitrate, and ammonium bicarbonate, with ammonium nitrate.

^†^The strategies of resource efficiency and circularity are defined inconsistently across literature. In this paper, we use both terms interchangeably, in their broadest sense, to cover solutions including elimination, reuse, recycling and substitution. Both resource efficiency and circularity strategies lead to reduce demand for virgin chemical production.

^‡^There remain two large uncertainties associated with demand scenarios: 1) the baseline demand used from IEA may not be considered socially acceptable as the plastic and fertilizer consumption per capita in 2050 still includes large differences between countries and 2) the risk that, due to falling demand, oversupply from some production processes may remove local incentive for further demand reduction.
