# Supplementary material for: Planet-compatible pathways for transitioning the chemical industry
Source: Proc Natl Acad Sci U S A. 2023 Feb 14;120(8):e2218294120. doi: 10.1073/pnas.2218294120 (PMC9974437; doi:10.1073/pnas.2218294120)
Supplement: Supplementary file 1 — Appendix 01 (PDF) [file pnas.2218294120.sapp.pdf]

PNAS

Supporting Information for

Planet compatible pathways for transitioning the chemical industry

Fanran Meng[1]†, Andreas Wagner[2]†, Alexandre B. Kremer[2]†, Daisuke Kanazawa[5]†, Jane J. Leung[2], Peter Goult[2], Min Guan[2], Sophie Herrmann[2], Eveline Speelman[2], Pim Sauter[2], Shajeeshan Lingeswaran[2], Martin M. Stuchtey[2,6], Katja Hansen[7], Eric Masanet[,8,9], André C. Serrenho[1], Naoko Ishii[5], Yasunori Kikuchi[3,4*], Jonathan M. Cullen[1*]
[1]Department of Engineering, University of Cambridge, Trumpington Street, Cambridge CB2 1PZ, UK
[2]Sytemiq, 69 Carter Lane, London EC4V 5EQ, UK
[3]Department of Chemical System Engineering, The University of Tokyo, 7-3-1 Hongo, Bunkyo-ku, Tokyo, 113-8656, Japan
[4]Institute for Future Initiatives, The University of Tokyo, 7-3-1 Hongo, Bunkyo-ku, Tokyo, 113-8654, Japan
[5]Center for Global Commons, Institute for Future Initiatives, The University of Tokyo, 7-3-1 Hongo, Bunkyo-ku, Tokyo, 113-8654, Japan
[6]Faculty of Business and Management. Innsbruck University, Universitätsstraße 15, 6020 Innsbruck, Austria
[7] Institute of Energy Efficient and Sustainable Design and Building, Technical University of Munich, Arcisstr. 21, 80333 München, Germany
[8]Bren School of Environmental Science and Management, University of California, Santa Barbara, CA, United States of America
[9]Department of Mechanical Engineering, University of California, Santa Barbara, CA, United States of America

*Corresponding authors. Email: jmc99@cam.ac.uk; ykikuchi@ifi.u-tokyo.ac.jp
†These authors contributed equally to this work.

This PDF file includes:

Materials and Methods
Figs. S1 to S97
Tables S1 to S12
SI References

Other supporting materials for this manuscript include the following:

https://github.com/systemiqofficial/Pathways-Chemical-Industry

Supplementary Methods & Discussion 3
1 Supporting information files 3
2 Performing analysis with provided files 4
3 Defining a planet-centric chemical industry and key differences to other models and scenarios 4
4 Demand Analysis 8
5 Supply Analysis 15
6 Limitations to model and suggestions for future improvements 26
7 The impact of the cost of chemicals 27
Supplementary figures 29
Supplementary Tables 126

Supplementary Methods & Discussion

Supporting information files

The Python model for supply analysis alongside its associated inputs and outputs are all published open source alongside the manuscript. The Excel based demand model is published on the same GitHub repository. The availability of open-source data is particularly limited for the chemical industry with plant-based and techno-economic data mostly unavailable or hidden behind expensive paywalls. It was therefore a critical part of this project to create an open model with transparent inputs.

The following files form part of the supplementary information and are referred to at different stages:

1) Python model for the supply part of the model published on GitHub
2) “Master template.xlsx” containing all input for the supply side model including techno-economics and emissions data
3) “Demand model.xlsx” entails all demand side related modelling including the end-of-life treatment model
4) “Model outputs.xlsx” contains the consolidated outputs of the Python model
5) “Global Dashboard.xlsx” plots and interprets the Python model outputs and derives key statistics and insights

The GitHub repository can be found here: https://github.com/systemiqofficial/Pathways-Chemical-Industry

The output files are stored in the “/output” folder and input file in the “/data” folder, respectively.

Performing analysis with provided files

The following step-by-step instruction allows to compute the model:

1. Download Python to your computer. The model was developed for Python version 3.9.7.
2. Download a script editor. There are several options, such as PyCharm or VSCode
3. Download GitHub Desktop
4. Register for a GitHub account
5. Clone the repository (https://GitHub.com/systemiqofficial/chemicals-decarbonization.git) to a local folder of your choice via "File-clone a repository".
6. Open python editor and load the project
7. Navigate to `chemicals-decarbonization` folder
8. Set up a virtual environment and activate it by entering the following commands into terminal:

Option 1) using `virtualenv`

- py -m pip install
- `Pip install virtualenv`
- `python3 -m venv venv`
- `venv\Scripts\activate`

Option 2) using conda

- `conda create -n chemicals-decarbonization python=3.9`
- `conda activate chemicals-decarbonization`

9. Install project requirements using `pip install -r requirements.txt`
10. Make a copy of the config_template.py file and rename it to config.py. Make changes only to config.py if any input parameters should be changed
11. Type in the terminal: `python main.py` to run the model
12. Outputs are added in the output/ directory

The Python model produces a comprehensive summary of output variables ("Model outputs") which are analyzed in Excel in the "Global Dashboard" file. In the sheet "DashDash", the sensitivity can be chosen. The five different supply scenarios (business-as-usual (BAU), most economic (ME), no fossil new-build after 2030 (NFAX) and no fossil strict (NFS), fastest abatement (FA)) (see section 5) for the given pathway are then loaded in the "Output" sheet. To enable the loading of data and all associated functions, the "Global Dashboard" file must be opened alongside the "Model outputs" and "Demand Model" files. All files need to be stored in the same folder.
The config.py folder can be modified to change a wide variety of assumptions (Section 0). In particular, the model can run on multiple CPU cores in parallel to accelerate the runtime by setting "RUN_PARALLEL" to True. Typical run-time for one scenario-sensitivity combination is ca. 25 min.

Defining a planet-centric chemical industry and key differences to other models and scenarios

While there are multiple possible interpretations of how the chemical industry can be aligned with a net zero future, a few key principles are adopted in this work that we believe offer the necessary systems-level change required to achieve this in a planet-compatible manner. We construct a dynamic plant-based supply model that can respond to different inputs (e.g., cost, carbon abatement, feedstock), drawing from ~50 production technologies split across 10 global regions between 2020 and 2050 (Section 0). We examine eight primary chemicals (Ammonia, Methanol, Ethylene, Propylene, Butadiene, Benzene, Toluene, Xylene) and two derivatives (Ammonium Nitrate, Urea) over the full life cycle stages. While environmental pollution and the impact on the health of human and other species are both of major concern, they remain out of scope of this work and are covered in other studies (1, 2).
The study features seven main supply-demand pathways which are identified as most relevant from the 25 scenarios computed. The supply scenario called "fastest abatement" is not discussed

in more detail as it is not included in the main manuscript given its similarity to the NFAX scenario outputs.
The model is distinctive compared to other analysis in the literature alongside the following dimensions (3–8):

- An agent-based supply model, in which an agent represents a manufacturing plant and externally forcing mechanisms are used to enable greenhouse gas (GHG) emissions towards net zero (described in more detail below and in Section 0).
- Wide array of decarbonization business cases including several but not yet commercially available technologies (but above technology readiness level (TRL) 6) based on almost exclusively open-source data (Section 0).
- Inclusion of raw material production emissions (fugitive emissions) and end-of-life emissions (Section 0).
- A set of limitations to regional and temporal availability of carbon capture and storage (CCS), sustainable biomass (including sub caps for e.g., bio-oils), municipal solid waste and pyrolysis oil (Section 0).
- Assessment of maximum circularity & demand reduction strategies coupled with supply of primary chemical production (Section 0).
- An intra-chemical linkage between methanol and other chemicals in which the methanol feedstock consumption for MTX processes is considered as new demand for methanol production year-by-year (Section 0).

<u>What is meant by net zero and how do we get there?</u>
"Net zero-carbon-emissions", "net zero-carbon", "net zero": these terms are used interchangeably to describe the situation in which the energy and industrial system as a whole or a specific economic sector releases no GHG emissions (quoted as CO_{2eq}) – either because it does not produce any or because it captures the CO_2 it produces to use or store. In this situation, the use of offsets from other sectors ("real net zero") should be extremely limited and used only to compensate for residual emissions from imperfect levels of carbon capture, unavoidable end-of-use emissions, or remaining emissions from the agriculture sector. This includes specifically, feedstock (scope 3 upstream), use-phase for fertilizer (scope 3 downstream) and end-of-life of plastics (scope 3 downstream).
<u>GHG emissions categories shown in lifecycle stages (this study) and GHG Protocol's categories</u>
This study categorized GHG emissions based on lifecycle stages using general terminology. They are feedstock extraction, production of chemical products, use-phase of chemical products, and end-of-life treatment of chemical products. GHG emissions have also been categorized by GHG Protocol under different scopes and categories as GHG accounting standards for corporate reporting purposes (9).
Table S1 compares the categories used in this study with GHG Protocol's categorization.

<u>Volume reduction complementary with GHG reduction of production: how supply and demand are modelled hand-in-hand</u>
A key hypothesis of the modelling is that resource efficiency through materials circularity is a key enabler to optimize the chemical industry system size and thus reduce demand for virgin chemical products. It is also critical that considerations from both angles – demand and supply – are taken simultaneously and married together in our analysis. For each chemical, we therefore look at defining pathways to maximize resource efficiency and minimize absolute emissions of scopes 1, 2 and 3 by 2050 from the perspective of changes in demand and supply.
The approach consists of two general phases: 1) demand scenarios that are then met by 2) back-casted optimized production scenarios (more detailed methodology on each of these 2 sets of analyses is elucidated in sections 3.4 and 3.55).

1. Demand for each chemical in a 2050 post-transformation world is the combination of the following:
    - Business-as-usual demand (BDEM) from IEA projections for 2020 to 2050. This demand baseline is used for BDEM-BAU pathway.
    - Demand of new applications in a net zero world (e.g., ammonia for shipping, electrification, solar and wind development) based on

decarbonization pathways for relevant sectors. This demand baseline is used for BDEM-ME and BDEM-NFAX pathways.
    - Different levels of circularity (elimination, reuse, recycling and substitution) in existing applications are taken into account. This demand baseline is used for all low circularity (LC) and high circularity (HC) pathways.
    - Ranges in assumptions in the above are captured to finally generate both high- and low- demand scenarios for each chemical corresponding to different levels of circularity.
2. Production scenarios to meet the high demand scenarios for each chemical by net zero carbon manufacturing technologies are then assessed:
    - Techno-economic assessment of net zero production technologies for each chemical (TRL > 6 only)
    - Broader system implications and how to consider them cross-chemically: technology maturity of key enabling technologies (e.g., CCS, green H_2), resource limitations (e.g., CCS, sustainable biomass).
    - Simulation scenarios below take all the above into account to optimize according to a different parameter set and within different constraints.

Geographical and trade considerations

The demand is modelled on a global basis with no regional breakdown whereas the supply model is broken down into 10 regions listed below. The sum of all regional production must meet global demand. The decision to limit the demand on a global level is based on two factors: On the one hand, demand forecast in different regions is very challenging due to its dependence on many different factors including economic development and the role of local policy. On the other hand, many of the chemicals analyzed in this study (Ammonia, Urea, Ammonium nitrate, Methanol, Benzene, Toluene, Xylene, Butadiene) can be transported relatively easily and are partly already being traded internationally today. In addition, shipping costs are typically small compared to the price of chemicals, and even significantly smaller than the difference between low and high-cost regions, making international trade economically attractive.

Regional breakdown of supply model:

- North America
- Latin America
- Europe
- Russia
- Africa
- Middle East
- China
- India
- Japan
- Rest of Asia and Pacific

Remaining within planetary boundaries is paramount

While quantifying impact on all planetary boundaries is difficult – both as a result of limited data availability and lack of standardized metrics by which to carry out this exercise – we have nevertheless attempted to maintain caps on system inputs and outputs in such a way to evaluate the climate change while respecting a few other key planetary boundaries.

These include:

- **Impact on land use and biodiversity:** a cap on available, sustainable, low-emission biomass supply as feedstock for the chemical industry is employed. This cap is calculated to take into account: no competition with other critical uses of land, no deforestation or peatland conversation, target degraded land with little plant growth, respect growth periods (10). As a result, this biomass cap allows to draw pathways for the chemical industry while remaining within planet boundaries associated with land use changes as well as biodiversity loss.

- **Impact on biosphere and nitrogen flows:** the reduced use of fertilizers recommended in low circularity and high circularity scenarios (11, 12) will mechanically limit runaway impact on the biosphere by equivalent amounts.
- **Impact on novel entities release:** waste management systems are analyzed in the context of end-of-life pathways. The combination of reduced plastic usage estimated in this model and improved waste management scenarios estimated from Breaking the Plastic Wave model (13), suggests that, with ambitious development of such levers, environmental leakage (including both leakage on land and in the ocean) can be effectively reduced by more than 80% by 2050, similarly microplastic pollution can be reduced by 78%.

<u>Balancing carbon flows</u>

This section explains balancing carbon flows from section 1.4.1 in the main manuscript in more detail (Supplementary figures

Fig. S1.).

This model analyzes the relationship in the amount of carbon atoms embedded in feedstock, chemical products, and end-of-life destinations. It does not consider emissions such as N_2O from fertilizer use, CH_4 from landfill, or the upstream CO_2 /CH_4 from fossil extraction. It further assumes that direct emissions and indirect electricity emissions (scope 1 & 2) from chemical processing to be net zero.

Under these assumptions, carbon balance requires $A + B + C = D = E + F + G$, where A through G means the following:

A: amount of carbon embedded in the fossil-based feedstock for chemical products,
B: amount of carbon embedded in the recycled feedstock for chemical products (including CCU),
C: amount of carbon embedded in the biobased and DAC-CCU feedstock for chemical products,
D: amount of carbon in the demand for chemical products,
E: amount of carbon from chemical products' end-of-life treatment that is sequestered,
F: amount of carbon from chemical products' end-of-life treatment that is recycled into feedstock,
G: amount of carbon from chemical products' end-of-life treatment that is incinerated without CCU or CCS.

By definition, $B = F$ (where recycling loss is included in E or G), therefore $A+C = E+G$. Further, net zero requires $C = G$, therefore $A = E$. Thus, the boxes with the same color must have the same carbon amount, in order for CO_2 emissions from end-of-life treatment to be net zero, or in order for Scope 3 category 12 (end-of-life treatment) emission to be net zero for CO_2. It is important to note that not all *(A)* fossil-based feedstock needs be *(E)* sequestered, and not all *(C)* biobased & DAC-CCU feedstock needs be *(G)* incinerated without CCUS in order to be net zero. Some *(A)* fossil-based feedstock can indeed be *(G)* incinerated without CCUS, and some *(C)* biobased & DAC-CCU feedstock can be *(E)* sequestered, and still the system can remain net zero, as long as the carbon amount equality ($A=E$, $B=F$, $C=G$) is maintained.

Because each of *(C)* biobased & DAC-CCU, *(E)* sequestration, and *(F)* recycling has limitation in size as discussed in 1.4.1, *(D)* demand that the chemical industry can accommodate under Scope 3 category 12 net zero also has limitation in size, since $D = C + E + F$.

As discussed earlier, this model excludes some of the chemical industry's life cycle (scope 3) emissions such as N_2O from fertilizer use, CH_4 from landfill, and the upstream CO_2 / CH_4 from fossil extraction. Taking these emissions into account will place further limit on the demand.

This model assumes in addition that the annual demand is at a steady level, resulting in a steady level of stock of embedded carbon in the chemical products in the economy, and that the recycling of carbon takes place only within the chemical industry's supply chain.

Demand Analysis

Aim

The demand model analyses what is the demand for the eight primary chemicals and two derivatives and quantifies the resource efficiency and circularity strategies[1] which impacts each of the key demand sectors as well as additional demand arising from meeting net zero targets. This model is based on the principles of resource efficiency and circularity for demand reduction. The analysis calculates the annual demand in the period 2020-2050 under different scenarios as described below.

The demand model is a deterministic and linear model and unlike the supply model does not include optimization features. The model is based on existing knowledge of resource efficiency and circularity strategies and their potential development (e.g., market penetration) by 2050 in each of the key sectors studied. Additional demand arising from climate transition is estimated assuming all other sectors achieve net zero by 2050.

The model output is therefore not a predetermination, projection or forecast of any kind, but rather a simulation that offers a view on key elements of the industry's direction of travel across the next 30 years if downstream industries and adjacent industries achieve net zero. It includes three scenarios: business-as-usual demand (BDEM), low circularity (LC) and high circularity (HC). Noteworthy, BDEM for abated supply scenarios (BDEM-ME, BDEM-NFAX) includes new demand associated with net-zero whereas BAU supply does not (BDEM-BAU). Insights possible from this model includes flows of chemicals studied produced from each key industry and abated by each of the levers studied, emissions arising from the end of life of each chemical as well as cost. Emissions and cost from resource efficiency and circularity strategies other than end of life (i.e., collection, sorting, recycling, end-of-life treatment), is excluded.

Model Map

The model follows a stepwise approach (Fig. S2)

1. Annual chemical demand projections using IEA RTS and STEPS scenario data(3, 12)
2. Annual chemical demand breakdown into tiers 1/2/3/4 chemicals and for each of the key industry sectors (1) fertilizers, (2) household goods, (3) buildings and construction, (4) transportation, (5) textiles, and (6) energy, for 2020 and assumed constant to 2050. Tiers chemicals are defined are chemicals which are 1/2/3/4 chemical reacting steps away from primary chemicals (e.g., primary chemical: ethylene, tiers 1: ethylene oxide, tiers 2: ethylene glycol, tiers 3: PET).
3. Calculation of the impact of key resource efficiency and circularity strategies including direct elimination, reuse and substitution to demand for each of the key industries.
4. Calculation of the additional demand arising from adjacent sectors reaching net zero (e.g., shipping fuel, wind power and solar panel materials)
5. Mapping of plastic waste management flows and end-of-life treatment options between 2020 and 2050 including collection, sorting, recycling, landfilling, incineration, dumping, open burning and leakage (to the land environment and to the ocean).
6. Calculation of the recycling levers including mechanical and upstream chemical recycling (e.g., depolymerization and solvent-based recycling technologies but excluding pyrolysis and gasification which are included in the supply model) for each of the key industries.
7. Calculation of waste management cost and associated end-of-life emissions.

Note that each demand scenario also includes an end-of-life plastic waste management scenario. Business-as-usual (BDEM) assumes historical growth for collection, sorting, recycling, and end-of-life treatment. High and low circularity (HC and LC, respectively) assumes similar ambitious growth for collection and sorting and end-of-life treatment. Recycling for those two scenarios is delt separately and discussed below.

Demand in 2020 to 2050 in BAU

Chemical demand projections for BAU are made using the following sources and extrapolated to 2020 when necessary:

---

[1] The strategies of resource efficiency and circularity are defined inconsistently across literature. In this paper, we use both terms interchangeably, in their broadest sense, to cover solutions including elimination, reuse, recycling and substitution. Both resource efficiency and circularity strategies lead to reduce demand for virgin chemical production.

- Reference technology scenario (RTS) from IEA for ethylene, propylene, methanol, BTX(i.e., benzene, toluene, xylene) and C4 (e.g., butadiene) (3).
    - BTX are further broken down into benzene, toluene and xylene (14).
    - C4 are further broken down into butadiene, 1-butene, 2-butene, isobutene (14).
- Stated policies scenario (STEPS) from IEA for ammonia (12).

Demand of each of the eight-chemical studied (ammonia, ethylene, propylene, methanol, benzene, toluene, xylene and butadiene) is then broken down into tiers 1/2/3/4 chemicals using various publicly accessible sources. Specific data used for each chemical is accessible in the "Demand Map" excel file shared in the orange tabs labelled 'Demand Breakdown – X'.

The share of each chemical breakdown is assumed constant to 2050. An example for ethylene is shown in Fig. S3.

<u>Sectorial Demand in 2020 to 2050 in BAU</u>

Sectorial analysis is then performed to estimate the share of chemical usage per industry using plastic as a driver (15). Data from Geyer et al. is used and adjusted to reflect low rubber content and to include synthetic fibers (i.e., textiles) – 83.3 Mt in 2019 (16). Molar mass of each chemical is used to calculate the chemical-derived mass from each sector for 2020.

2050 sectorial demand projections are calculated based growth from several industry report and adjusted to match overall IEA projections.

All data, calculation and assumptions made are accessible in the "Demand Map" excel file shared in the orange tabs labelled 'Demand Breakdown – X' and 'Demand – Industry shares'.

An example for ethylene is shown in Fig S4.

For ammonia, sectorial demand is divided between fertilizer (71.2% in 2020 and 69.5% in 2050)(12), and others.

<u>Additional Demand to 2050 in a Net Zero World</u>

Additional demand, meaning demand from sectors which is not accounted in the IEA reference scenarios, is estimated and added to existing demand projections for LC and HC scenarios. Same assumptions have been used for both scenarios which exclusively include additional demand from the energy sector to achieve net zero:

- **Ammonia demand for shipping:** the analysis assumes that ammonia for shipping is the only technology to bring shipping to net zero by 2050. The demand for ammonia will therefore considerably grow to overtake fuel production from 0 Mt in 2020 to 625 Mt in 2050. Estimations of ammonia required to power the shipping industry by 2050 are made by UMAS (a University of College London Initiative) and MPP (a World Economic Forum Initiative) and accessible to the authors on a yearly basis (17). Data series is accessible in the 'Ammonia Demand Map' excel file, under the tab 'shipping'.
- **Ammonia demand for power generation as an energy carrier:** Based on examples from Japan and Korean governments' commitment to use ammonia firing power plant as several industry players(18, 19), it is estimated that ammonia demand for power generation could be as high as 160 Mt by 2050. A linear growth is assumed between 2020 and 2050. Data series is accessible in the 'Ammonia Demand Map' excel file, under the tab 'Power generation'.
- **Chemical demand for wind turbines:** the analysis assumes that wind turbines capacity will increase from 0.7 MW in 2020 to 15 MW in 2050.(20) Wind turbines are made of various polymer composites (4%) including epoxy, PET, PVC, PUR.(21) Associated additional demand for each chemical is estimated based on polymer composite composition, molar mass and growth in wind turbine capacity. Assumptions and data series are accessible in the 'Demand Map' excel file, under the tab 'Demand – Wind'.
- **Chemical demand for solar panels:** the analysis assumes that solar panel capacity will increase from 0.7 MW in 2020 to 31 MW in 2050.(20) Solar panels are made of various chemicals including ethyl vinyl acetate and poly (vinyl fluoride).(21) Associated additional demand for each chemical is estimated based on chemicals composition, molar mass, and growth in solar panel capacity. Assumptions and data series are accessible in the 'Demand Map' excel file, under the tab 'Demand – Solar'.
- **Chemical demand for battery:** focusing on battery electric vehicles specifically, the demand for plastics in cars is expected to rise by 10% from the switch from internal

combustion engines (*16*). Extrapolating from this, the additional chemical demand from the transportation system is assumed to rise by 10% non-linearly from 2020 to 2050; 1% by 2030, 5% by 2040 and 10% by 2050 based on expected penetration rates of battery electric vehicles (20). This rate is assumed constant across all relevant chemicals for the sector.

Reduction and Substitution Levers

Impact of circular business models on chemical demand are estimated using existing report and publications for each of the key industry sector and for each chemical separately.

- **Agricultural sector:** *(for ammonia only)*
    - Fertilizer reduction is the only lever consider although it encompasses four discrete sub-levers: improved agricultural practices (e.g., land management), precision agriculture (e.g., new technology), diet change (e.g., reduce meat consumption) and reduce food waste. LC scenario is obtained from IEA net zero emissions scenario (NZE - 11% reduction potential by 2050, corresponding to 18 Mt) (12). HC scenario is obtained from GLOBIUM model developed by the Food and Land Use Coalition using the better future scenario (BF - 29% reduction potential by 2050, corresponding to 46 Mt) which model is provided to the authors (11). Data series are available in the 'Ammonia Demand Map' excel file, in the tabs labelled 'Annual Ammonia HD' and 'Annual Ammonia LD'.
    - Fertilizer substitution is assumed although it does not affect overall ammonia demand that it changes the mix of fertilizer use by 2050. In 2020, urea represent 55% of fertilizer on a nitrogen basis while ammonium nitrate represents only 11% (total 66% on an N-basis) (12) (IEA analysis is based on International Fertilizer Association database). LC scenario is obtained from IEA net zero emissions scenario (NZE) and by 2050 urea represents 22% of the fertilizer mix and ammonium nitrate 44% (total 66% on an N-basis). HC scenario is obtained assuming full substitution of urea for ammonium nitrate given urea displayed higher scope 3 emission factors compared to other fertilizers available (22). Substitution effect is assumed linear between 2020 and 2050. Data series are available in the 'Ammonia Demand Map' excel file, in the tabs labelled 'Annual Ammonia HD' and 'Annual Ammonia LD'.
- **Packaging and household goods:** *(for ethylene, propylene, methanol, benzene, toluene, xylene and butadiene)* Four levers, respectively elimination, reuse, paper substitution and compostable substitution, are considered (13). Data is extrapolated from 2040 to 2050 using data provided by the authors of Breaking the Plastics Wave. Their analysis is drawn based on 18 plastic product categories (e.g., beverage drinks, sachets, trays etc.) which is then broken down at the polymer level (e.g., PET, PE, PP etc.) to access chemical composition (e.g., ethylene, propylene etc.). Dataset obtained is triangulated and adjusted using Plastics Europe dataset which offers a polymer breakdown by industry (23). The modeling results in a penetration rate for each of the four levers studied and direct reduction impact for each of the 7 chemicals in scope. LC and HC scenarios are possible by using different assumptions on the penetration rates. Data series are available in the 'Demand Map' excel file, in the tab labelled 'R&S – Packaging and HH goods'. Example for ethylene is shown in Fig S5.

- **Transportation:** *(for ethylene, propylene, methanol, benzene, toluene, xylene and butadiene)* Two levers, respectively extended vehicle lifetime, mobility-as-a-service, are considered (24). Previous studies establish that substitution in the transportation industry is not a relevant lever (25). Data is provided by the authors of XaaS report which estimate that mobility-as-a-service could reduce up to 33% of car production and lifetime extension of 21% by 2050. Penetration rates are used from the same source resulting in a non-linear growth as followed: 10% by 2030, 40% by 2030 and 100% by 2050. Each lever is assumed to have an equivalent transversal across all chemicals. The modelling results in a penetration rate for each of the four levers studied and direct reduction impact for each of the 7 chemicals in scope. Those numbers result in a HC scenario; a LC scenario is possible by assuming only 50% penetration rates at any given year. Data

series are available in the 'Demand Map' excel file, in the tab labelled 'R&S – Transportation'. Example for ethylene is shown in Fig S6.

- **Buildings and Construction:** *(for ethylene, propylene, methanol, benzene, toluene, xylene and butadiene)* Two levers, respectively reduction of m^2 usage per capita, and material substitution, are considered (26). Data for reduction levers are used as proposed by the authors from ODI and triangulated with suggested recommendations from the International Resource Panel (27). Data for substitution levers are adjusted to account for GHG emissions (e.g., no plastic to metal substitution which are unfavorable (13)) of product substitution and penetration rates. As a result, it is assumed that only 50% of profiles could be substituted, pipes would not be able to be substituted and substitution in other categories would be limited to 90%. Penetration rates are assumed to be linear given substitutes already exist at commercial scale. Each lever is assumed to have an equivalent transversal across all chemicals. The modelling results in a penetration rate for each of the two levers studied and direct reduction impact for each of the 7 chemicals in scope. Those numbers result in a HC scenario; a LC scenario is possible by assuming only 50% penetration rates at any given year. Data series are available in the 'Demand Map' excel file, in the tab labelled 'R&S – Buildings'. Example for ethylene is in Fig S7.

- **Apparel:** *(for ethylene, methanol, toluene, xylene and butadiene)* Levers in the textile sector are applied only to apparel as no data is available for the rest of the textile industry. Four levers, respectively waste reduction, reuse and new business models, reduction in consumption and substitution to alternative materials, are considered (28). Data for reduction levers are used as proposed by the authors from McKinsey and extrapolated to 2050 where it is estimated that waste reduction in the value chain can eliminate needs for 25% of materials, and new business models for up to 34%. Data for substitution is calculated from average publicly available CAGR (~5%) from the 'man made cellulosic fibers' sector (which include lyocell, viscose, etc.) and assume to substitute PET fibers only. It leads to a 20% substitution potential by 2050. Penetration rates are assumed as followed: for waste reduction 70% by 2030, 90% by 2040 and 100% by 2050; new business models: 20% by 2030, 50% by 2040 and 100% by 2050, then substitutes already exist at commercial scale and penetration rates are assumed linear. Each lever is assumed to have an equivalent transversal across all chemicals relevant for the sector. The modelling results in a penetration rate for each of the two levers studied and direct reduction impact for each of the 5 chemicals in scope. Those numbers result in a HC scenario; a LC scenario is possible by assuming only 50% penetration rates at any given year. Data series are available in the 'Demand Map' excel file, in the tab labelled 'R&S – Apparel'. Example for ethylene is in Fig S8.

- **Other sectors:** *(for ethylene, propylene, methanol, benzene, toluene, xylene and butadiene)* Blanket innovation levers in other sectors are applied, respectively reduction and substitution to reflect that all sectors would likely be impacted if radical changes are to be seen in the sector studied. Assumptions are made based on the smaller reduction and substitution potential observed in other sectors: reduction of 15% and substitution rate of 5% by 2050. Penetration rates are assumed to be linear between 2020 and 2050. The modelling results in a penetration rate for each of the two levers studied and direct reduction impact for each of the 7 chemicals in scope. Those numbers result in a HC scenario; a LC scenario is possible by assuming only 50% penetration rates at any given year. Data series are available in the 'Demand Map' excel file, in the tab labelled 'R&S – Others'. Example for ethylene in Fig S9.

Waste Generation

A waste model is created to estimate contribution of recycling as well as calculate scope 3 emissions arising from end-of-life. The waste model created only focuses on plastics given the lack of data regarding the fate of other chemicals.

Waste generation is obtained for each industry (15) and at the polymer level(29) for 2015 and extrapolated to 2020 using historical growth (13). Waste generation data from automotive and building and construction is noted to be lower than expected but no global dataset as comprehensive is found. Waste projections to 2050 are made using year-by-year weighted average growth rates of the two major chemical contributors: ethylene and propylene from IEA previously used for consistency.(3) It results in an overall 1.2% CAGR for plastic waste generation between 2020 and 2050.

The chemical-derived share of each industry waste is then calculated based on previously established matrix of chemical usage per industry for 2020. The share of chemical usage per industry is assumed to be constant between 2020 and 2050. The ratio of waste generation vs production for each chemical is then calculated and found to be appropriate based on literature (15) ranging between 80% and 90%. The resulting waste baseline is then discounted to account for circular business model levers (e.g., reduce and substitute) based on previous calculations. Discount rate is calculated as the ratio of post reduce and substitute demand divided by BAU demand for any given year for each individual chemical. Discount rates from reduce and substitute only apply after a set timeline given the existing average lifetime of goods in each industry. Delay between production and waste generation are used according to existing literature (15): packaging: 1 year (no delay); transportation: 12 years; buildings and construction: 30 year; textiles: 5 years; others: 5 years.

Data series are available in the 'Demand Map' excel file, in the tabs labelled '[Chemical name] Recycling LC' or '[Chemical name] Recycling HC' – row 1 to 50. Example for ethylene is in Fig S10.

Mechanical Recycling

Once the chemical-derived plastic waste generation is calculated, mechanical recycling contribution is calculated. Recycling rates for 2020 are obtained from existing literature (13, 29, 30). Recycling rates for 2050 are obtained from different sources:

- **Packaging and household goods:** extrapolation from 2040 to 2050 based on Breaking the Plastic Wave analysis (13).
- **Transportation:** target objective of the industry obtained from Ellen MacArthur Foundation via ODI (25).
- **Apparel sector:** extrapolation from Ellen MacArthur foundation, A new Textile Economy report (30).
- **Buildings and Construction:** Assumed to be twice more than existing recycling rates in Europe today and aligned with industry objectives (31).
- **Others:** blanket assumption of 20% - considerably lower than other sectors given the complexity of the remaining materials (e.g., low volumes, highly technical plastics).

Figures derived from the above literature provide assumptions for the HC scenario. In the LC scenario, recycling growth and therefore recycling output volumes are assumed to be the same. Recycling rates in the LC scenario are lower because more plastic waste is generated in this scenario.

Data series are available in the 'Demand Map' excel file, in the tabs labelled '[Chemical name] Recycling LC' or '[Chemical name] Recycling HC' – row 50 onwards. Example for ethylene and propylene is in Fig S11.

Recycling outputs are then calculated by industry and mapped to analyze in which sector they would be most likely to be used and therefore to abate further chemical demand. Recycling rates are dividing into two types of recycling:

- 'closed loop' defined in this analysis as recycling outputs are used by the same industry and,
- 'open loop' defined in this analysis as recycling outputs are used by a different industry recycling

Assumptions for each industry can be found below:

- **Packaging and household goods:**
  - Data regarding 'closed loop' (defined in this analysis as recycling outputs are used by the same industry) and 'open loop' (defined in this analysis as recycling

outputs are used by a different industry) recycling is obtained and used between 2020 and 2040 then extrapolated to 2050 (13).
    - Recycling outputs fate data for 2020 is obtained from the open-source database published by Closed Loop Partners for Canada and the United States which is used to estimate in which industry packaging waste recycled as 'open loop' would flow. Data is extrapolated to the global dataset and assumed constant overtime (except for textile where data is adjusted given very high reduction and substitution rate).
- **Transportation:** data is obtained from WRAP for 2020(32) and estimated based on existing industry literature for 2050.(31)
- **Apparel sector:** although no firm dataset is found, data is estimated based reports descriptions from Ellen MacArthur foundation, a new Textile Economy report and WRAP (30, 32).
- **Buildings and Construction:** Assumed no open loop recycling occurs.
- **Others:** Assumed no open loop recycling occurs.

Data series are available in the 'Demand Map' excel file, in the tabs labelled '[Chemical name] Recycling Rates'. Example for ethylene is in Fig S12 and Table S2-3.

<u>Waste management</u>

Plastic waste management assumptions are made based on municipal solid fate globally obtained for 2040 and extrapolated to 2050 from data provided by the authors of Breaking the Plastic Wave (13). Only two waste management scenarios have been implemented in this model: one for BDEM and one for HC and LC. Assumptions can be found below. Waste management assumptions are used to calculate scope 3 emissions from end-of-life.

In LC and HC scenarios, it is assumed that by 2050, the use of unabated incinerator would not be allowed in a net zero economy and therefore, all existing incinerators would be provided with CCS units. Ramp up of CCS units on incinerator follows the same S-curve used for chemical recycling (see next section).

Data series are available in the 'Demand Map' excel file, in the tabs labelled '[Chemical name] Recycling LC' and '[Chemical name] Recycling HC'. A summary is shown in Table S4.

<u>Upstream chemical Recycling</u>

Upstream chemical recycling technologies which have a TRL>6 are considered in this study including (1) depolymerization of PET, (2) solvent based recycling technologies also referred to as dissolution technologies. Each of those two technologies are considered in the demand model because their output(s) remove the need for the basic chemicals in scope for this report (i.e., ethylene, propylene, methanol, benzene, toluene, xylene, butadiene). Other thermochemical recycling technologies such as gasification and pyrolysis are producing respectively syngas and pyrolysis oil and therefore lead to the production of the basic chemicals in scope for this report. As such, pyrolysis and gasification are considered as part of the supply model.

Growth of chemical recycling technologies is estimated and capped by feedstock availability. Only plastic waste is accounted in this cap which has been collected and sent through a sorting center (or material recovery facility) which is estimated based on Breaking the Plastic Wave data.(13)

- **PET depolymerization:** remaining PET waste is calculated post recycling and used as a cap. (*29*)
- **Solvent-based recycling:** 'high quality' remaining plastic waste is estimated post recycling and used as a cap to account for the fact that this technology requires plastic waste grades with relatively high level of purity (33). It is set at 50% of available remaining plastic waste and is assumed to be relevant for any polymer type.

An S-curve is then applied to estimate growth of the technology given the set constraint. The S-curve used is initially designed for hydrogen production process ramp up but considered relevant in the context of scaling a process in the chemical industry (34).

$$x = \frac{a}{\left(1 + e^{(-b*(y-M))}\right)^{a}}$$

where *x* is the penetration rate of the technology, *y* is the year (varying from 0 to 30), *a* = 1.9, *b*=0.35, *c*=1 and *M*=15.

The S-curve is applied separately to waste available from high income where the technology is assumed to be available today (year 0 = 2020) and middle and low income where the technology is assumed to only be available in 2035 (year 0 = 2035).
Data series are available in the 'Demand Map' excel file, in the tabs labelled '[Chemical name] Recycling LC' and '[Chemical name] Recycling HC'.
Remaining plastic waste (i.e., post mechanical and upstream chemical recycling) that has been collected and sorted is assumed 'available' for pyrolysis in the supply model.
Remaining plastic waste (i.e., post mechanical and upstream chemical recycling) that is sent to landfill and incineration 'available' for gasification in the supply model.

Waste management cost

Investment cost is estimated based on literature data for waste management infrastructure. (13) The following CAPEX cost (Table S5) are applied to calculate required investment cost of each scenario and, assumed constant between 2020 and 2050 and applied to year-by-year waste management additional volumes capacity compared to 2020.
Data series are available in the 'Demand Map' excel file, in the tabs labelled 'Job + CAPEX analysis'.

Scope 3 end-of-life emission factors for carbon-based chemicals

Scope 3 emission factors for each chemical are calculated to access end-of-life scope 3 emissions as well as GHG emissions captured by CCS on incinerators. The following methodology is used:

- **Step 1:** Emission factors per end-of-life fate is obtained (Table S6) (13). In a BDEM scenario, emissions factors are assumed constant overtime. In LC and HC scenario, it is assumed other sectors (especially the energy sector) would reach net zero and therefore emission factors associated would decrease to 0.
- **Step 2:** Emission factors for incineration are estimated based on EPA Warm Model (v15) (35) and calculated for each chemical based on the polymer composition of the chemical-derived plastic waste generation (Table S7). Toluene is modelled using 'mixed plastics' given no value could be found for polyurethanes. Butadiene is modelled as ethylene given no specific value could be found for synthetic rubbers. In the HC and LC scenarios, residual emissions from CCS are calculated using a 95% capture rate consistent with assumptions in the supply model. In the BDEM scenario, incineration without CCS is assumed to be equivalent to full combustion given the use of zero emission energy in the rest of the economy. At this point, the energy recovery component of incinerators cannot claim offsetting compared to average grid emissions.
- **Step 3:** Year-by-year weighted average emission factors are calculated for each chemical based on the emission factors above and the fate of the chemical-derived plastic waste fate at end-of-life.

Data series are available in the 'Demand Map' excel file, in the tab labelled 'Scope 3 Yearly'.

Scope 3 end-of-life emission factors for ammonia

Scope 3 emission factor for ammonia is calculated to access end-of-life scope 3 emissions. The following methodology is used:

- **Step 1:** Emission factors per end-of-life fate is obtained for 2020 (22, 36). Based on the latest IPCC report, NOx emissions are excluded as GHG. Therefore, only emissions factors associated with the formation of N_2O through fertilizers are found relevant for this work. Other fertilizers are estimated as an average of urea and ammonium nitrate values for modeling purposes given other fertilizers demand is not modelled in detail.
- **Step 2:** For BDEM scenario, emission factors are assumed to be constant. For LC and HC scenarios, it is estimated that 25% N_2O emission reduction is possible according to the IPCC latest report if better land practices are deployed together with N_2O inhibitors (Table S8) (36).
- **Step 3:** Year-by-year weighted average emission factor is calculated based on the emission factors above and the fate of ammonia at end-of-life.

Data series are available in the 'Ammonia Demand Map' excel file, in the tab labelled 'Emission Factor Scope 3'.

Supply Analysis
<u>Aim and Structure</u>
The supply model analyses how the demand for the eight primary chemicals can be met while reducing the emissions from production & feedstock lifecycle stage towards net zero. At the heart of the model is a calculation of the economic and environmental costs and footprints of the chemical manufacturing sector described in the supply model system map (Section). The analysis subsequently calculates the supply technology mix to meet the annual demand in the period 2020-2050 under various levers of interest.
It should be noted that the model is not a dynamic optimization model to reach precisely net zero, but it is based on a bottom-up plant level decision making algorithm that drives the overall emissions reduction in the abatement scenarios. The model forces year-by-year changes to the plant fleet[2] to meet the global demand for the given chemicals. Input assumptions (e.g., techno-economics, emissions, etc.) and system boundaries are put in place to enable a year-by-year optimization of which plants to build, retrofit or retire. The agent-based model is therefore fundamentally different from commonly used TIMES algorithms and linear optimization (3, 5). Linear optimization models are usually designed to identify optimal strategies based on the given constraints. With a single set of strategies, these approaches generate single best outcomes (37). This method is adequate if the chosen optimum strategy is relatively robust to these key assumptions. However, if the optimal strategy is sensitive to those assumptions, linear optimization loses their prescriptive value (38). Finding robust strategies requires evaluation of different lever options over a large ensemble of possible future states of the system. To answer the "what-if" question and to generate a large number of scenarios, a simulation model is mandatory. The agent-based supply side model allows us to actually to evaluate the "what-if" questions instead of pretending to be able to present an optimal strategy in a deeply uncertain future. The model outcomes are therefore not a predetermination, projection or forecast of any kind, but rather a set of views on key elements of the industry's direction of travel across the next 30 years if it is to reach net zero. Insights that are possible from this include how much this transition will cost, how much emissions can be abated, and the technological and geographical shifts that could occur in global chemical production of these primary chemicals. The model and our analysis do not specify the nature of system interventions that enable these external constraints to incentivize this transition to net zero by 2050. Through this approach the model provides us with a hypothetical carbon pricing that will be required to achieve the emission reduction over time in each scenario. The interventions could however similarly include mandates and other regulatory measures.
Several external constraints are applied to enable the transition towards net zero by 2050:

- Abated production technologies are preferred over non-abated technologies for new-build technologies.
- A fixed retrofit rate of 5% is applied. This rate is adjustable in the model and assumption-based, but a much lower rate would drastically increase the cumulative CO_2 emissions of the sector.
- Non-abated production sites are decommissioned in the model starting from 2035.

As described in the main manuscript, different combinations can be taken on the supply scenarios, that, when combined with the three possible demand scenarios, give rise to a matrix of possible demand-supply pathways to study. The different supply angles are designed to each capture a different set of priorities and therefore to offer a view on sensitivity depending on which parameters are optimized for. These sets of priorities are used to rank the various possible technologies for new-build, retrofit and decommission in each region combination per year (Fig. S14.). This results in a ranking list for each chemical, which is then used to build a scenario for that chemical in a stacked fashion (see next section). In addition to the pathways described in the main manuscript, there is also a 5th supply scenario called fastest abatement which uses identical ranking logic as the "No new fossil by 2030" but does not penalize against fossil feedstock. Its

[2] The modelled plant fleet is not directly correspondent to existing assets with their specific location and specific production parameters but assumed as an average plant in a given region.

outcome lies in the middle of the “Most Economic” and “No new fossil by 2030” and is therefore not included in the main analysis.

Additional pathway features:

- While the BAU pathway allows for initial technologies to be built all the way through to 2050, all other pathways only allow this up to the year 2025 – in order to capture existing commitments and plants already under construction – after which initial technologies are forced to the bottom of the ranking list.
- If a transition technology is available in the same year as an end-state technology in the same region, it will be deprioritized in favor of the end-state technology. The model’s underlying assumption is that the drive to net zero in 2050 cannot wait if better technologies (i.e., technologies offer more GHG emissions abatement) are available at comparable cost, and that plant owners will prefer not to implement two separate investment rounds before 2050 (one from initial transition and the second from transition end-state)Model Map

The model follows a stepwise approach: (Fig. S13).

1. Data import from “Master template” file including information on
    a. Technology information (Section 0): Techno-economics, feasible retrofit pathways, availability timelines, multi-product ratios and embodied emissions
    b. Emissions Factors (Section 0)
    c. Prices (Section 0)
    d. Availabilities (Section 0)
    e. Decommission rates (Section 0)
    f. Production capacity in 2020 by technology and region (Section 0)
2. Calculation of key variables based on input (e.g., levelized cost and emissions of each technology across time and region), see Section 0.
3. Ranking of technologies for new-build, retrofit and decommission in each year and calculation of plant stack in each year for each chemical over time (see Section 0)
4. Analysis, export of outputs and sensitivity analysis (See Section 0)

The following three categories of data are key data inputs for the model:

- 2020-2050 demand for all chemicals (BAU, High Demand and Low Demand) derived from the demand model discussed in Section 0. The information can be found in “Master template” under the “Value – Chemical X” for each technology.
- 2020 global production capacity of all chemicals in scope, split into technology type and geographic region. The information can be found in “Master template” under the “Value – Chemical X” for each technology.
- Technology options per geographic region per year – ranked according to several different sets of priorities by scenario.

The simulation is approached year-on-year, where, for any given chemical, the global demand for each year must be met by the sum of all regional production in that same year (Fig. S16.).

For a given year *Y*, the following actions are taken:

A. The chemical *X* may be a by-product from another chemical (e.g., propylene as by-product from steam cracking).

B. 5% of initial plants are retrofitted: a plant is converted from one technology type to another on the same site (e.g., Steam methane reforming + Haber Bosch to Steam Methane Reforming + CCS + Haber Bosch). Alternatively, the plant may also be decommissioned and replaced with a new-build plant (at a potentially different location) which is also considered to be a retrofit action.

C. If year *Y*>2035, unabated initial technology plants that cannot be retrofitted are decommissioned with a rate given in “Master Template” in sheet “Decommission”.

D. If the demand is not yet met, new-build plants are added based on the new-build ranking.

E. If production exceeds demand, existing plants are decommissioned based on the decommission ranking (more information in Section 0). Plants that are added during the period of the model run (2020-2050) are only decommissioned at the earliest of

20 years after construction, as it is assumed that these would be too new otherwise to shut down and would rather lower their capacity utilization factor.

B and D are directly compared according to the ranking variables (Fig. S14. and Section 0) above and the highest ranked option is chosen. This option is implemented as many times as possible until demand is met. If any constraint is met (see Table S2.), the next option on the ranking is implemented in order to avoid violating any constraints. This continues until it also hits a constraint and moves onto the next option on the ranking list, and so on.

Once the technology switch rate is reached but there is still a supply deficiency compared to demand in that year, D is triggered to fulfil the remaining demand. This is done while still respecting all other constraints (e.g., technology ramp up rate of novel technologies; resource availability, etc.). This exercise is then repeated for each year with data from the last year functioning as baseline.

Additional model features:

- Methanol is both input and output of the supply model. See 'Inter-Chemical Interactions', Section 0.
- Multi-product processes are assigned 'primary' chemicals and 'secondary' chemicals. For example, ethylene is the lead chemical for all steam cracking based processes and is the only chemical allowed to build, retrofit or decommission steam cracker. See Section 0.
- From year 2040 onwards, any plants of transition technologies (defined in Section 0) remaining are forced to undergo a 'second retrofit' to an end-state technology by the year 2050.

Technology information

Technologies and input parameters

This section will describe how technology archetypes are selected and the parameters of each that are considered in this model.

For every technology business case, the following data is gathered (see "Master Template" under "Values – Chemical X"):

- Plant capacity[3] (ton chemical/day)
- Plant lifetime[4] (years)
- Plant capacity factor (%)
- Material input requirements, including feedstocks both as reactants in chemical synthesis as well as for fuel (GJ/ or ton/ton chemical product)
- Power requirements (GJ/ton chemical product)
- Capital expenditure (USD/ton chemical product per annum)
- Fixed operations and maintenance costs are assumed as 5% of CAPEX unless expected to differ widely from this (USD/ton chemical product per annum)
- CO_2 capture rate, where CCS is deployed (%)

All CAPEX costs are assumed to be constant over time unless our research revealed projected changes as a result of technology maturation and associated improvements in both technology efficiency and economies of scale. This is most relevant for end-state technologies where there remains potential for maturation. The most important example of this is electrolysis towards green hydrogen production based on falling costs for electrolyzers and renewable energy. Note that the electrolyzer costs forecast shown in Fig. S17 are taken as an average of proton exchange membrane electrolyzers and alkaline electrolyzers, as our work does not aim to propose one type of electrolyzer over the other(39). Hydrogen prices for processes where hydrogen is used as feedstock are shown in Fig. S18.

Technology timelines

For each technology, a start date is defined, from which the model thereafter allows the technology to be built (Fig. S19). Note that there are no corresponding end dates for any

[3] To enable a faster runtime of the model, a parameter called "plant_spec_override" is active in the config file, putting each plant at 3000 t/d and therefore limiting the overall number of plants handled in the model.

[4] This metric was not actively used in the model. An economic lifetime for net present value calculations of 25 years was assumed. Physical plant lifetime is typically not a limiting factor for chemical plants as they can run for >50 years if well maintained.

technology other than a forced forbidding of initial technology deployment after the year 2025 in all net zero scenarios.

Technology selection and categorization

For each chemical, technology archetypes that can contribute towards partial or full GHG emissions reduction of its production are considered for inclusion in this model. Each is bucketed under one of three categories:

- Initial: technologies used today to produce chemicals from fossil fuel feedstocks and energy sources; incompatible with net zero world.
- Transition: technologies that partially reduce emissions of Initial technologies; generally considered to be available today or sooner than End-State technologies, therefore paving the way prior to the latter becoming available at scale.
- End-State: technologies where emissions are abated by CCUS and/or by clean carbon-free energy and/or feedstock sources; compatible with net zero world.

All included transition and end-state technologies are considered to have a TRL >6 today. It is considered beyond the remit of this work to analyze lower TRL technologies quantitatively due to uncertainty with regards to their scalability and economic parameters.

Specific technology abatement calculations

Not all information for certain abated technologies is publicly available. Sources and notes on assumptions taken for any of the input data are listed in detail in the "Master template.xlsx" file. Four specific calculations should be highlighted here in particular:

1) BTX production (catalytic reforming and MTA)

A large share of BTX[5] is produced via catalytic reforming. Different variants of it exist today in industry achieving different production shares of B/T/X, depending on blending of aromatics into the gasoline pool. Four cases are modelled as possible extremes across the spectrum. The business case information is obtained and derived from "Benzene Production from Naphtha – 2021 Q1 report", a product of Intratec (www.intratec.us) (40). The respective production share of these four business cases is determined by subtracting the production of aromatics from steam cracker business cases of global demand. The production economics are typically averaged across these business cases.

a. "Refineries Benzene/Xylene + TDP": Extraction of Benzene, Toluene and Xylene from the reformate. All Toluene is converted to Benzene and Xylene via Toluene disproportionation (TDP).
b. "Refineries Benzene no TX extraction": Only Benzene is extracted from the reformate.
c. "Refineries Benzene/Toluene/Xylene + TX extraction": B/T/X are all extracted from the reformate.
d. "Refineries Benzene/Xylene + no toluene recovery": The Toluene is all blended into the gasoline pool, but Benzene and Xylene are extracted.

Similarly, MTA has business cases with and without TDP.

2) Butadiene production from bioethanol

The business case information is obtained and derived from "Butadiene Production from Ethanol – 2021 Q1 report", a product of Intratec (www.intratec.us) (41).

3) Co-feed hydrogen for methanol production

Methanol production emissions from gasification can be abated by adding green hydrogen into the process gas mix to adjust the ratio of hydrogen, carbon monoxide and carbon dioxide to yield a stochiometric number of 2.05 (42). Thereby no CO_2 in the process gas needs to be separated and removed. Process gas data from two sources are used for coal gasification (43) and biomass (44) respectively. The MSW gasification business case is modelled based on the same assumptions as the biomass gasification case. It is assumed that the gasifier size would remain unchanged but a higher capacity for the methanol synthesis unit could be achieved. Similarly, 10% of CAPEX is subtracted compared to the base-case due to cost savings in water gas shift and CO_2 removal.

---

[5] Xylene in the context of this manuscript always refers to para-xylene (the highest demanded isomer). Xylene production technologies therefore include the CAPEX for p-xylene separation.

4) CCS cost for gasification plants

CCS energy consumption and CAPEX for business cases where no literature values could be found are estimated based on IEA reports on SMR+CCS (45) and coal power plant CO_2 capture (46).

Calculations

From the above input parameters, key intermediate parameters are calculated for each technology in each region for each year, using the equations listed in Table S10. as they pertain to the relevant step in the model.

Emission Factors

Emission factors are categorized according to scope 1, 2 and upstream 3 emissions representing the lifecycles of production (scope 1), energy (e.g., electricity) during production (scope 2) and feedstocks (scope 3 upstream). The emission factors can be found in Master template in the "Emissions" tab.

Note that scope 3 end-of-life emissions are calculated outside of the supply model as they depend on waste management and are therefore closely linked to the demand model (Section 0-0).

In this model, three possible sources of energy are assumed, each of which has their associated emission factor and price.

1. Grid: electricity from the grid with associated emissions factor.
2. Grid – PPA: grid power under a renewable power purchase agreement. All transition and end-state technologies rely on Grid – PPA to avoid electricity associated emissions.
3. Direct RES: On-site renewable energy generation for on-site hydrogen generation. As shown in (39), hydrogen production from dedicated renewables is the lowest cost production pathway.

The emission factor (scope 2) for grid electricity is shown in Fig. S20. for both the BAU scenario (no climate targets aligned decarbonization of energy system) and in the abatement scenarios (aligned to 1.5°C pathway). Note that electricity taken from the grid with power purchase agreements and electricity generated on-site from RES are assumed to have an emission factor of zero.

Emissions for fugitive methane emissions are assumed to remain constant for the BAU scenario while a 20% and 50% reduction is assumed in the abatement scenarios compared to 2020 values. These assumptions are based on slightly more conservative views compared to the global methane pledge which aims to reduce emissions by 30% by 2030 (combined lower use and lower emissions factors) and the IEA NZE which suggests a drop of 75% needed for 1.5°C until 2030 (of which 50% emissions factor reduction, 25% lower use of fossil) (47).

Point source CO_2 is assumed to have no associated emission factor based on the assumption that in a decarbonizing world, increasing shares of bio feedstock will displace fossil carbon feedstock in adjacent sectors (e.g., steel, cement, incinerators).

Emission Share

The emissions of a given process are calculated based on the input feedstocks (e.g., X t of natural gas/ t methanol). The model takes into account that some of the input carbon ends up in the carbon-based products and does not turn into CO_2 emissions during production. The data table with this information is called "Emissions share" within the "Master template.xlsx" file.

A few important considerations:

- Emissions from steam crackers are handled via a raw-material output called "off-gases". Naphtha has no associated scope 1 emission factor to avoid double counting and the emissions share is therefore 0 for all naphtha-based processes.
- All carbon from energy use is turning into CO_2.
- Biomass-related CO_2 emissions are considered CO_2 neutral.
- CO_2 capture is applied to all CO_2 emissions: raw material and fuel use.

Prices

Material Inputs

Projected material prices are either taken directly or derived from cited sources and can be found in Master template in the sheet “Prices and Availability”. Derivations are necessary in the case where feedstock prices are either not available in the public domain or notoriously difficult to predict. The rationale behind some of the key derivations are as follows:

- Naphtha prices (Fig. S21.
-) are assumed to follow crude oil prices, scaled with a constant factor of derived from IEA Future of Petrochemicals report (3). The high-cost scenario is generally based on the relative slope of the RTS scenario with strongly rising crude oil prices and the low-cost sensitivity is based on the CTS scenario with sinking/flat fossil fuel prices. 2020 Baseline values are taken as average of 2015-2020 cost points derived from historical data.
- Ethane and Propane prices are assumed to follow naphtha prices, scaled with a constant factor derived from (3). The only exception is for North America, where its access to abundant shale-gas derived propane results in a propane market that is unique in the world. In this case, propane prices are assumed to follow natural gas prices via a linear relationship that is deduced from historical data.

$$Propane\ price\ \left(\frac{\$}{MMBtu}\right) = 3.15 * Natural\ gas\ price\ \left(\frac{\$}{MMBtu}\right) - 0.8$$

- Point source CO_2 is priced at 0 \$/t, since it is very difficult to forecast what the price of CO_2 will be.

Natural gas and coal input prices are shown in Fig. S22 and Fig. S23.
Note that in the case where either a ‘high’ and a ‘low’ option are available for the price of a particular feedstock, the ‘high’ price is taken as the default and the ‘low’ is utilized for sensitivity analyses.

Power & Hydrogen

Power price assumptions for the three types of power are given in Fig. S24. Direct RES is a special case within the model. It is exclusively used to provide power for dedicated on-site hydrogen production for technology business cases that exclusively deploy green hydrogen as feedstock (i.e., not for co-feed cases). The cost for direct RES in Fig. S24. Is shown as levelized cost, but within the model the business case is handled differently; it is assumed that the CAPEX for the renewables is spent as part of the investment into a new plant (e.g., Electrolysis for green hydrogen plus Haber Bosch for green ammonia production). The OPEX for the direct RES is therefore only the fixed O&M contribution of direct RES.

CCS Capture

CCS capture costs are calculated as part of the business case via an additional CAPEX (for CO_2 capture facility) and OPEX (i.e., additional natural gas and/or electricity energy demand for absorption/desorption of CO_2). The cost is therefore captured as part of the intrinsic economics of the abated chemical production. A CO_2 capture rate of 90-95% is assumed depending on process. This is in line with theoretically feasible capture rates[6] and is perceived as minimum to be considered as “abated”.
CCS Capture costs for end-of-use incinerators is calculated at \$67/ton CO_2 capture cost. This is derived based on the assumption of the cost of coal power plants and downscaling of the plant size.

CCS Transport and Storage

The costs of transporting and storing captured CO_2 are based on the recent seminal modelling work of Smith et al.(48), where regional variations in such costs are estimated. Classification of the regions in our model according to their tiers are given in Table S11..

<u>Availabilities</u>

The availabilities are listed in the “Prices and Availability” tab in “Master template.xlsx”.

Material Inputs

The availability of various feedstocks is capped according to the following reasons (where possible, availability at a regional rather than global level is taken, as indicated):

[6] Currently demonstrated capture rates are often lower (e.g., 60%) because only process CO_2 is captured and not CO_2 in the flue gas created for heat & power production. This is significantly lower cost due to the higher CO_2 concentration in the process stream.

Global caps

- Biomass: the total global biomass availability for the chemical sector is based on iterations on work done by the Energy Transition Commission (10), where biomass allocation guidelines are utilized to allocate biomass to industrial sectors that could require biomass in a net zero world.
- Bio-oils (i.e., waste from cooking oil): based on work done by the Energy Transition Commission (10) that assumes 1.5 EJ/year of waste bio-oil available today. A conversion factor of 40 GJ/t bio-oils is used.

Regional caps

- Biomass: The overall biomass availability is then divided into the regional availability by respective land-area.
- Pyrolysis oil: derived purely from regional availability of recycled waste plastic, which is calculated by the demand model above (see Demand Analysis section 0). A conversion factor of 0.8 tons of pyrolysis oil for every ton of pyrolyzed waste polymer is assumed.
- Municipal solid waste RDF and treated wastewater: derived from municipal solid waste RDF and treated wastewater per capita today, respectively, and extrapolated between 2020-2050 based on projected regional urban populations (effective waste management not considered for rural populations).

CO_2 Storage

The regional availability of CO_2 storage volumes for CCS is based on the ramp up of infrastructure developments to enable use of the theoretical regional storage. Currently only about 40 Mt CCS storage capacity are available worldwide (49). A model is developed using data from the Global CCS Institute (49) to establish a regional CO_2 storage capacity that increases over time to yield 7 Gt of CO_2 storage by 2050, of which 25% are allocated to the chemical industry (Fig. S25.). This is directly relevant for all technology cases where CCS is an emissions-reduction lever as it is particularly limited in the first 10-15 years.

Note that these caps are not in any way ‘allocated’ to the chemical industry but should be considered as a reasonable share compared to the needs of other major industries (e.g., cement production, negative emissions technologies such as DACCS).

Inter-Chemical Distribution of Resources

A mechanism to distribute capped feedstocks and CO_2 storage is required in order to avoid a few chemicals utilizing all the available resources. The cap is therefore distributed across the chemicals based on their demand volume (in Mt chemicals) in 2050.

<u>Decommission</u>

The model differentiates between two different processes for decommissioning plants:

1. Demand lower than production capacity: The most expensive or emission-intensive technology is decommissioned according to the ranking logic described in Section 0 and Fig. S14.
2. In abatement scenarios (ME, NFAX, NFS, FA), decommissioning for initial technologies that are not or cannot be retrofitted: With increasing rate (shown in Tab “Decommission” in “Master template.xlsx”), these technologies are decommissioned. This forced decommissioning is particularly relevant for catalytic reforming which has no retrofit options. This form of decommissioning takes priority over point 1, i.e., plants are first removed and only then demand is evaluated vs. the updated production capacity.

In general, “old” plants are decommissioned preferentially to younger plants to avoid distortions of retiring recently constructed plants (see Table S12. for 2020 starting point). Plants are considered “old”, once they have reached their lifetime or above it. Note, that this does not mean that the plant can’t continue to operate for significantly longer if well maintained. It is just used as an indicator for preferential decommissioning in the model. Since the model is based on open-source data or desktop research data, the age of the plants is randomly distributed across the initial plant stack, i.e., the plant representation is not specified to a specific existing asset and its corresponding location (beyond the region classification). These values can be found in the “Value – Chemical X” tab in “Master template.xlsx” under category “Production” and name “Current-day production % old”.

<u>Demand in 2020 and share of production technologies</u>

In order to be able to model the production of chemicals for the period 2020-2050, a view must first be had on the distribution of global production today. This serves as the starting stack of plants from which the model then begins its optimization for years 2021 onwards.

A rough distribution of chemical production capacity according to region and the technologies used to produce them is given in Table S12. These values can be found in the "Value – Chemical X" tab in "Master template.xlsx" under category "Production".

Ranking

The ranking variable depends on the scenario (Fig. S14.) and if two technologies rank equally, the next ranking variable in line is considered to distinguish between the two options. For example, in the Most Economic pathway, if two regions have the same LCOX in year *Y* for a given technology, region A will be ranked higher than region B if the scope 1+2 emissions of this technology in this region in year *Y* is lower than that of region B. Note that LCOX refers to levelized cost of chemical X considering a new-build of this technology. Note the same metric is taken even if the technology is being considered as a retrofit. To avoid over-precision, binning is applied, in which the entire ranking variable and the corresponding ranking is aggregated into 300 sub-bins. Subsequently, the actual ranking comparison is done on a bin-level.

Inter-Chemical Interactions

There are production technologies that impact more than one chemical at a time. This can happen in one of two possible ways: multi-chemical production or production from methanol.

Multi-Chemical Production Routes

In the event where a plant is based on a technology that produces more than one of the eight primary chemicals, its construction/retrofit/decommission will directly impact all of the chemicals it produces.

In order to manage this, each multi-product technology is assigned a 'primary chemical', which is often the chemical that is produced in the greatest proportion in the entire product slate and will be the chemical that determines if and when any action is undertaken on this plant. The assigned primary high-value chemicals and co-produced secondary high-value chemicals for the various multi-product processes are indicated in the tab "Multi chemicals ratios" in "Master Template.xlsx".

All other secondary chemicals produced by the same plant will automatically bear the consequences of any action to the plant, according to the order of events exemplified by the following example:

1. Technology X is a multi-product process that produces chemical A, B and C to the amounts of 3000, 6000 and 1000 tons/day, respectively (i.e., in the ratio 3:6:1). Chemical B is therefore the assigned primary chemical.
2. In modelling a scenario for chemical B, two plants of technology X are built in the year 2030. This equates to an added production capacity of chemicals A, B and C of 6000, 12000 and 2000 tons/day, respectively.
3. In modelling the same scenario for chemicals, A and C, an allocation of two plants of technology X per chemical are automatically considered for the year 2030. These plants' contributions of 6000 and 2000 tons/day towards partially meeting the demand for chemicals A and C, respectively, are also considered.
4. Chemicals A and C are not able determine on their own whether these plants are decommissioned/retrofitted in the future unless chemical B's model triggers these events. The production of chemicals A and C via technology X is therefore tied closely with the production of chemical B via this technology. This is necessary to avoid diverging actions being carried out on the same plant by its various respective products.

The order with which chemicals are modelled in the code is shuffled each year in a pre-determined fashion to avoid that one chemical is more crucial than another. For example, for certain "Refineries" processes (i.e., catalytic reforming) Benzene is the primary chemical, and for another Xylene, but they are both producing both chemicals. It should not be the same chemical always decommissioning their process before the other chemical.

Note that the associated costs and emissions of these multi-product processes are assigned to each high-value chemical according to the proportion in which they are produced relative to one another in the product slate. In some cases, the production process will also produce low-value chemicals (e.g., fuel gas that is cycled back to fuel the process, coke, methane); neither parts of the cost nor emissions are assigned to these low-value chemicals. For medium-value chemicals (e.g., pyrolysis fuel oil) a 50% multiplication of the volume share is used to calculate the apportioning for cost and emissions. An example is given in Fig. S27 to illustrate how costs and emissions are apportioned for a naphtha steam cracker.

Multi-chemical production technologies have the limitation that they may not allow for production and demand of multiple chemicals to fit. For example, the two business cases for MTA, one including, and one excluding Toluene disproportionation do not enable to produce the exact ratio of B/T/X as required by the demand model. This will have one of two consequences:

1. Further research in MTA catalysis research will allow for greater tunability of the catalyst selectivity.
2. The demand will follow due to increased prices (based on demand>supply) of the chemicals upon implementation of new production technologies

Production from Methanol

There are three technologies that involve utilizing methanol as a feedstock and producing one of the seven other primary chemicals: methanol-to-olefins (MTO), methanol-to-propylene (MTP) and methanol-to-aromatics (MTA). In sum, these three processes are hereafter referred to as MTX processes.

As methanol itself is a primary chemical that is being modelled, its role as both output and input of the supply model must be managed. This is done in the following ways.

Volumes of methanol available for MTX processes reflect the recent trend:

1. The volume of methanol required for MTX processes in year *X* is added on top of methanol demand for non-MTX processes in year *X*, and in sum constitute the total methanol demand for year *X*.
2. The availability of methanol in year *X*+1 for MTX processes is assumed to be 1.*5* x that of the volume of methanol required for MTX processes in year *X*. This acts as a ceiling on the available methanol volume in the following year, while still allowing methanol capacity to grow in order to meet increasing MTX demands. The amount of methanol actually being used by MTX is solely depending on the decommissioning/retrofit/new-build of methanol consuming MTX technology in the year-by-year stack optimizations.

1. The proportion of black (no emissions reduction) vs. green (complete emissions reduction) methanol being produced in year *X* is taken to be the assumed proportion of black vs. green methanol available for MTX processes in year *X*+1[7].
2. The price of black and green methanol as a feedstock for MTX processes in year *X*+1 is assumed to be the levelized cost of production of black and green methanol, respectively, in year *X*, plus a fixed margin on top in the Python model (100 $/ton methanol).
3. The scope 1+2 emissions of producing black and green methanol (t CO_2/t methanol) in year *X* is taken to be the scope 3 upstream emission factor of black and green methanol as a feedstock, respectively, in year *X*+1.

To enable a consideration of year-by-year costs, the ranking is performed twice, incorporating the levelized cost and emissions from the methanol production into the cost calculation of its derivatives.

<u>Key assumptions</u>

In addition to the sensitivity analysis described below, the user can change input assumptions in the “config.py” file of the Python code. These include (not exhaustive):

---

[7] Green and black classification of supply technologies can be found in the config file of the Python supply model. All end-state technologies are considered green.

- "NO_FOSSIL_FROM_YEAR": Year for the NFAX model, after which no new fossil plants are allowed. Default: 2030.
- "CARBON_PRICE": Carbon price in the model (added via the "Price and Availability" tab in "Master Template" as price for "carbon"). Default: not activated/false.
- "RETROFIT_CAP": Forced retrofit rate for each chemical each year. Default: 0.05 (=5%).
- "INITIAL_TECH_ALLOWED_UNTIL_YEAR": the year until which initial tech are allowed. Default: 2025.
- "SECOND_RETROFIT_EARLIEST_YEAR": the year from which second retrofits are allowed. This is relevant for transition tech plants. The earliest year they will be transitioned to an end-state tech is this year. Default: 2040.
- "MINIMUM_AGE_DECOMMISSION": Minimum lifetime of a new-built end-state technology asset after which it can be decommissioned. Default: 20.
- "MAX_TECH_RAMP_RATE": Technology ramp rate. The maximum addition of plants each year can be a maximum of this factor times the number of installed plants. Default: 1.3.
- "MAX_PLANTS_RAMP_UP": In the early years of a new technology, the maximum number of plants that can be added is this number (default 4). In collaboration with the ramp rate, these two factors determine how fast technologies can scale
- "REGIONAL_CAP": The maximum share of chemicals that can be produced in a given region to avoid over-representation of one region given the lack of trade modelling. Default: 0.3 (=30%).
- "DISCOUNT_RATE": Discount rate used as WACC for levelized cost calculation. The WACC is assumed equal across all regions. Default: 0.09.
- "ECONOMIC_LIFETIME_YEARS": Economic lifetime for LCOX calculation. Default: 25.
- "METHANOL_AVAILABILITY_FACTOR ": This factor (default 1.5) describes how much more methanol would be available in a given year compared the last year for MTX. For example, if 100 Mt methanol is produced in year *Y* out of which 80 Mt are needed for methanol demand other than MTX, and 20 Mt are used for MTX. In the year (*Y+1*), 1.5*20 = 30 Mt would be available for the chemicals that use MTX technology.

<u>Export, data output and sensitivity analyses</u>

The output data stored in the /output folder. The folder structure is based on "Geography/Scenario/Sensitivity" and then split in three different sub-categories:

1) Ranking files for new-build, retrofit (containing decommission & new-build) and decommission
2) Intermediate files calculated based on the model inputs
3) Final files containing results from the model run based on the plant stack build-up

In addition, a folder called "Aggregate" is also created that collects input across all scenarios into summary files. The file called "*date*_dashboard_output.xlsx" is the data export summary that can be imported into the "Global Dashboard" for full data analysis. The model also exports charts illustrating the technology mix over time for each chemical such as shown in Fig. S28-Fig. S97 in the sub-folder "Wedges".

Sensitivity analyses offer the opportunity to derive insights on the nature and size of key swing factors that could shift the outcome of our model, as well as give clues as to where pain points lie in the system.

The supply model offers a broad spectrum to change input assumptions and mechanics of the model. Two sensitivity analyses are incorporated in the model by importing modified input assumptions:

- **Applying a more stringent ramp up on CO_2 storage availability.**
    - A ramp up curve on CO_2 storage availability is already implemented as a default in the model.
    - An even more stringent ramp up (i.e., slower, therefore less CO_2 storage availability) is performed as a key sensitivity, and may be pertinent in the event that any of the following occur:
        - Lack of transport and storage logistics
        - Engineering challenges in ensuring no leakage at storage site

        - Long lead-times and high project failure rates
        - Engineering challenges around retrofitting existing plants with CO_2 capture units
        - Oppositions from the local community
- **Lowering future fossil feedstock costs.**

Performing this sensitivity addresses two fundamentally different views on how fossil fuel prices will develop in the future – either they will increase or decrease.

    - Higher fossil prices are the default in our model, and may be supported by the following arguments:
        - Pressure on markets to shift towards a net zero world will lead to high cost of capital and falling investments in upstream oil and gas infrastructure (already partially seen today), leading to declining supply and increasing cost of fossil fuel resources.
        - Falling gasoline demand will lead to unprofitability for refineries and therefore declining naphtha supply and, again, increasing costs.
        - Less local crude oil refining may require naphtha to be shipped, therefore increasing costs.
        - Efforts to lower upstream emissions from fossil fuel extraction will increase production costs.
    - Lower fossil prices are performed as a sensitivity, and may be supported by the following arguments:
        - In a shrinking market, high-cost locations will close production of fossil fuel extraction, leaving behind lower cost regions to supply the market.

Limitations to model and suggestions for future improvements
In the process of completing this study, a variety of limitations of the methodology comes to light. Some can be addressed, while some remain unaddressed. The latter is often due to a combination of lack of open-source data and lack of established conceptual methodology by other researchers in this field upon whom we could rely on to tackle difficult modelling challenges. Here, we hope to share the lessons learned and the opportunities for improvement in any follow-up work.

Demand Model

Data availability: globally available datasets for chemical demand, chemically derived waste generated (some data for plastic exist but are of poor quality): a more comprehensive, reliable and transparent dataset would greatly improve this analysis and allow for more granularity (e.g., mapping of more industries, or more chemicals).

Impact of upstream resource efficiency business cases: Very few studies, let alone review articles, are studying in detail the impact of existing circular business model on resource consumption, GHG emissions, and cost. The lack of data availability leads to greater uncertainty upon global extrapolation.

Supply Model

Technology learning curves: while lowering CAPEX costs for green hydrogen electrolysers are factored into the modelling, the same treatment is not performed on other end-state, emerging technologies – the vast majority of which maintain a constant CAPEX over time. Applying, for example, an S-curve logic to factor in technology learning curves and market penetration would improve the supply model.

Inter-regional trade assumptions: a key reason why demand is modelled globally while production is modelled regionally is the lack of understanding in global chemicals trade and in particular how this could shift as the sector and the world at large transition to a net zero future. It is therefore non-trivial to make assumptions as to how regional demand could eventually be met by regional supply.

Plant-level data: the chemical sector is extremely opaque and plant-level data on most chemicals (location, age, annual capacity, economic and technological parameters) is currently not openly available in the public domain. The consequences of lacking this data are two-fold: (i) a forced retrofit rate of 5% has to be employed in order to make up for the lack of visibility on plant age and thereby the plant life cycle with regards to anticipated investment/maintenance, and (ii) site-specific modelling that takes into account proximity of chemical plants to feedstock resources and CO_2 storage sites is not possible, rendering geographic granularity of the model to remain at the sub-continental scale. It is noted that the carbon clarity in the global petrochemical supply chain (C-THRU) project[8] led by our co-authors in Cambridge UK is working on an open-access data modelling framework to compile and reconcile data from disparate sources on plant, national, regional and global levels. This will help deliver the world's most comprehensive, reliable and transparent data and account of current and future emissions for the global petrochemical sector.

[8] Carbon clarity in the global petrochemical supply chain. https://www.c-thru.org/

The impact of the cost of chemicals

The section explains in detail how the cost increases in basic chemicals, such as olefins and aromatics, are estimated to impact the production cost of end-user products under the input-output price model.

The input-output price model is commonly shown as follows (50)

$$\begin{pmatrix} p_1 \\ \vdots \\ p_n \end{pmatrix} = \begin{pmatrix} a_{1,1} & \cdots & a_{1,n} \\ \vdots & \ddots & \vdots \\ a_{n,1} & \cdots & a_{n,n} \end{pmatrix}^T \begin{pmatrix} p_1 \\ \vdots \\ p_n \end{pmatrix} + \begin{pmatrix} v_1 \\ \vdots \\ v_n \end{pmatrix}$$

, where p_i and v_i are the price and the value-added coefficient for the i^{th}sector respectively, $a_{i,j}$ is the input coefficient, and T is the transpose of a matrix.

In order to calculate the impact of the price change in the basic chemicals sector on the price of all other sectors, we make p_n an exogenous variable, where the n^{th}sector is the basic chemicals sector, while p_1 through p_{n-1} remain endogenous variables. Then, the above equation provides

$$\begin{pmatrix} p_1 \\ \vdots \\ p_{n-1} \end{pmatrix} = \begin{pmatrix} a_{1,1} & \cdots & a_{1,n-1} \\ \vdots & \ddots & \vdots \\ a_{n-1,1} & \cdots & a_{n-1,n-1} \end{pmatrix}^T \begin{pmatrix} p_1 \\ \vdots \\ p_{n-1} \end{pmatrix} + \begin{pmatrix} a_{n,1} \\ \vdots \\ a_{n,n-1} \end{pmatrix} p_n + \begin{pmatrix} v_1 \\ \vdots \\ v_{n-1} \end{pmatrix}$$

Solving the above equation for p_i $(i = 1, 2, \ldots n-1)$ provides

$$\begin{pmatrix} p_1 \\ \vdots \\ p_{n-1} \end{pmatrix} = \left[\begin{pmatrix} 1 - a_{1,1} & \cdots & -a_{1,n-1} \\ \vdots & \ddots & \vdots \\ -a_{n-1,1} & \cdots & 1 - a_{n-1,n-1} \end{pmatrix}^T \right]^{-1} \left[\begin{pmatrix} a_{n,1} \\ \vdots \\ a_{n,n-1} \end{pmatrix} p_n + \begin{pmatrix} v_1 \\ \vdots \\ v_{n-1} \end{pmatrix} \right]$$

If p_n changes by Δp_n, the above equation will be shown as follows, if we assume v_i $(i = 1, 2, \ldots n-1)$ does not change when p_n changes.

$$\begin{pmatrix} p_1 \\ \vdots \\ p_{n-1} \end{pmatrix} + \begin{pmatrix} \Delta p_1 \\ \vdots \\ \Delta p_{n-1} \end{pmatrix} = \left[\begin{pmatrix} 1 - a_{1,1} & \cdots & -a_{1,n-1} \\ \vdots & \ddots & \vdots \\ -a_{n-1,1} & \cdots & 1 - a_{n-1,n-1} \end{pmatrix}^T \right]^{-1} \left[\begin{pmatrix} a_{n,1} \\ \vdots \\ a_{n,n-1} \end{pmatrix} (p_n + \Delta p_n) + \begin{pmatrix} v_1 \\ \vdots \\ v_{n-1} \end{pmatrix} \right]$$

, where Δp_i $(i = 1, 2, \ldots n-1)$ is the price change in the i^{th}sector caused by Δp_n.

Thus, $$\begin{pmatrix} \Delta p_1 \\ \vdots \\ \Delta p_{n-1} \end{pmatrix} = \left[\begin{pmatrix} 1 - a_{1,1} & \cdots & -a_{1,n-1} \\ \vdots & \ddots & \vdots \\ -a_{n-1,1} & \cdots & 1 - a_{n-1,n-1} \end{pmatrix}^T \right]^{-1} \begin{pmatrix} a_{n,1} \\ \vdots \\ a_{n,n-1} \end{pmatrix} \Delta p_n$$

The above equation can further be transformed as follows (51)

$$\begin{pmatrix} \Delta p_1 \\ \vdots \\ \Delta p_{n-1} \end{pmatrix} = \left[\begin{pmatrix} 1 - a_{1,1} & \cdots & -a_{1,n-1} \\ \vdots & \ddots & \vdots \\ -a_{n-1,1} & \cdots & 1 - a_{n-1,n-1} \end{pmatrix}^{-1} \right]^T \begin{pmatrix} a_{n,1} \\ \vdots \\ a_{n,n-1} \end{pmatrix} \Delta p_n = \begin{pmatrix} b_{n,1}/b_{n,n} \\ \vdots \\ b_{n,n-1}/b_{n,n} \end{pmatrix} \Delta p_n$$

, where $b_{i,j}$ is the Leontief inverse matrix of $a_{i,j}$. Leontief inverse matrix for the US and Japan is provided in (52) and (53) respectively.

For example, in case of Japan, if we assume the 1st sector is "foods", 2nd sector is "passenger motor cars, and nth sector is the basic chemicals sector, Japan's 2015 Leontief inverse matrix for 107 sectors provides as follows *(50)*:

$b_{n,1}$ = 0.007267 (for foods; sector #111)

$b_{n,2}$ = 0.012133 (for passenger motor cars; sector #351)

$b_{n,n}$ = 1.226802 (for basic chemicals; sector #203)

Thus, $b_{n,1}$ / $b_{n,n}$ = 0.007267 / 1.226802 = 0.6% and $b_{n,2}$ / $b_{n,n}$ = 0.012133 / 1.226802 = 1.0%.

Japan's basic chemicals sector (sector #203) includes ethylene, propylene, miscellaneous petrochemical basic products, pure benzene, pure toluene, xylene, and miscellaneous petrochemical aromatic products, under the sector name of "petrochemical basic products".

Key assumptions in this input-output price model analysis are as follows:

1. The results are based on the industry structure in 2012 for the US and in 2015 for Japan.

2. If an upstream industry (Industry X) increases the price, and each of the downstream industry accepts it and reflects it in its price to its downstream industries without changing the production volume and materials. Thus, Industry X's price increase propagates toward end users, while industries in between neither absorb nor inflate the price increase.
3. The price of the imported products in Industry X is also increased by the same rate as the domestic products in Industry X.
4. The price increases shown in this calculation are price increases in the manufacturing sectors only. Price increases in the distribution and retail sectors are not included. For example, for the passenger automotive supply chain, price increases in basic chemicals could increase the price of showroom furniture and fixtures at car dealers, but these increases are not included.

Supplementary figures

Fig. S1.
More detailed explanation of Figure 4 of main text

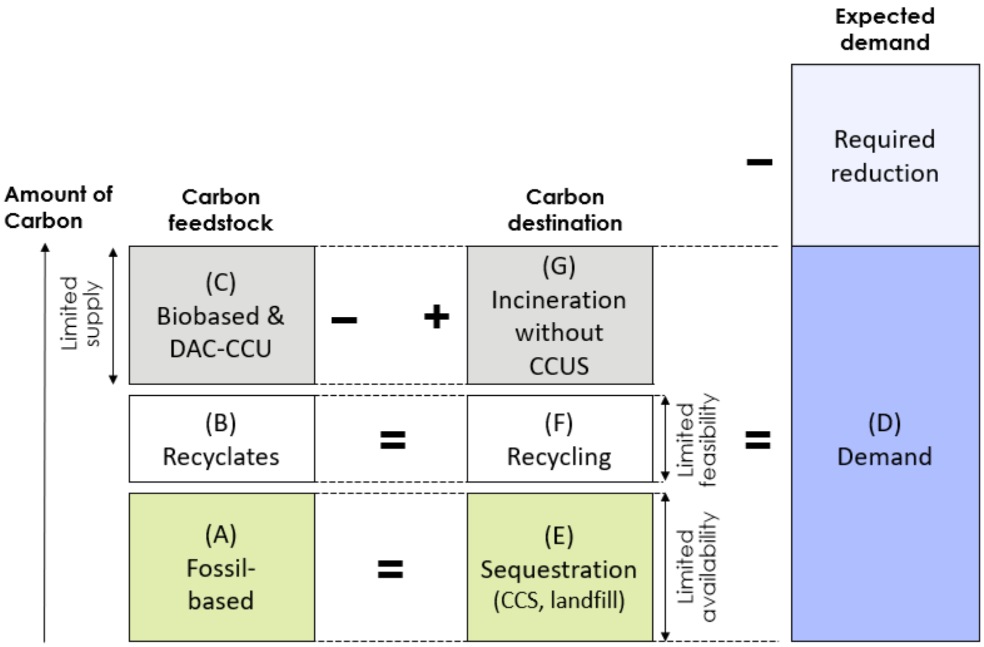

Fig. S2.
Step-by-step approach in the demand model

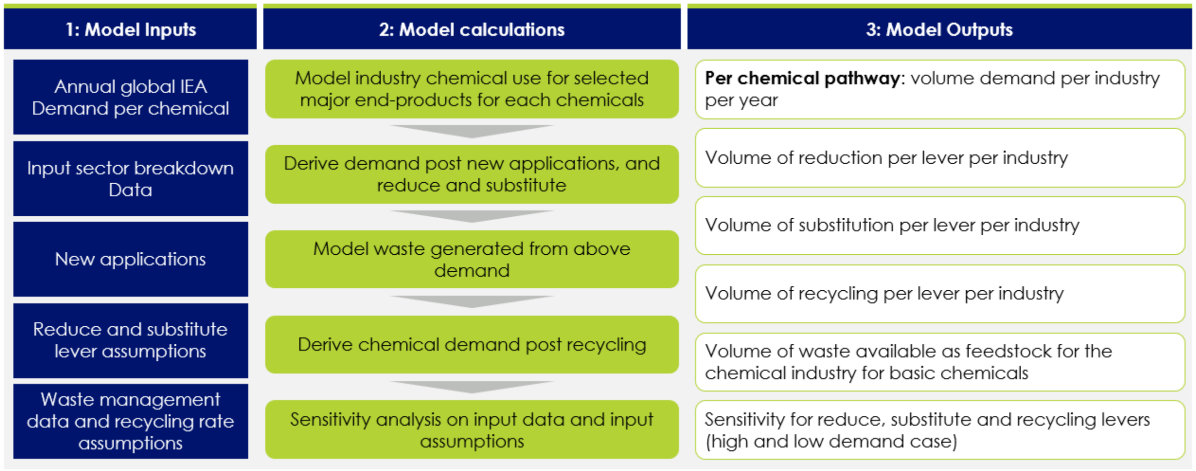

Fig. S3.

Methodology to calculate the main demand of ethylene breakdown by chemicals

Main demand of ethylene breakdown by chemical

%, 2020

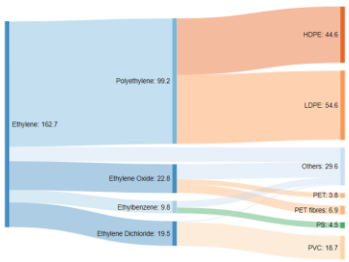

	METRIC	VALUE	METRIC	SOURCES/COMMENTS
OVERALL ethylene PRODUCTION	2017 ethylene demand	150	Mt	IEA – Reference technology scenario, 2018
+	Additional ethylene demand by 2020	13	Mt	IEA – Reference technology scenario, 2018 (CAGR 1.5% across 30 years)
=	2020 ethylene demand	**163**	Mt	
TIERS 1 CHEMICAL BREAKDOWN	% Polyethylene	61	%	Adjusted based on publicly available data from Merchant Consulting Limited, 2020
	% ethylene oxide	14	%	
	% Ethylbenzene	6	%	
	% ethylene dichloride	12	%	
	% Others	7	%	
TIERS 2-3 CHEMICAL BREAKDOWN	% HDPE	45	%	Geyer et Al, 2017, assuming PE is made of 100% ethylene. Calculated out of polyethelyene.
	% LDPE	55	%	
	% PET	17	%	For ethylene oxide to ethylene glycol: Market and Research Consulting 2016. For ethylene glycol to PET and PET fibres: PMR Press Release, 2019. Calculated out of ethylene oxide.
	% PET fibres	30	%	
	% Others	53	%	
	% PVC	96	%	Market Research Future, 2016. Calculated out of ethylene dichloride.
	% Others	4	%	
	% PS	58	%	For ethylbenzene to styrene: Merchant Consulting Limited, 2020. For styrene to PS: Expert Market Research, 2020. Calculated out of ethylbenzene.
	% Others	42	%	

Fig. S4.

Methodology to calculate the main demand of ethylene by industry for 2020 and 2050

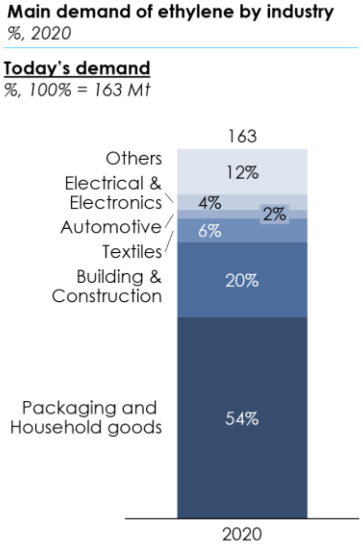

	METRIC	VALUE	METRIC	SOURCES/COMMENTS
OVERALL ethylene PRODUCTION	2017 ethylene demand	150	Mt	IEA – Reference technology scenario, 2018
	+ Additional ethylene demand by 2020	13	Mt	IEA – Reference technology scenario, 2018 (CAGR 1.5% across 30 years)
	= 2020 ethylene demand	**163**	Mt	
INDUSTRY BREKADOWN	% Packaging and household goods	46	%	• Data form Geyer et al., 2017 (including textiles) for all plastics by industry.<br>• Packaging and household goods include institutional products.<br>• For textiles, assuming 100% of ethylene-derived fibres are PET<br>• Others include unspecified.
	% Buildings and construction	15	%	
	% Transportation	6	%	
	% Textiles	19	%	
	% Others	15	%	
ETHYLENE-DERIVED SHARE PER POLYMER RESIN	% ethylene in PE	100	%	• Calculated based on the molar mass of ethylene (28 g.mol-1) over the molar mass of the repeating unit of the polymer.<br>• Other include all other polymer resins unspecified.
	% ethylene in PVC	45	%	
	% ethylene in PET	15	%	
	% ethylene in PS	27	%	
	% ethylene in other	10	%	
INDUSTRY BREKADOWN	% Packaging and household goods	55% , 89 Mt		
	% Buildings and construction	21% , 35 Mt		
	% Transportation	5% , 8 Mt		
	% Textiles	2% , 3 Mt		
	% Others	17% , 27 Mt		

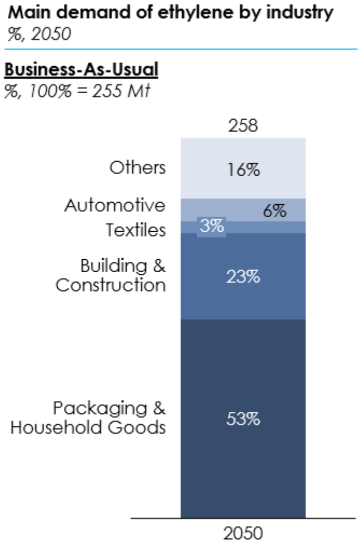

	METRIC	VALUE	METRIC	SOURCES/COMMENTS
OVERALL ethylene PRODUCTION	2017 ethylene demand	150	Mt	IEA – Reference technology scenario, 2018
	+ Additional ethylene demand by 2050	105	Mt	IEA – Reference technology scenario, 2018 (CAGR 1.5% across 30 years)
	= 2050 ethylene demand	**255**	Mt	
INDUSTRY GROWTH CAGR 2020-2050	% Packaging and household goods	1.4	%	• Packaging and household goods: Breaking the plastic Wave, 2020 and divided by 2<br>• Building and Construction and Transportation: Overseas Development Institute, 2020 and divided by 2<br>• Apparel: McKinsey, Fashion On Climate, 2020 and divided by 2<br>• Others (including non-apparel textiles): calculated using remaining ethylene demand from IEA scenario<br>• Overall CAGRs were adjusted consistently (divided by 2) from sources to match IEA overall projections
	% Buildings and construction	1.7	%	
	% Transportation	1.7	%	
	% Apparel	2.1	%	
	% Electrical and Electronics	1.5	%	
	% Others	1.5	%	
INDUSTRY BREKADOWN	% Packaging and household goods	53	%	135 Mt
	% Buildings and construction	23	%	58 Mt
	% Transportation	6	%	14 Mt
	% Textiles	3	%	6 Mt
	% Others	16	%	41 Mt

Fig. S5.
Methodology to calculate annual demand for ethylene for packaging and household goods post reduce and substitute levers

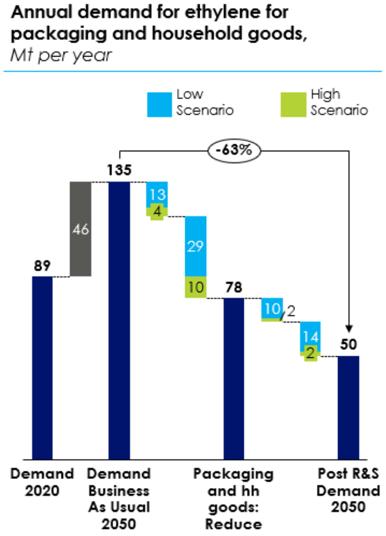

	METRIC	VALUE	METRIC			SOURCES/COMMENTS
INDUSTRY DEMAND	2050 packaging and household goods demand	135	Mt			Previously calculated
REDUCE LEVERS		Elimination		New Business Models and reuse		• % are applied directly to total sector demand<br>• Analysis from Breaking the Plastic Wave, 2020 by packaging application and taking into account polymer resin content of each application extrapolated from 2040 to 2050<br>• High scenario assuming same reduction potential by 2040 than Breaking the Plastic Wave and higher penetration market by 2050 of reduce solutions (S-curve applied)<br>• Low scenario assuming same reduction potential than Breaking the Plastic Wave but achieved by 2050 instead of 2040
	High scenario					
	By 2030	1.0	%	5.8	%	
	By 2040	6.8	%	17.2	%	
	By 2050	12.6	%	29.2	%	
	Low scenario					
	By 2030	0.7	%	4.3	%	
	By 2040	5.0	%	12.7	%	
	By 2050	9.3	%	21.6	%	
SUBSTITUION LEVERS		Paper		Compostables		
	High scenario					
	By 2030	2.6	%	3.9	%	
	By 2040	5.8	%	8.1	%	
	By 2050	8.6	%	12.1	%	
	Low scenario					
	By 2030	2.2	%	3.4	%	
	By 2040	5.1	%	7.1	%	
	By 2050	7.5	%	10.5	%	

Fig. S6.
Methodology to calculate annual demand for ethylene for transportation post reduce and substitute levers

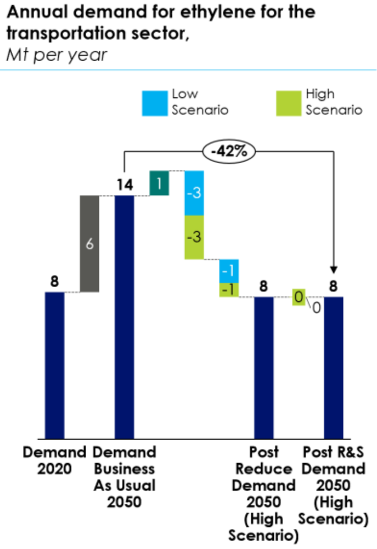

	METRIC	VALUE	METRIC	SOURCES/COMMENTS
INDUSTRY DEMAND	2050 transportation demand	14	Mt	Previously calculated
SWITCH FROM ICE TO EV	High scenario			• Based on ETC analysis and provided by the author (not published) • % represent the penetration rate of EV as part of the global market • Then it was assumed that EV have 10% more plastic than ICE based on an analysis from WEF: Forging Ahead, 2020
	By 2030	20	%	
	By 2040	70	%	
	By 2050	100	%	
	Low scenario			
	By 2030	10	%	
	By 2040	50	%	
	By 2050	100	%	
REDUCE LEVERS	High scenario	MaaS	Lifetime Ext.	• % are applied directly to total sector demand • Analysis based on forth coming XaaS report, 2021 from SYSTEMIQ • Low scenario was assumed to have a 50% ambition level compare to high scenario
	By 2030	3 %	2 %	
	By 2040	13 %	8 %	
	By 2050	33 %	21 %	
	Low scenario			
	By 2030	2 %	1 %	
	By 2040	7 %	4 %	
	By 2050	17 %	11 %	
SUBSTITUION LEVERS				• No substitution is expected in this sector according to Phasing out Plastic – The automotive sector, 2020, ODI and confirmed by industry experts

Fig. S7.
Methodology to calculate annual demand for ethylene for buildings and construction post reduce and substitute levers

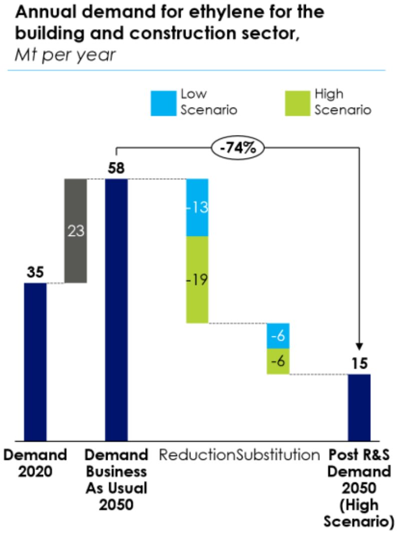

	METRIC	VALUE	METRIC	SOURCES/COMMENTS
INDUSTRY DEMAND	2050 buildings and construction demand	58	Mt	Previously calculated
REDUCE LEVERS	**High scenario**<br>By 2030<br>By 2040<br>By 2050<br>**Low scenario**<br>By 2030<br>By 2040<br>By 2050	Reduction<br>**18**<br>**36**<br>**55**<br><br>**7**<br>**15**<br>**22**	 %<br>%<br>%<br><br>%<br>%<br>%	• % are applied directly to total sector demand<br>• Analysis from Phasing out Plastic – The construction sector, 2020, ODI<br>• Adjustment were made as followed:<br>• In a high scenario, substitution of flooring, cabling and liners was capped at 90% penetration rate by 2050 (similar substitution rate as per packaging)<br>• Substitution of pipes, tubes and fittings were assumed not recommended given material of substitution has prohibitive GHG emissions (e.g. aluminium or steel)
SUBSTITUION LEVERS	**High scenario**<br>By 2030<br>By 2040<br>By 2050<br>**Low scenario**<br>By 2030<br>By 2040<br>By 2050	Substitution<br>**6**<br>**13**<br>**19**<br><br>**3**<br>**6**<br>**10**	 %<br>%<br>%<br><br>%<br>%<br>%	• Substitution of profiles was capped at 50% by 2050 given higher cost of substitute wood material (similar assumption that packaging when applied) and accounting for the fact that substitution with aluminium and steel is not recommended (see above)<br>• Low scenario for reduction is 40% of high scenario and based on IRP scenario for construction material reduction targets. 50% was estimated for substitution<br>• The penetration of the levers is assumed to be linear between 2020 and 2050 given there is no technology barrier to existing solutions.

Fig. S8.
Methodology to calculate annual demand for ethylene for apparel post reduce and substitute levers

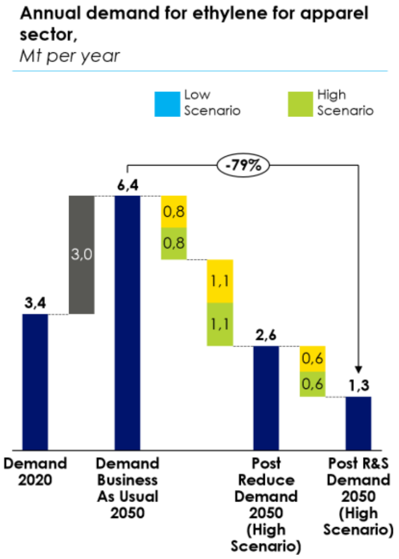

	METRIC	VALUE	METRIC			SOURCES/COMMENTS
INDUSTRY DEMAND	2050 apparel demand	6	Mt			Previously calculated
REDUCE LEVERS		Waste reduction		New business models		
	High scenario					
	By 2030	18	%	7	%	• Analysis from Fashion for Climate, 2019, McKinsey extrapolated to 2050.
	By 2040	23	%	17	%	
	By 2050	25	%	34	%	
	Low scenario					
	By 2030	9	%	3	%	
	By 2040	11	%	19	%	
	By 2050	13	%	17	%	
SUBSTITUION LEVERS		Substitution				
	High scenario					
	By 2030	7	%			• Market analysis on average of various report found man-made fibres are going to grow at a CAGR twice superior to other fibres (2.1% - see previously) and additional growth will lead to substitution of cotton and polyester fibres equally.
	By 2040	13	%			
	By 2050	20	%			
	Low scenario					
	By 2030	3	%			
	By 2040	7	%			
	By 2050	10	%			

Fig. S9.
Methodology to calculate annual demand for ethylene for other sectors post reduce and substitute levers

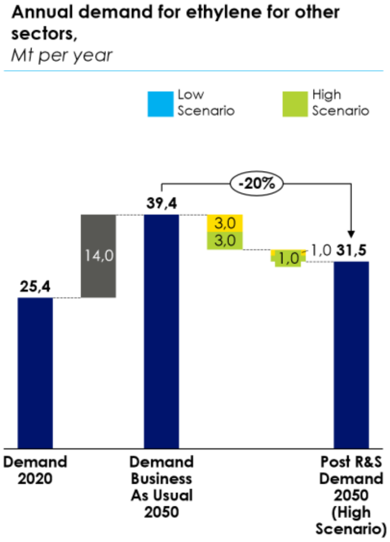

	METRIC	VALUE	METRIC	SOURCES/COMMENTS
INDUSTRY DEMAND	2050 other sectors demand	34	Mt	Previously calculated
REDUCE LEVERS	High scenario			• Reduction and substitution innovation is believed to benefit all sectors and therefore by default it was assumed to be at least similar to the most conservative assumptions for other sectors
	By 2030	5	%	
	By 2040	10	%	
	By 2050	15	%	
	Low scenario			
	By 2030	2	%	
	By 2040	3	%	
	By 2050	5	%	
SUBSTITUION LEVERS	High scenario			
	By 2030	3	%	
	By 2040	5	%	
	By 2050	8	%	
	Low scenario			
	By 2030	1	%	
	By 2040	2	%	
	By 2050	3	%	

Fig. S10.
Methodology to calculate HC ethylene-derived waste generation

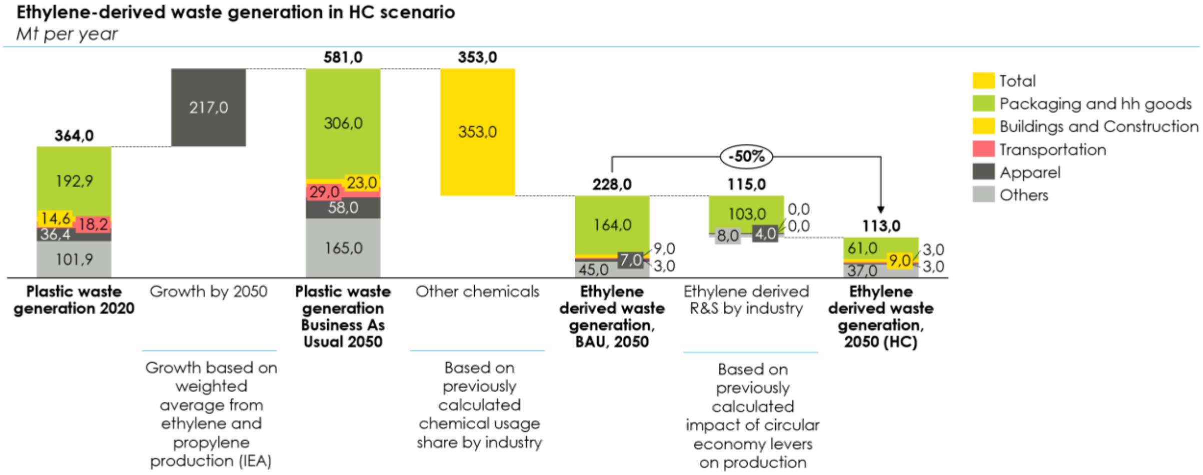

Fig. S11.
Recycling rates assumptions for each sector across HC and LC scenarios

Projected Recycling rates
%

	2020			2050 - LC			2050 - HC		
	Overall plastic	Ethylene-adjusted	Propylene-adjusted	Overall plastic	Ethylene-adjusted	Propylene-adjusted	Overall plastic	Ethylene-adjusted	Propylene-adjusted
**Packaging and household goods**	**14%**	15%	6%	**42%**	42%	43%	**57%**	57%	46%
**Buildings and construction**	**2%**	2%	1%	**50%**	55%	40%	**50%**	55%	40%
**Transportation**	**3%**	3%	3%	**40%**	40%	45%	**44%**	48%	48%
**Apparel**	**2%**	13%	n/a	**26%**	26%	n/a	**50%**	50%	n/a
**Others**	**5%**	5%	5%	**18%**	18%	18%	**20%**	20%	20%
**Overall**	**10%**	**12%**	**5%**	**37%**	**47%**	**35%**	**43%**	**44%**	**35%**

Fig. S12.
Origin and fate of recycled outputs from ethylene-derived plastic waste

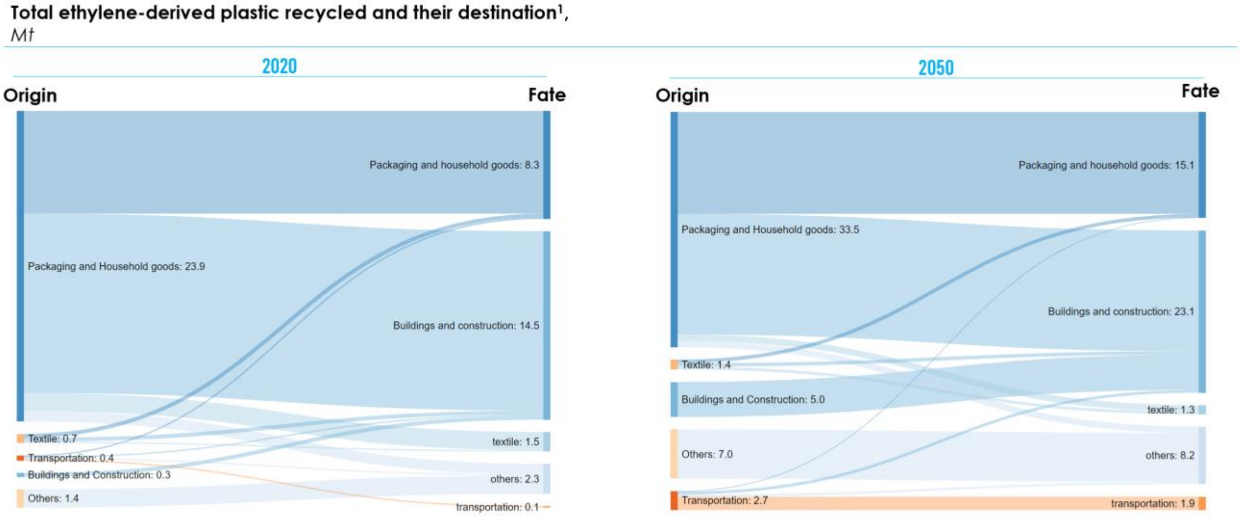

Fig. S13.
Step-by-step approach in the supply model

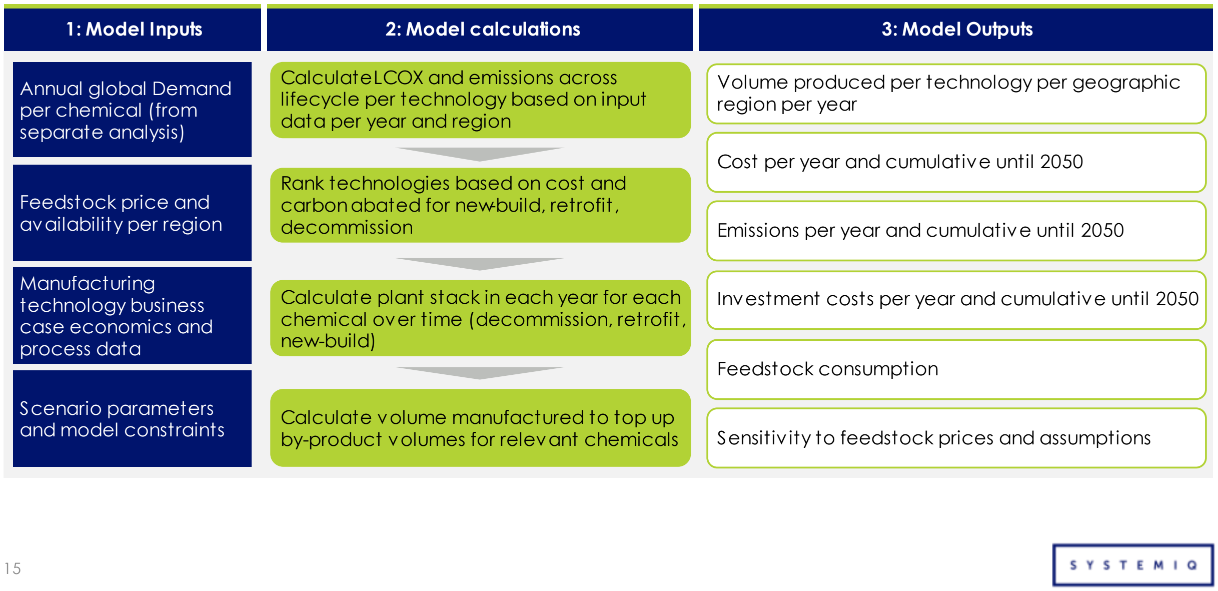

Fig. S14.
Description of ranking methodology for different supply scenarios

Scenarios		Business as usual (BAU)	Most economic (ME)	No fossil new-build after 2030 (NFAX)	No Fossil Strict (NFS)
Pathways		• Business-As-Usual Scenario (BDEM – BAU)	• Net-Zero Business-As-Usual Scenario (BDEM – ME)<br>• Most Economic Scenario (LC – ME)<br>• Circular Economy Scenario (HC – ME	• No New Fossil Scenario (LC – NFAX)<br>• System change scenario (HC – NFAX)	• Radical system change scenario (HC – NFS)
Retrofit existing plants	Retrofitting modelling logic	No retrofitting required	• Fixed retrofit rate: 5% of production volume per year undergoes retrofit<br>• Initial technologies are preferentially retrofitted<br>• Plants can be retrofitted only once, except for 'transition technology' which are not full abated after the first retrofit<br>• Retrofit rankings include decommissioning of existing plant and construction of abated plant		
Build new plants if demand > production capacity	Ranking logic for retrofits and addition of new plant capacity to meet demand	#1 criteria is used by default, #2 is used when two technology have similar criteria for #1, similarly #3 is used when two technologies have similar criteria for #1 and #2			
		1. Minimum LCOX<br>2. Maximum abatement of production emissions<br>3. Maximum abatement of raw-material emissions	1. End-state technologies ranked before transitional and initial technologies<br>2. Minimum LCOX<br>3. Maximum abatement of production emissions<br>4. Maximum abatement of raw-material emissions	1. End-state technologies ranked before transitional and initial technologies<br>2. Maximum abatement of sum of production & raw materials emissions<br>3. Minimum LCOX	1. End-state technologies ranked before transitional and initial technologies<br>2. Maximum abatement of sum of production & raw materials emissions<br>3. Minimum LCOX
	Extra constraints for ranking	• Conventional technology (currently in use) allowed until 2050	• Conventional technology (currently in use) allowed until 2025 considering existing projects in pipeline		
				• Technologies using fossil feedstocks for raw material or energy provision removed from ranking (i.e., not available) after 2030, except initial tech plants until 2025	• Technologies using fossil feedstocks for raw material or energy provision removed from ranking (i.e., not available) except initial tech plants until 2025
Decommission plants	Decommissi on logic	If demand>supply remove in following order:<br>1. Most expensive asset (highest LCOX)<br>2. Highest production emission<br>3. Highest raw material emissions	If demand>supply remove in following order:<br>1. Initial tech first<br>2. Most expensive asset (highest LCOX)<br>3. Highest production emission<br>4. Highest raw material emissions	If demand>supply remove in following order:<br>1. Initial tech first<br>2. Highest production emission<br>3. Highest raw material emissions<br>4. Most expensive asset (highest LCOX)	If demand>supply remove in following order:<br>1. Initial tech first<br>2. Highest production emission<br>3. Highest raw material emissions<br>4. Most expensive asset (highest LCOX)
			Unabated plants are decommissioned starting from 2035 with increasing rate		

Fig. S15.
Supply model logic map

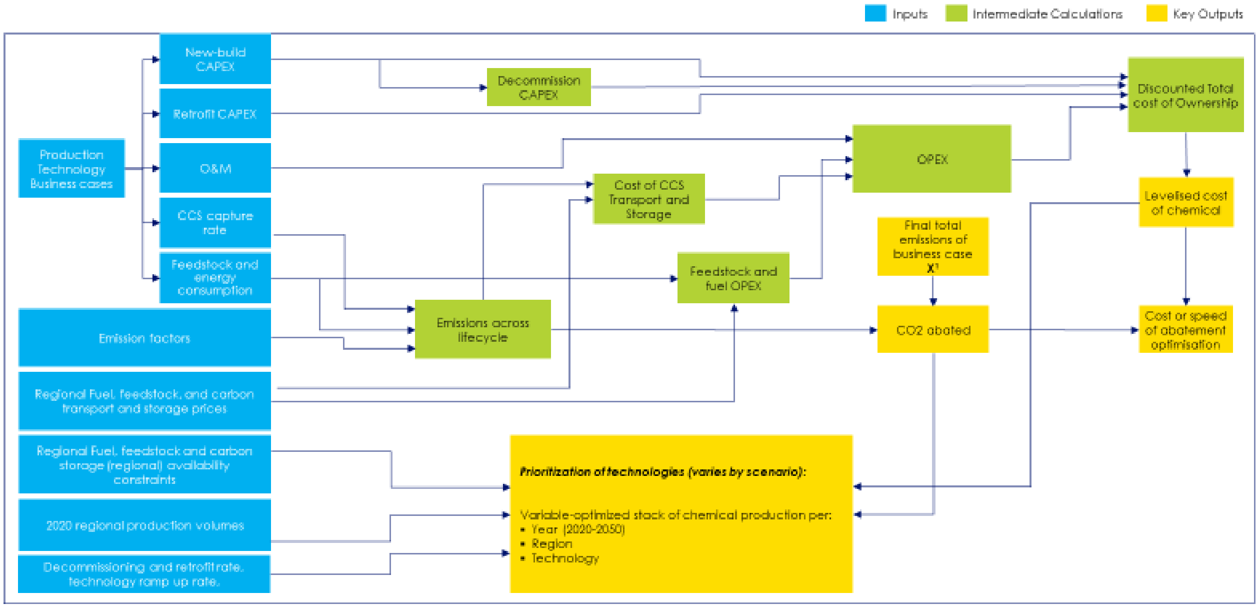

Fig. S16.
Iterative Supply Stack Building to Meet Annual Demand

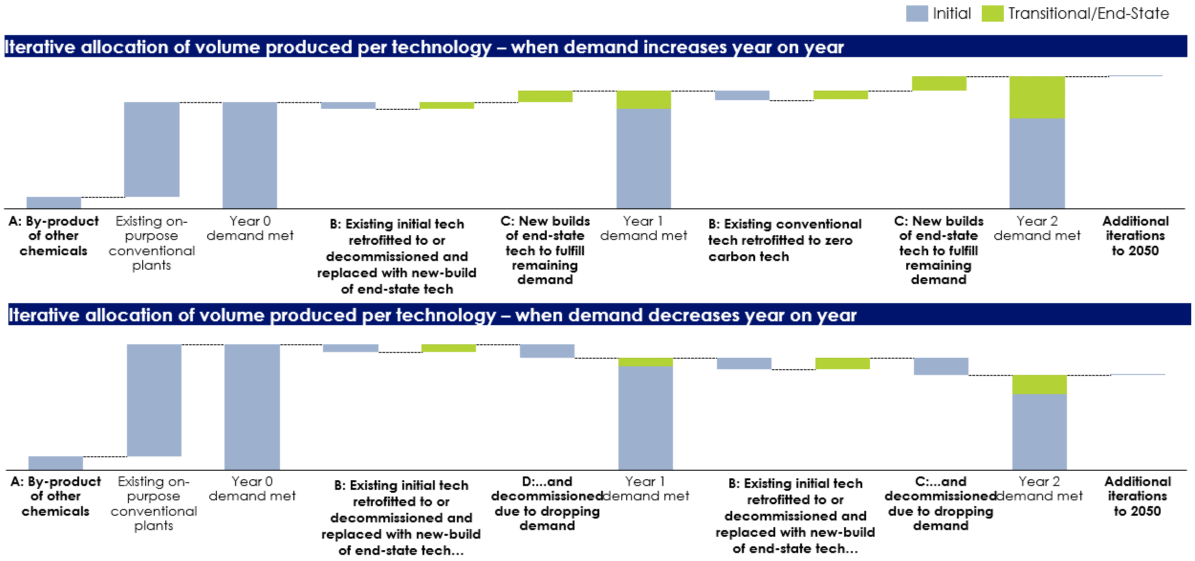

Fig. S17.
Electrolyzer cost decline based on average forecasts from (39)

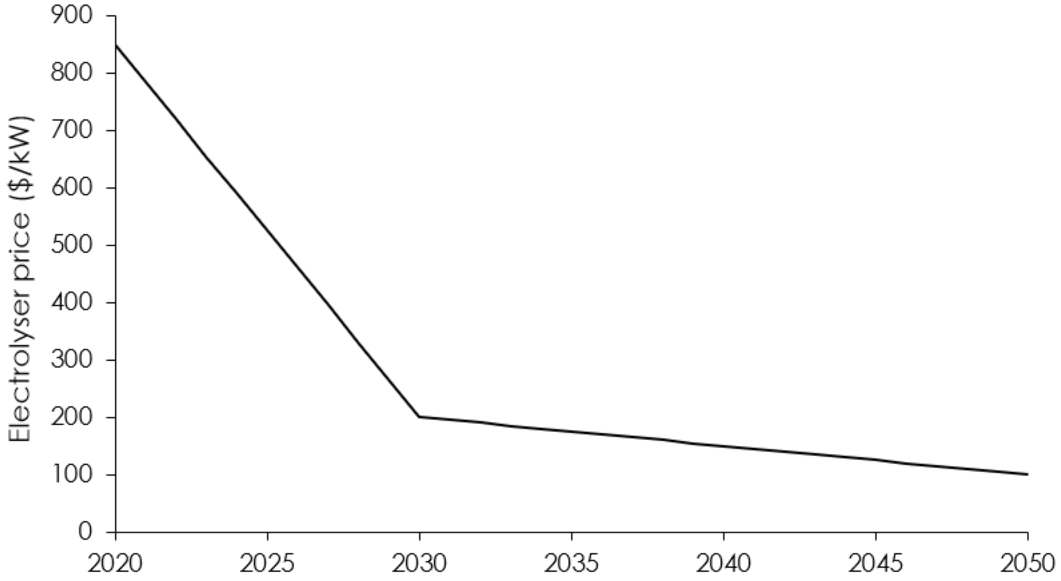

Fig. S18.
Hydrogen price over time produced via dedicated renewable site of hybrid wind and solar including costs for transport and storage (falling from 0.36 to 0.24 $/kg from 2020 to 2050). The indicated prices illustrate average locations for renewables in each region. Prices based on own modelling with input data largely from (39, 47)

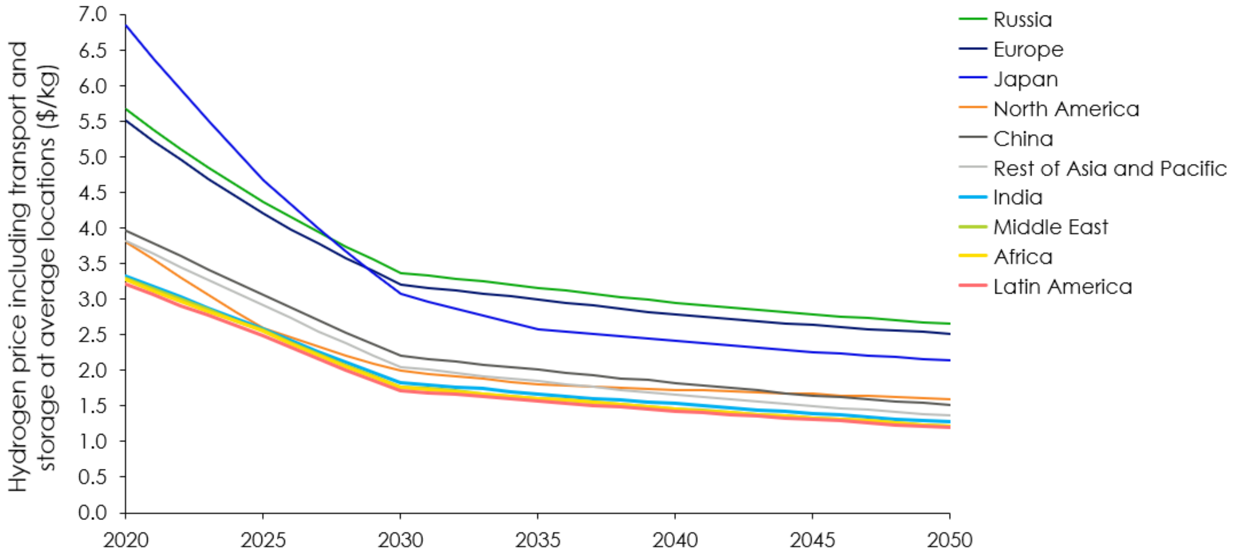

Fig. S19.
Overview of initial, transition and end-state technologies with their associated timeline of availability. Abbreviations: Abbreviations: SMR – steam methane reforming, HB – Haber-Bosch, AN – ammonium nitrate

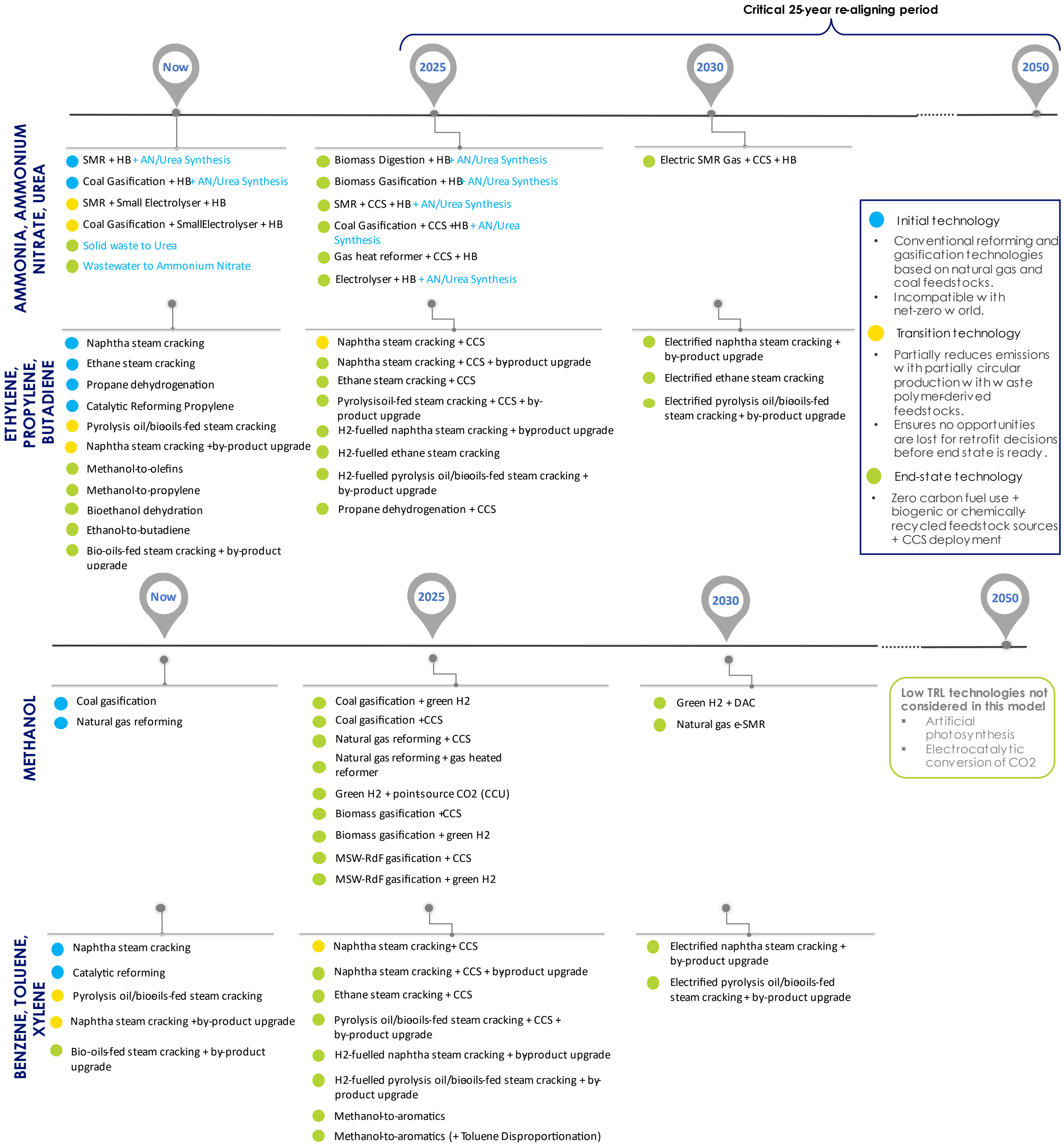

Fig. S20.
Assumed grid emissions factors in BAU and ME, NFAX and NFS scenarios.

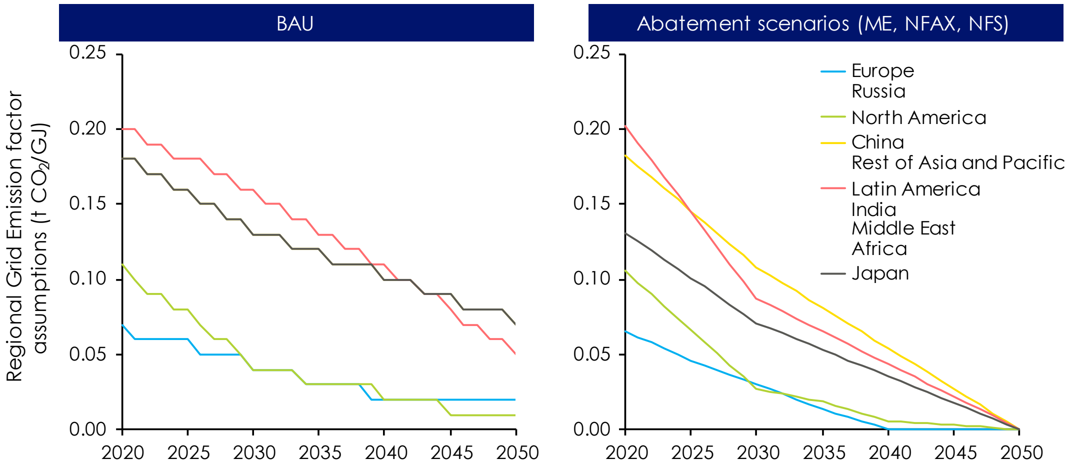

Fig. S21.

Naphtha price for high price (default) and low price (sensitivity)

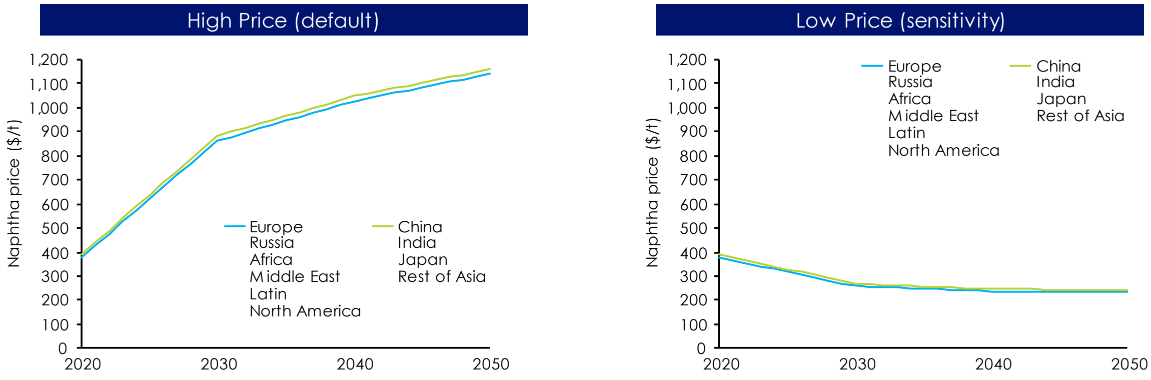

Fig. S22.
Natural gas price for high price (default) and low price (sensitivity) including price spike in early 2020s

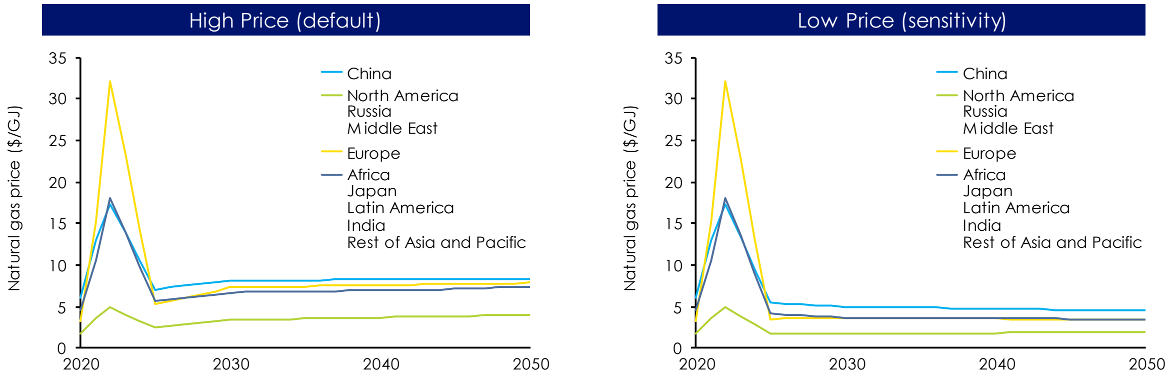

Fig. S23.
Coal price for high price (default) and low price (sensitivity) including price spike in early 2020s

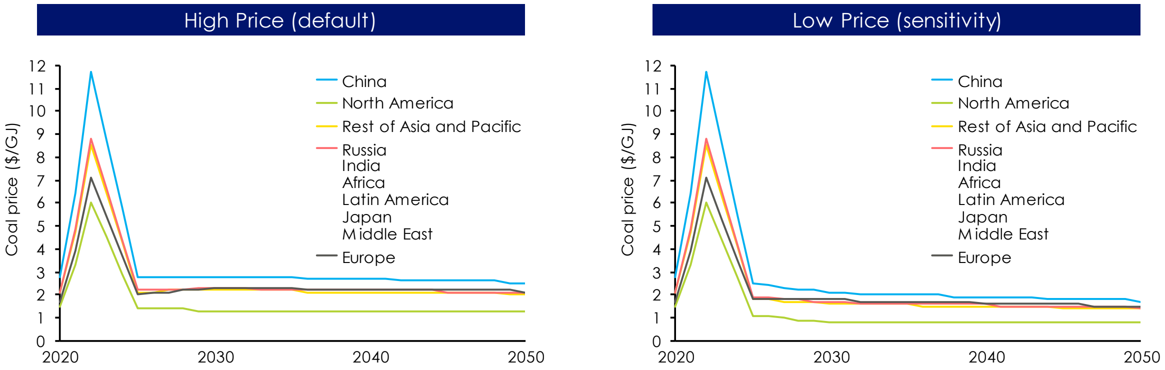

Fig. S24.

Electricity pricing and capacity factors employed for hybrid wind/solar dedicated renewables plant. Energy crisis effects are priced in for grid-ppa prices in the early 2020s.

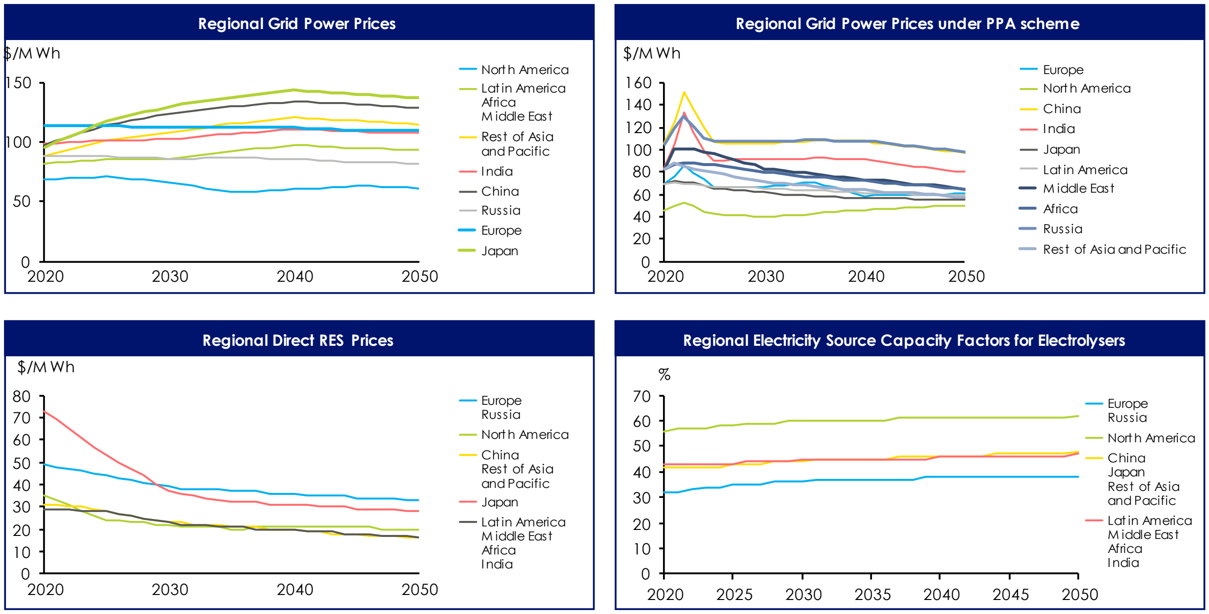

Fig. S25.
Cap of CCS storage volume per year by region

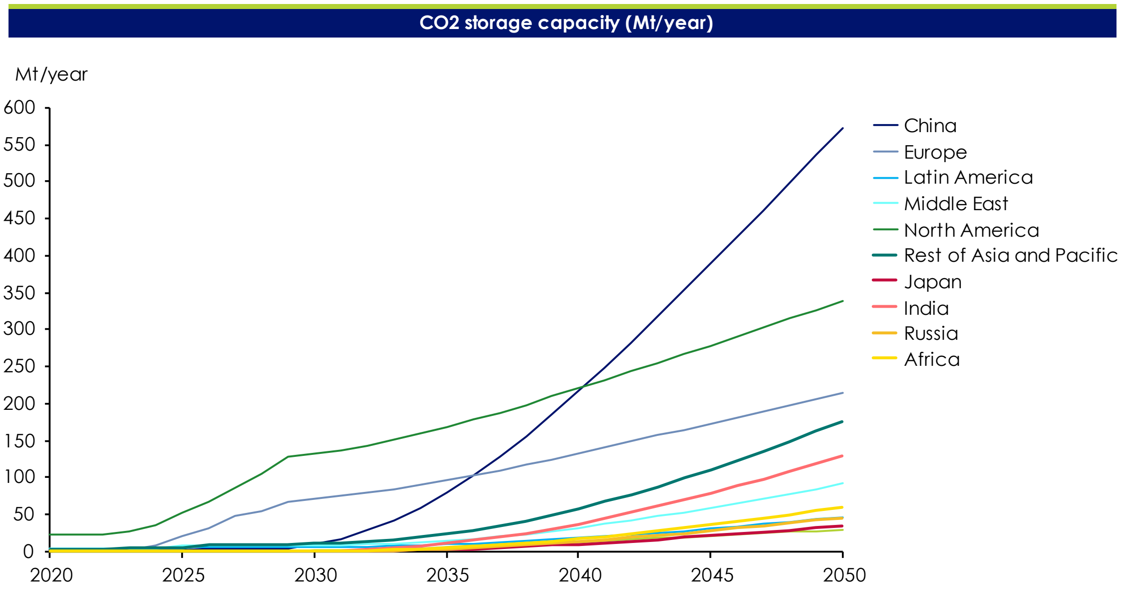

Fig. S26.
The share (top) and absolute volumes (Mt) of CCS used by ammonia, ethylene and methanol respectively in the BDEM-ME scenario. CCS technologies are only available from 2025 onwards. The cap grows significantly in the 2030s-2040s due to widespread CCS deployment towards net-zero and therefore plays a particularly important role in the early stages of the transition.

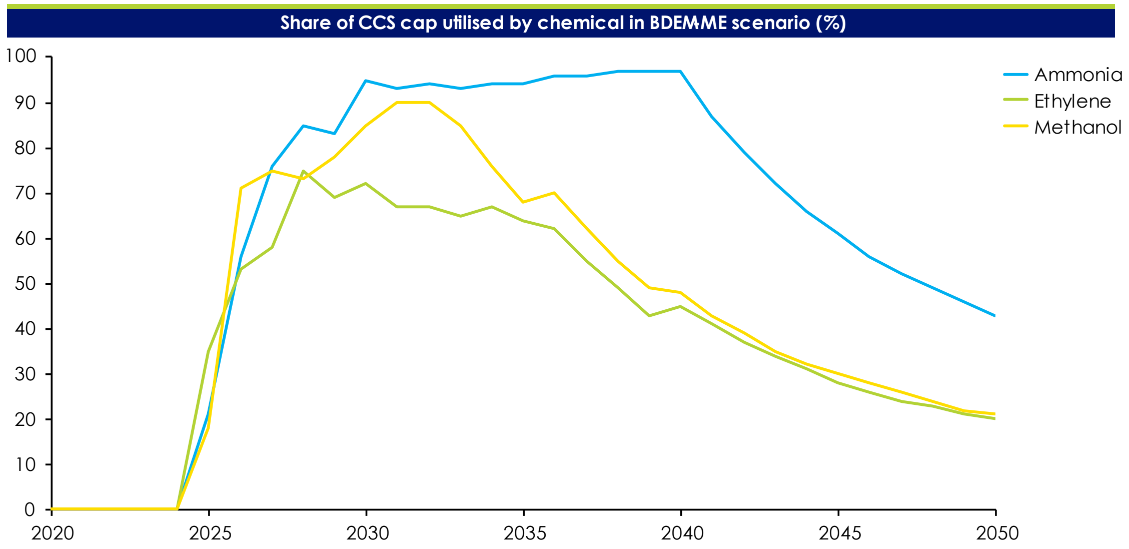

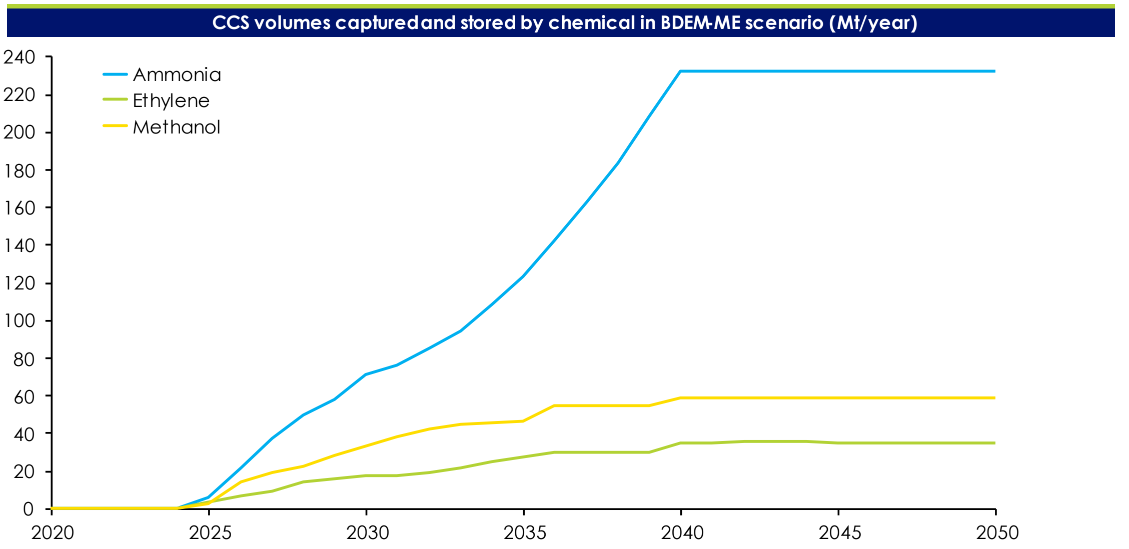

Fig. S27.
Appropriation of costs and emissions from volume-based output of Naphtha steam cracker

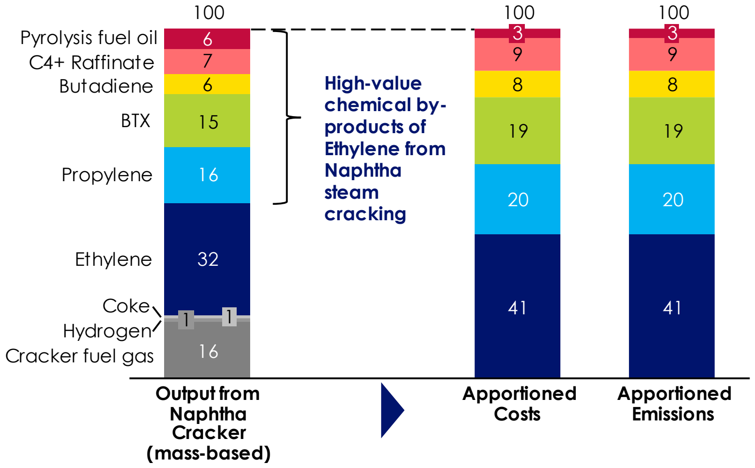

Fig. S28.
Technology over time for ammonia demand and volumes under BDEM-ME scenario

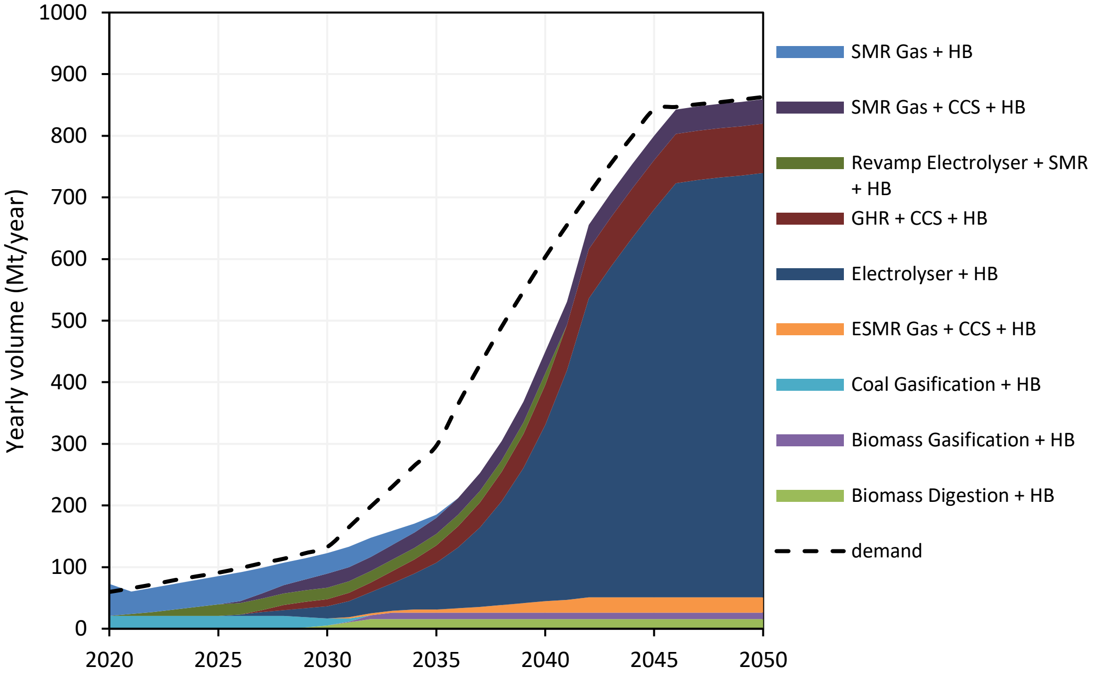

Fig. S29.
Technology over time for ammonium nitrate demand and volumes under BDEM-ME scenario

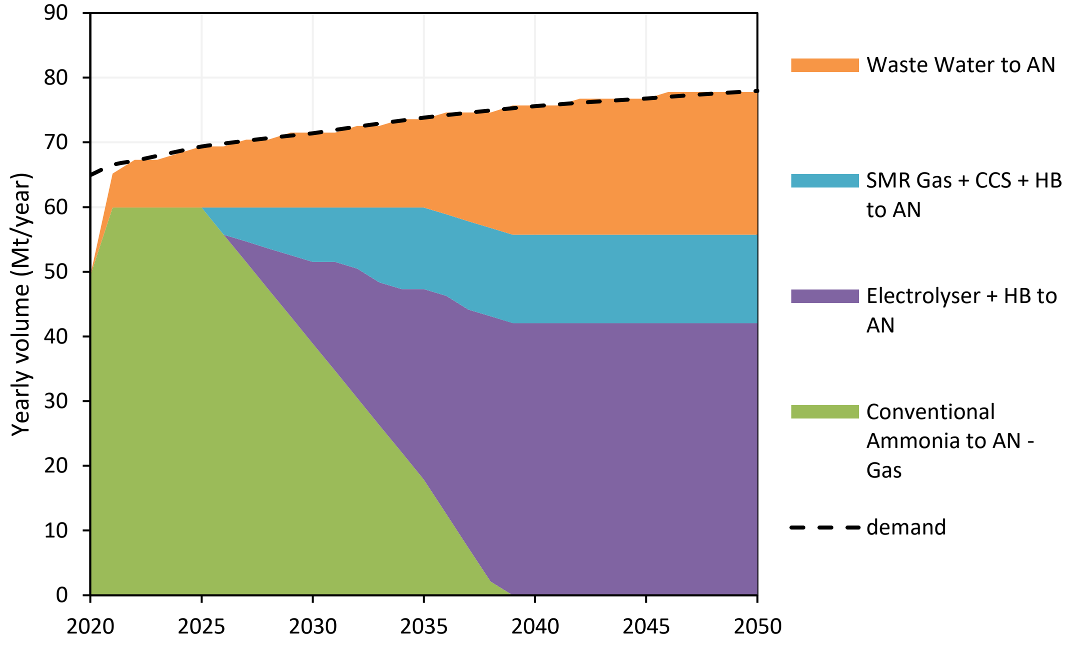

Fig. S30.
Technology over time for benzene demand and volumes under BDEM-ME scenario

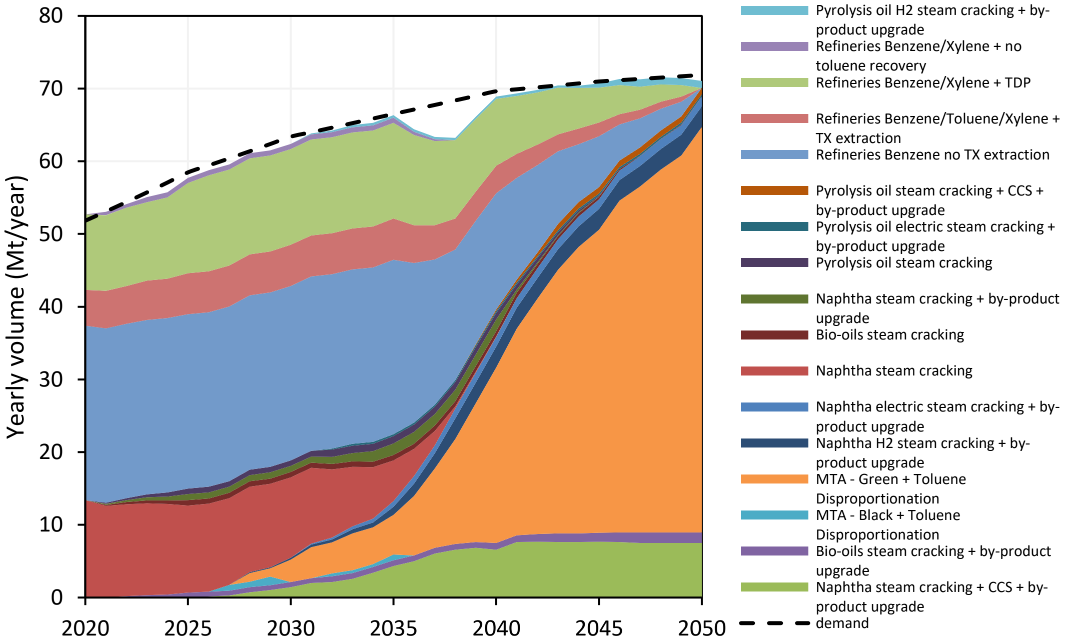

Fig. S31.
Technology over time for butadiene demand and volumes under BDEM-ME scenario

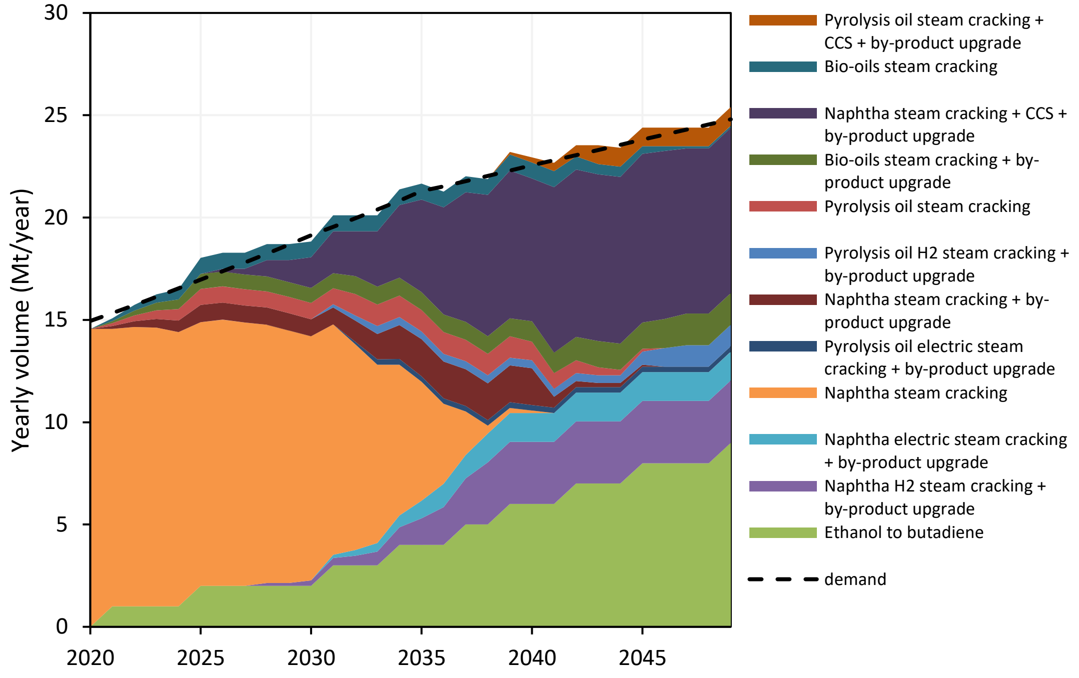

Fig. S32.
Technology over time for ethylene demand and volumes under BDEM-ME scenario

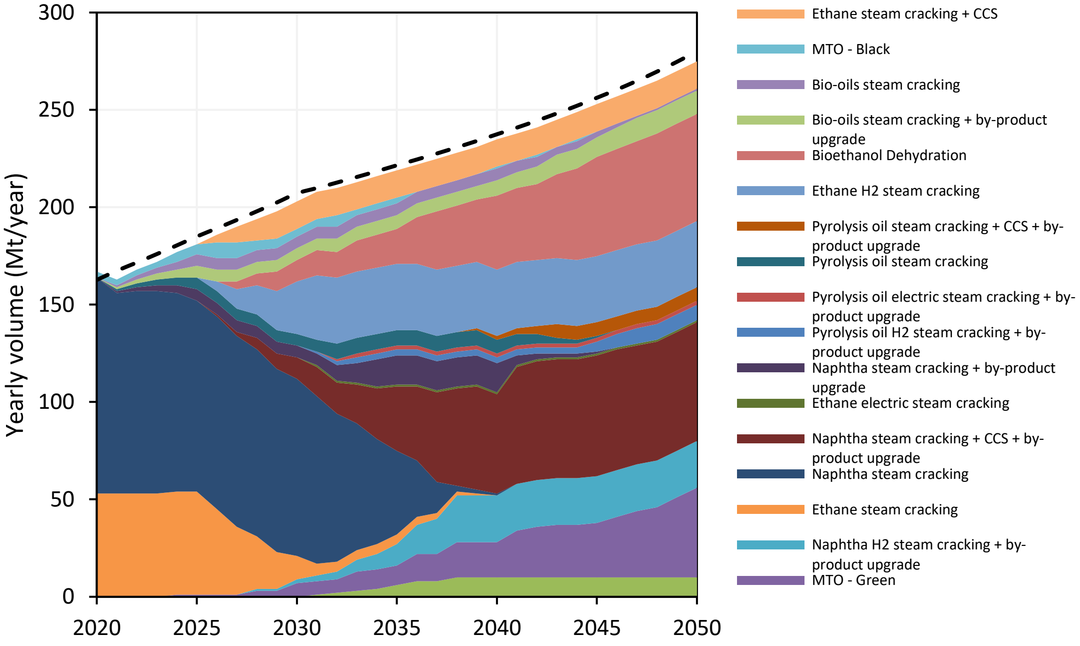

Fig. S33.
Technology over time for methanol demand and volumes under BDEM-ME scenario

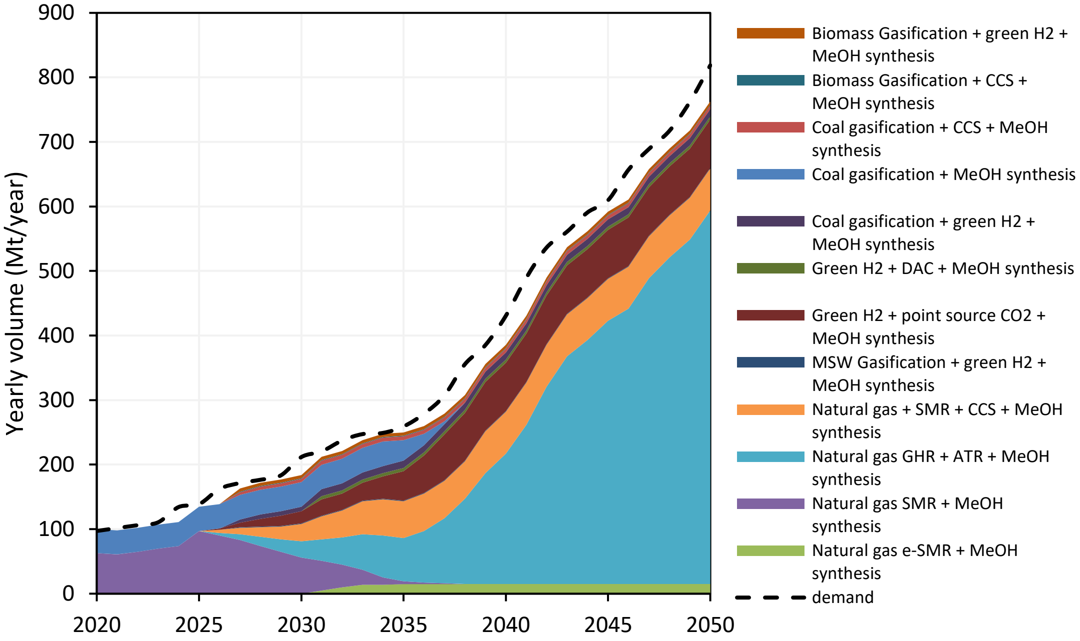

Fig. S34.
Technology over time for propylene demand and volumes under BDEM-ME scenario

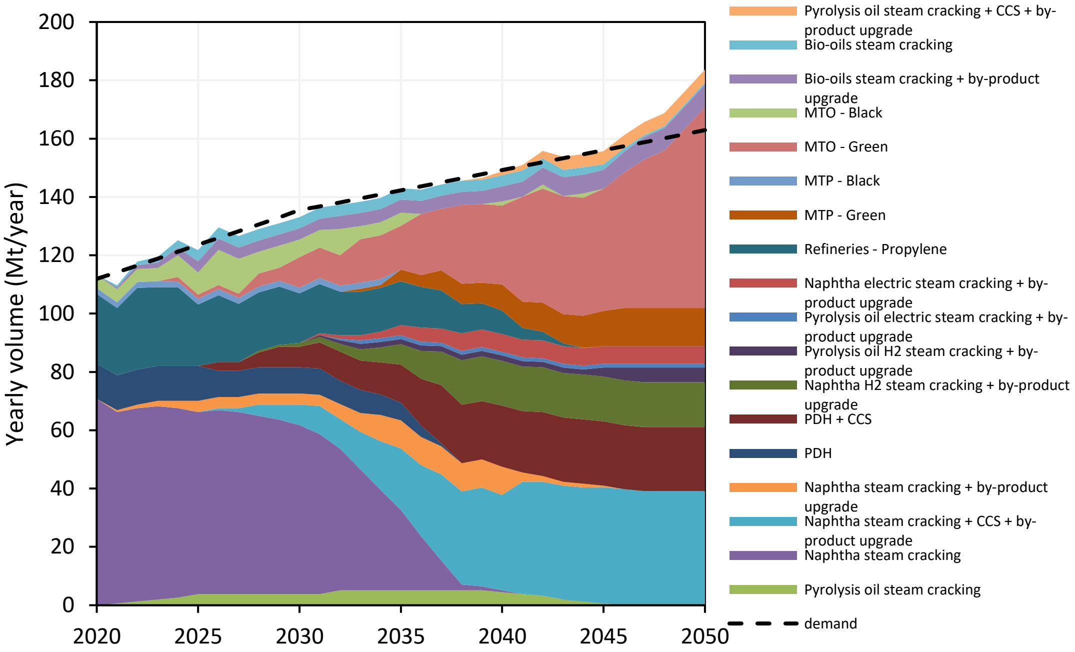

Fig. S35.
Technology over time for toluene demand and volumes under BDEM-ME scenario

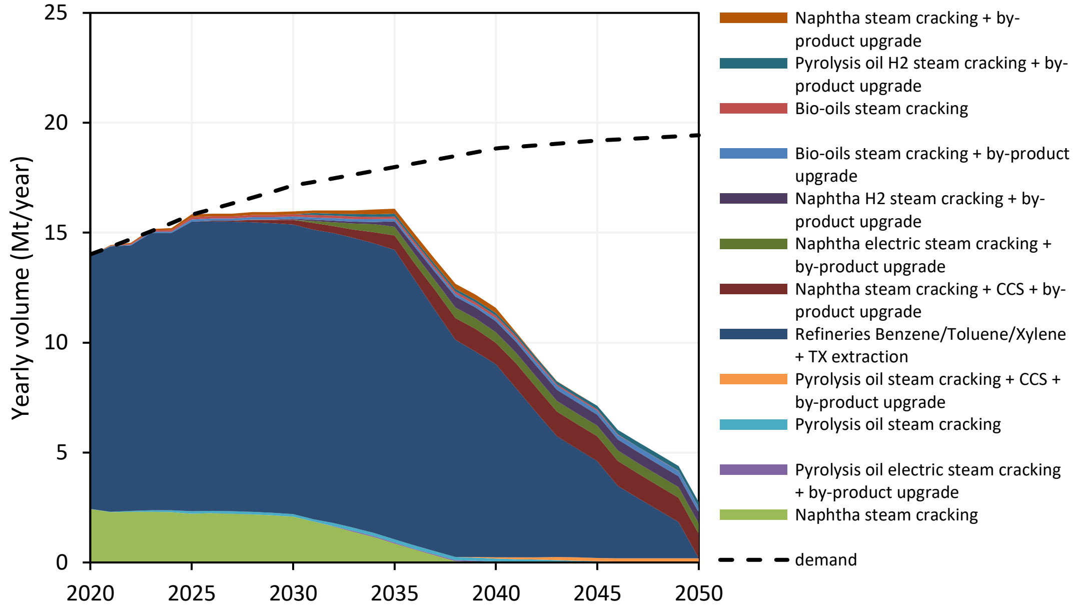

Fig. S36.
Technology over time for urea demand and volumes under BDEM-ME scenario

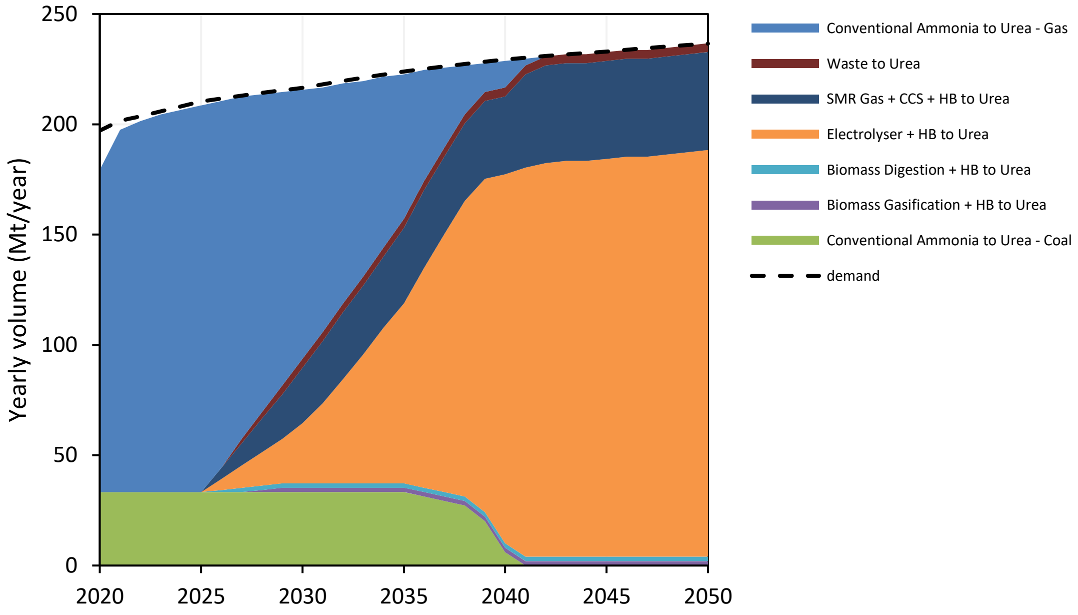

Fig. S37.
Technology over time for xylene demand and volumes under BDEM-ME scenario

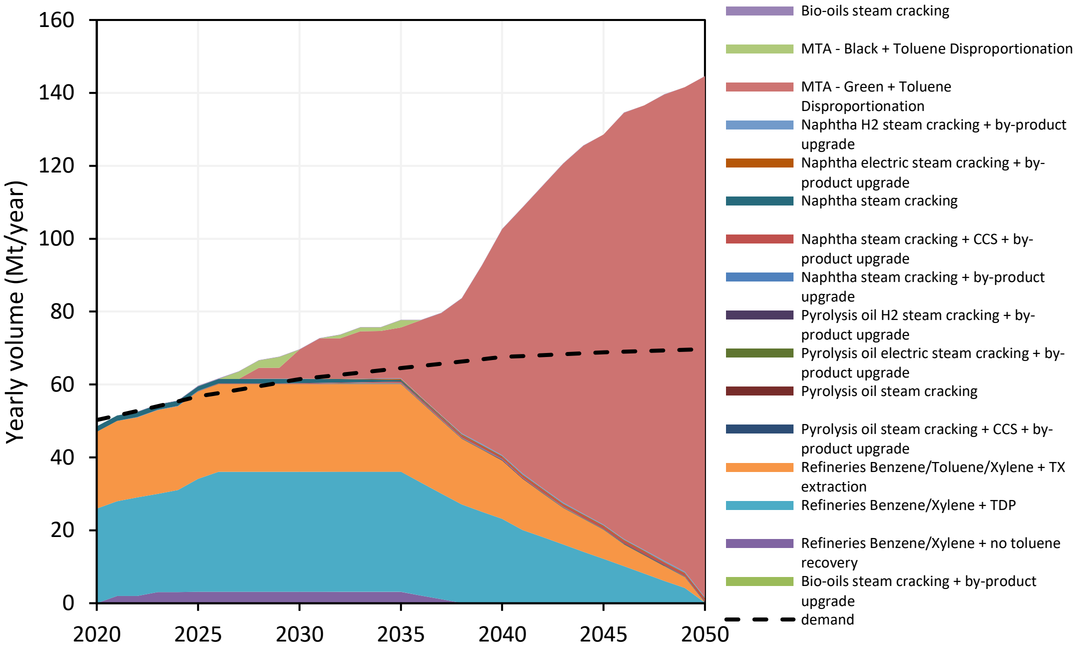

Fig. S38.
Technology over time for ammonia demand and volumes under LC-ME scenario

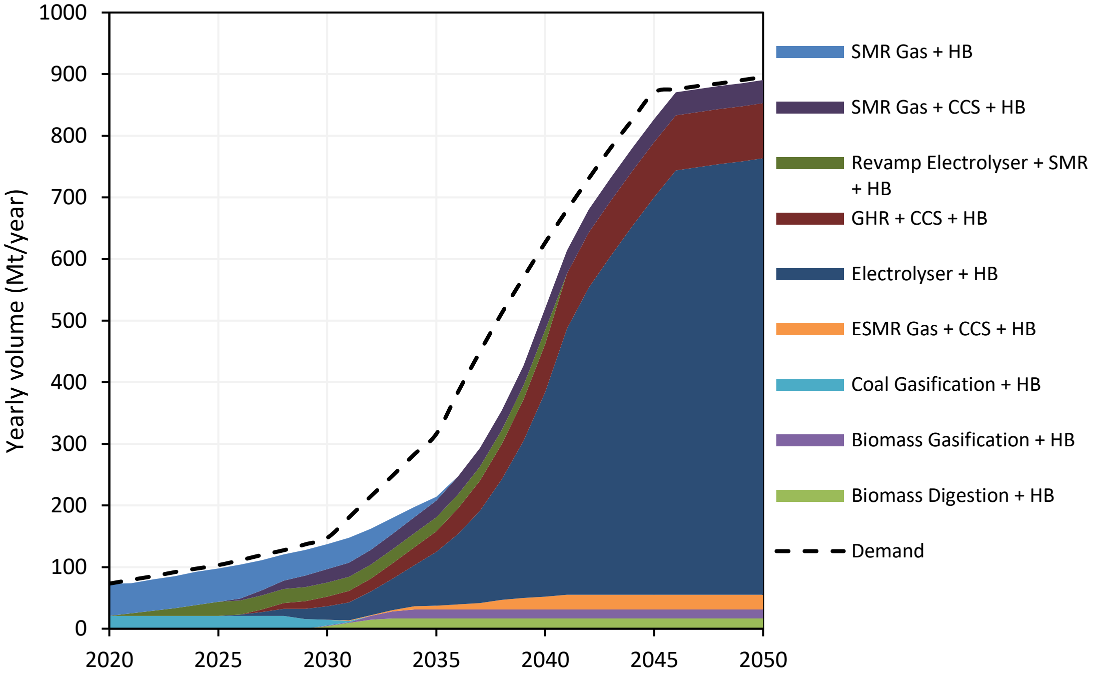

Fig. S39.
Technology over time for ammonium nitrate demand and volumes under LC-ME scenario

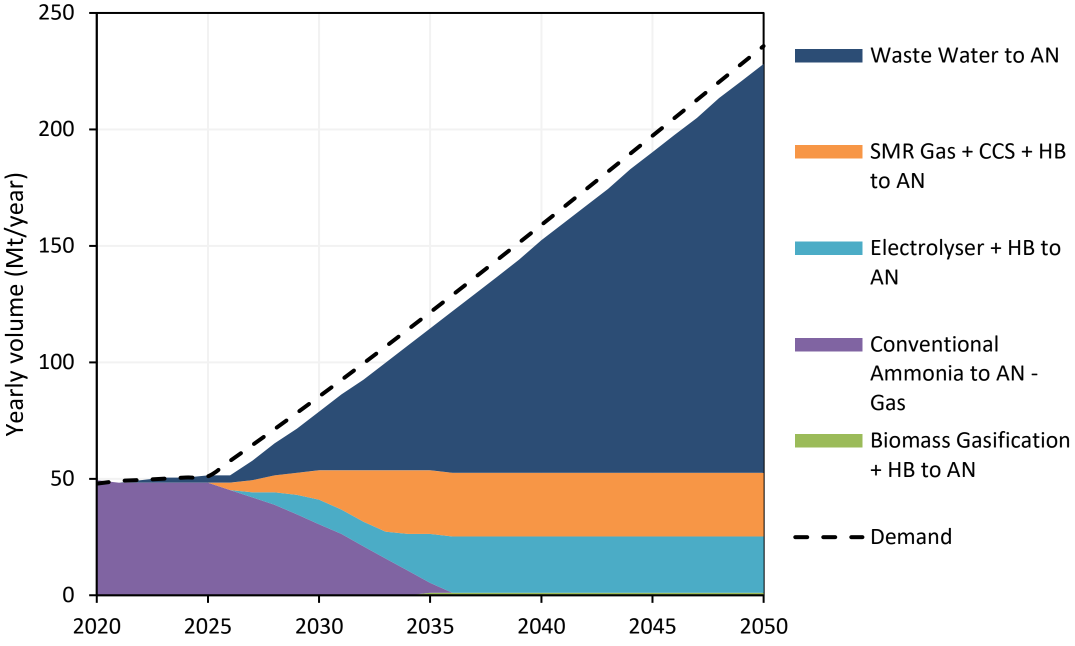

Fig. S40.
Technology over time for benzene demand and volumes under LC-ME scenario

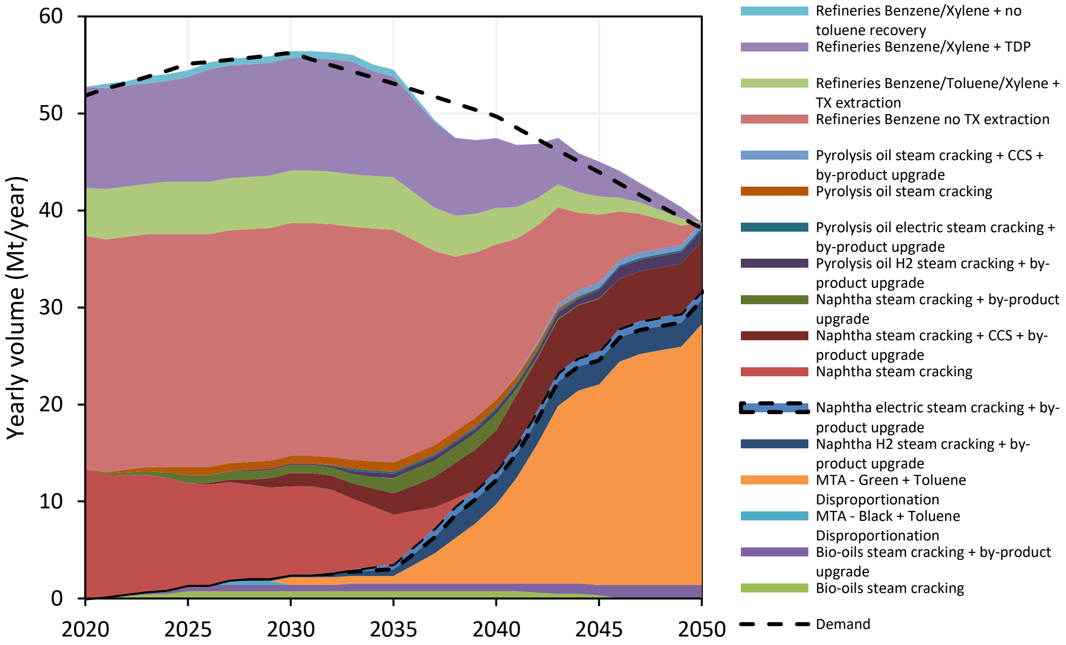

Fig. S41.
Technology over time for butadiene demand and volumes under LC-ME scenario

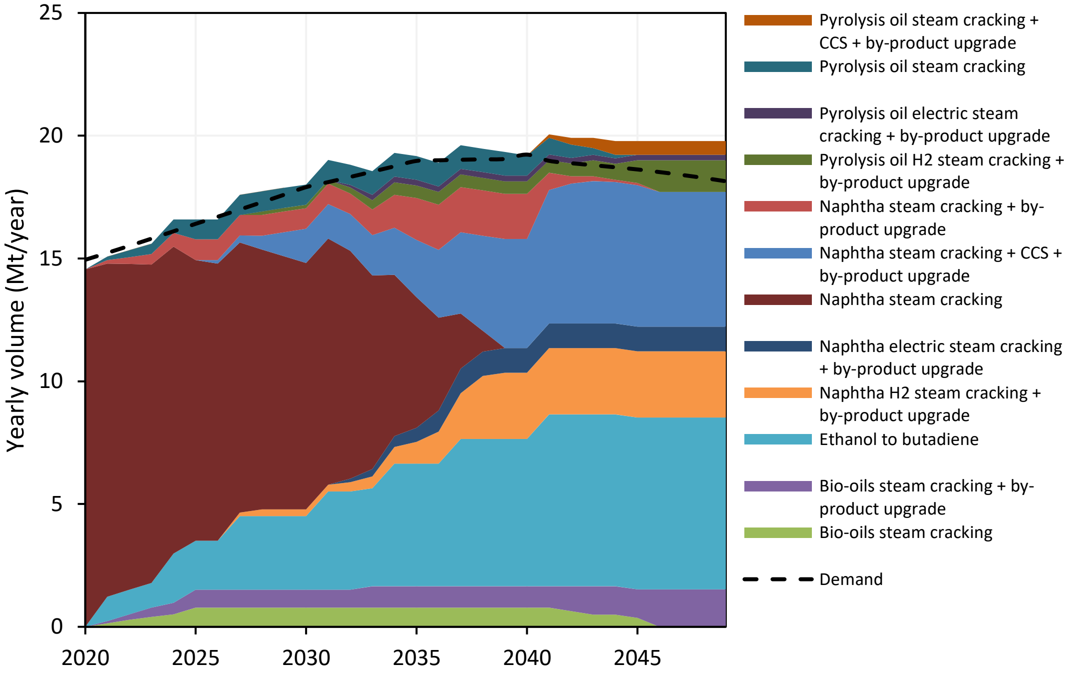

Fig. S42.

Technology over time for ethylene demand and volumes under LC-ME scenario

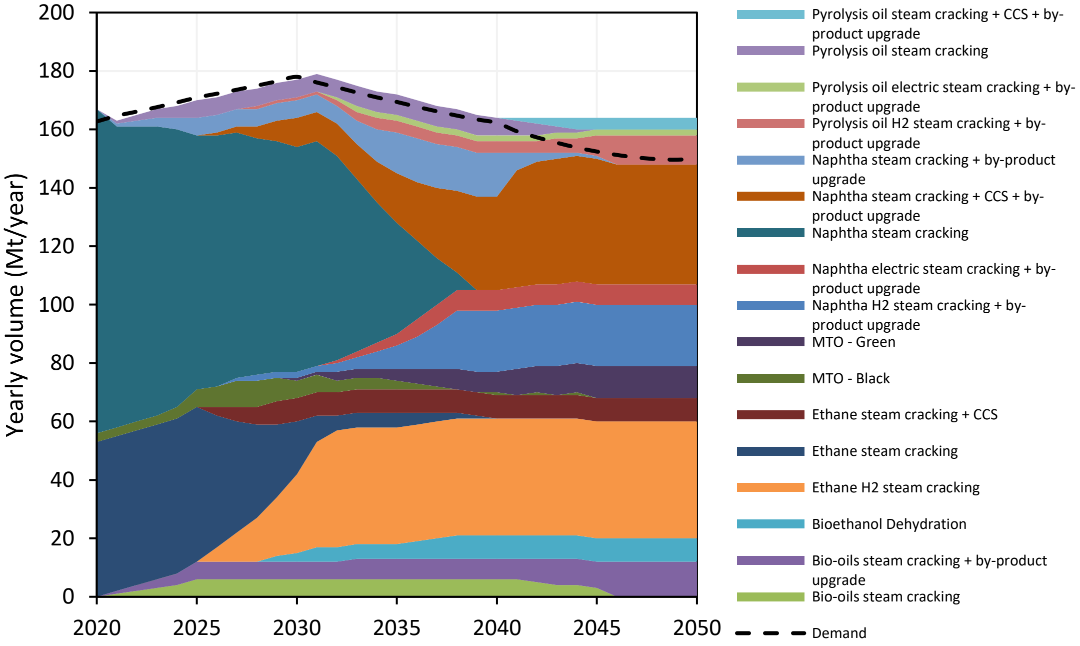

Fig. S43.
Technology over time for methanol demand and volumes under LC-ME scenario

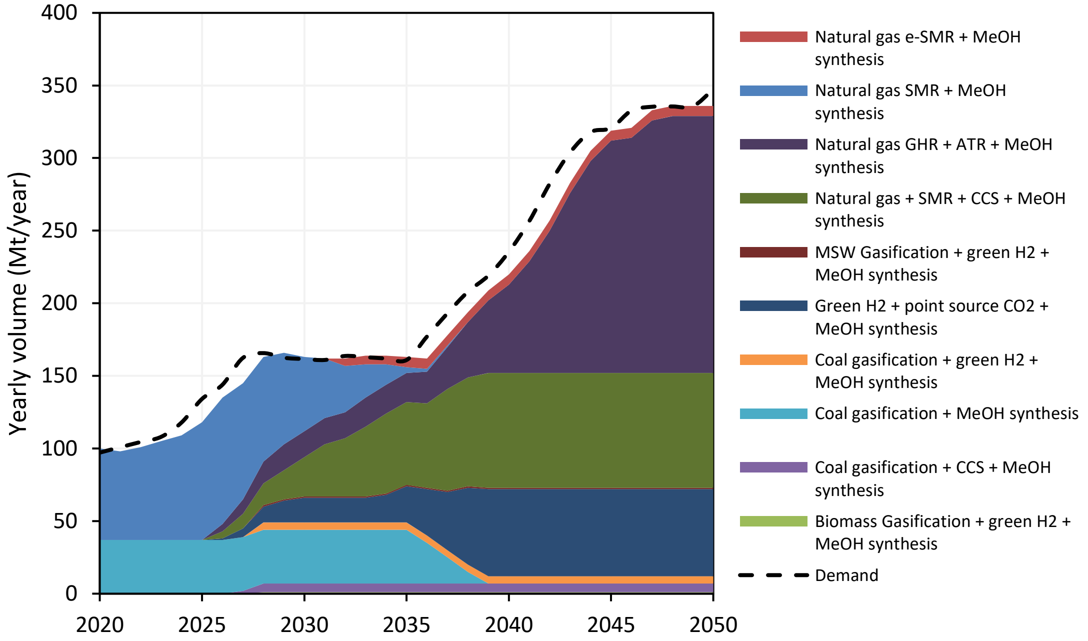

Fig. S44.

Technology over time for propylene demand and volumes under LC-ME scenario

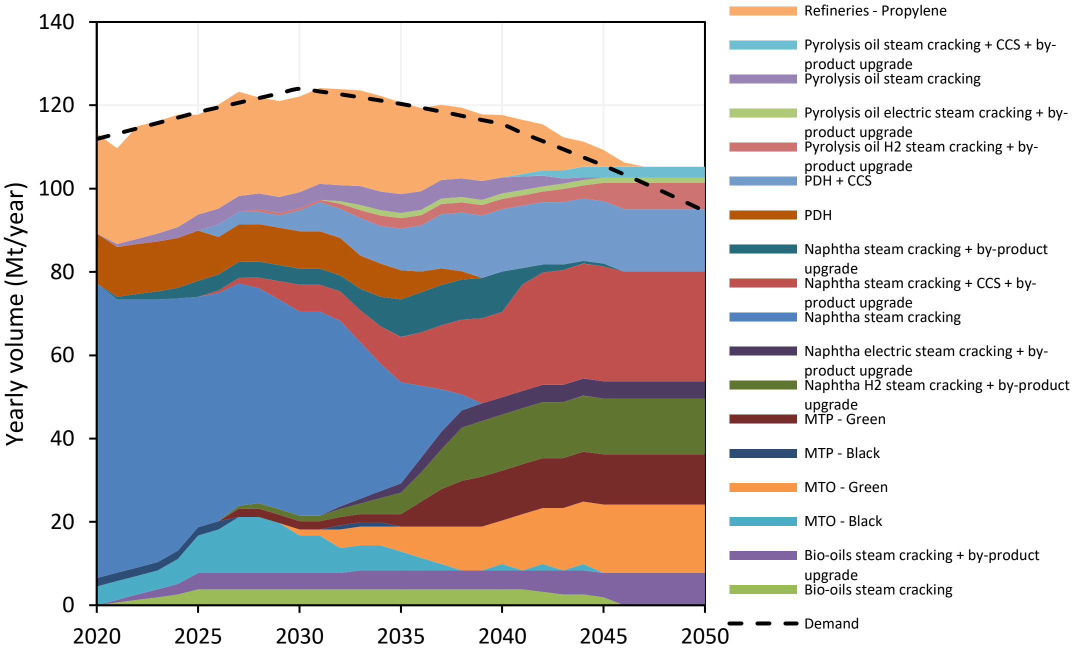

Fig. S45.
Technology over time for toluene demand and volumes under LC-ME scenario

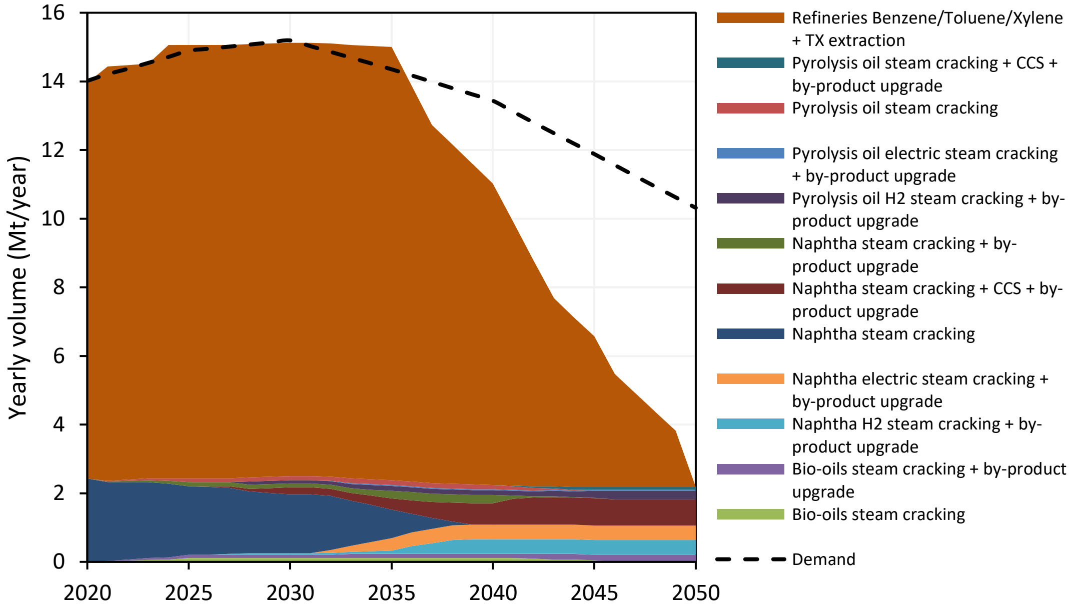

Fig. S46.
Technology over time for urea demand and volumes under LC-ME scenario

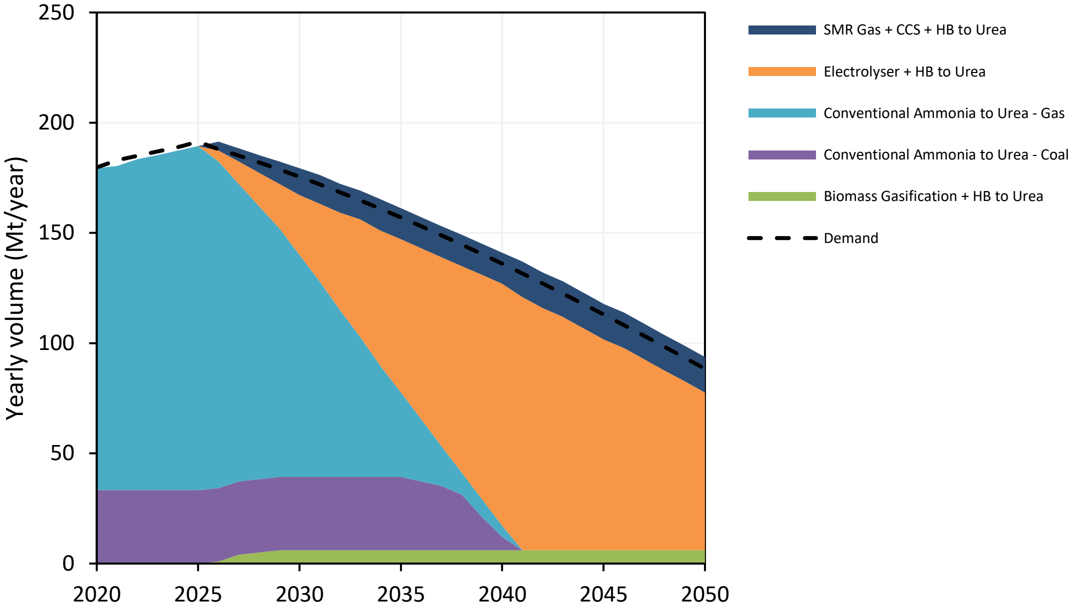

Fig. S47.
Technology over time for xylene demand and volumes under LC-ME scenario

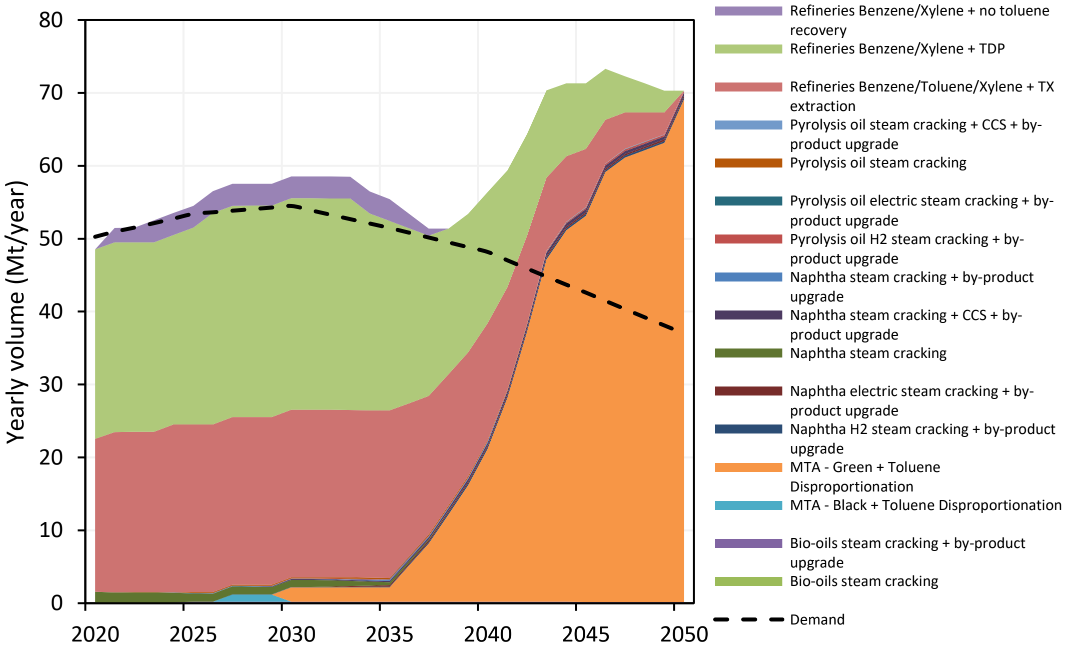

Fig. S48.
Technology over time for ammonia demand and volumes under HC-ME scenario

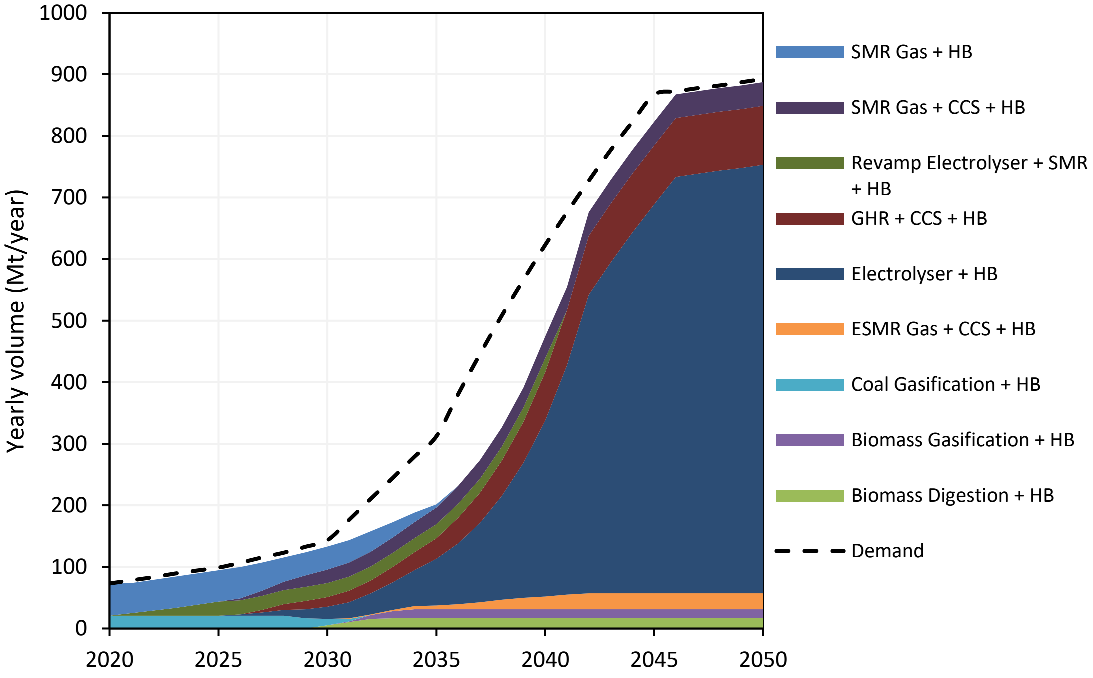

Fig. S49.
Technology over time for ammonium nitrate demand and volumes under HC-ME scenario

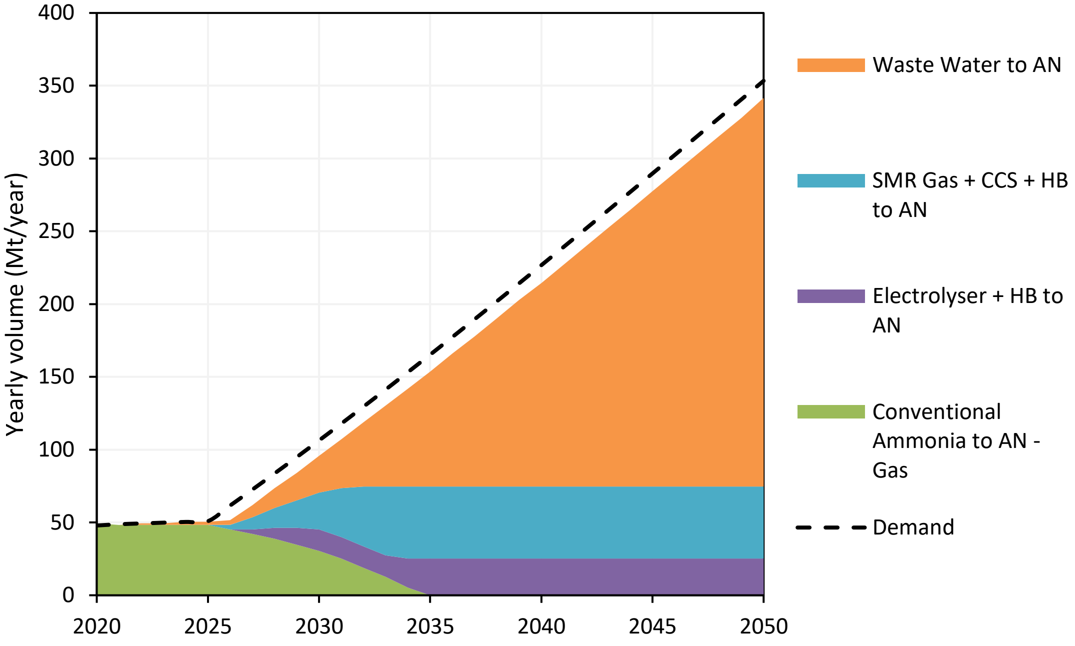

Fig. S50.
Technology over time for benzene demand and volumes under HC-ME scenario

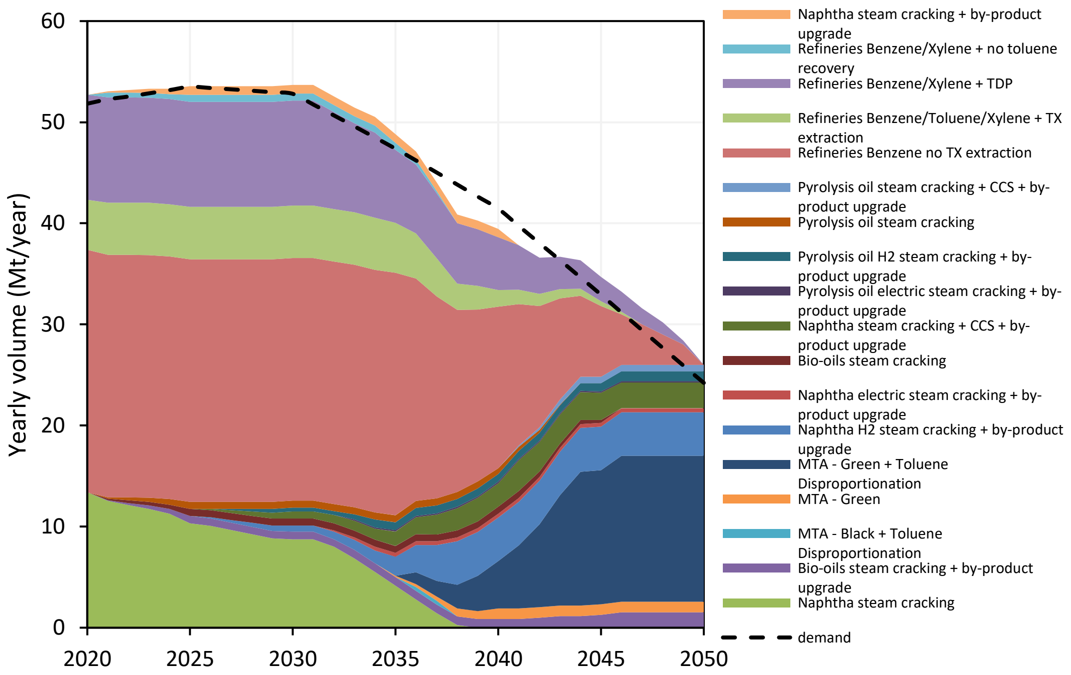

Fig. S51.
Technology over time for butadiene demand and volumes under HC-ME scenario

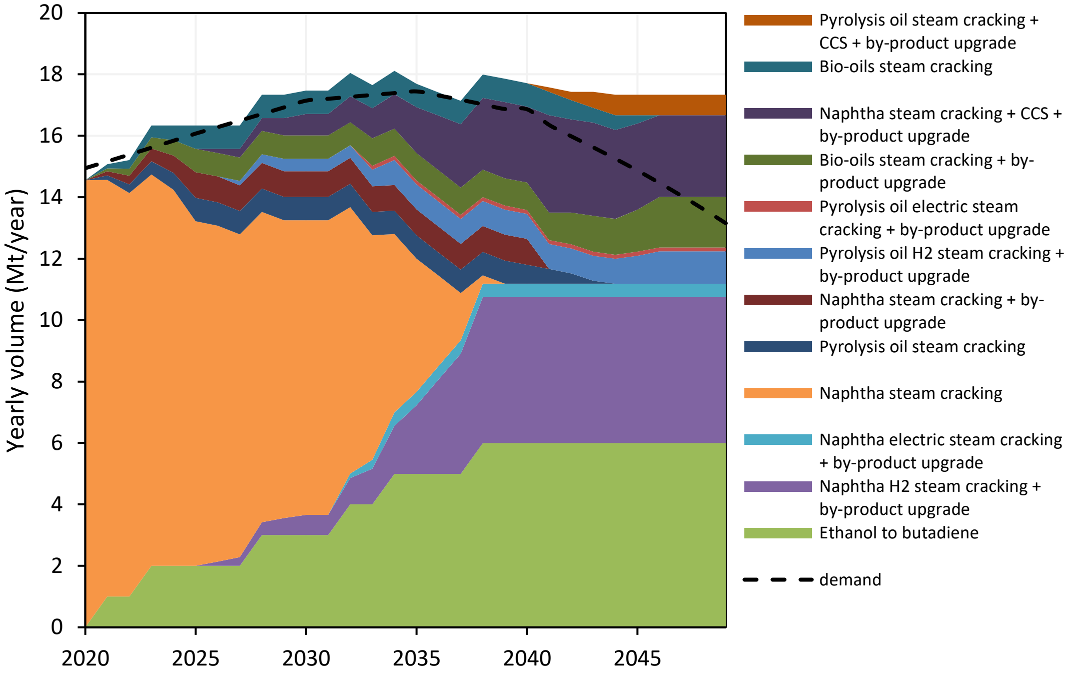

Fig. S52.
Technology over time for ethylene demand and volumes under HC-ME scenario

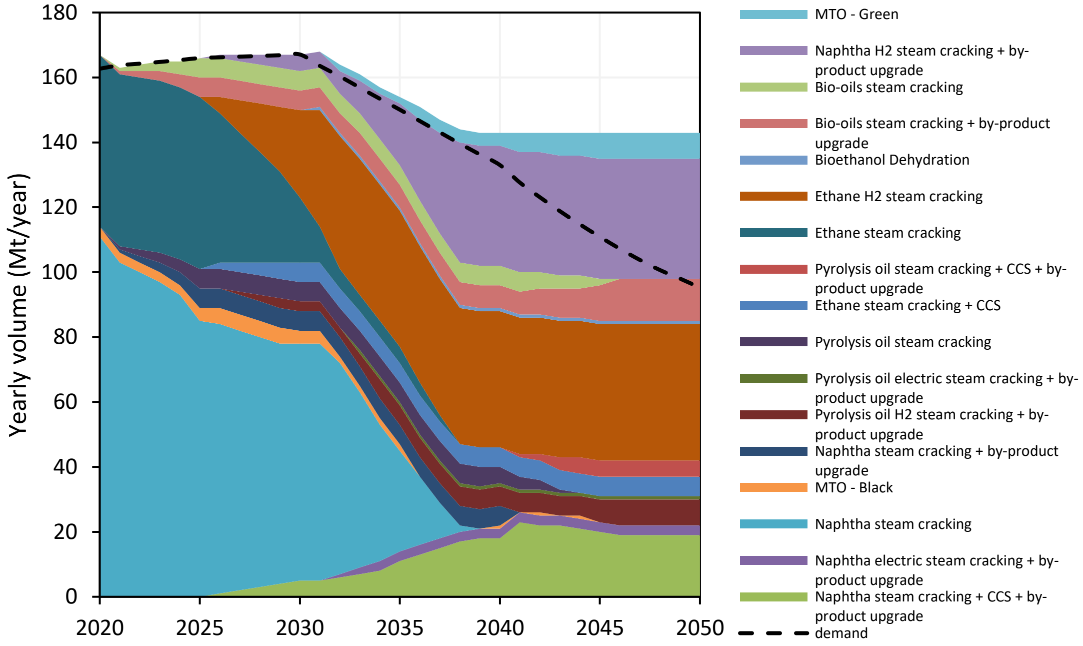

Fig. S53.
Technology over time for methanol demand and volumes under HC-ME scenario

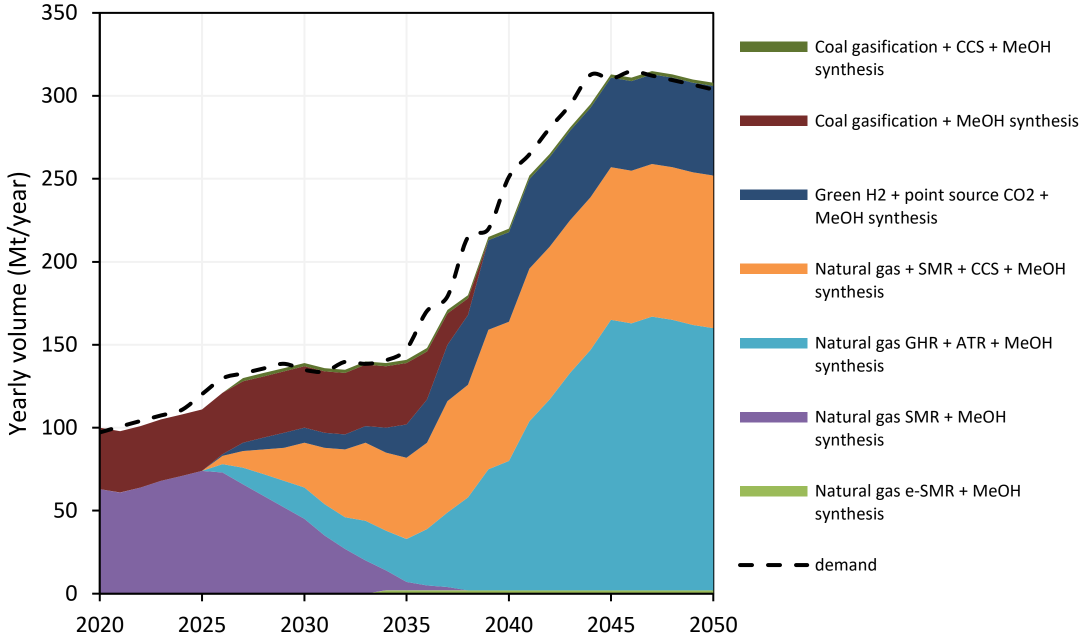

Fig. S54.
Technology over time for propylene demand and volumes under HC-ME scenario

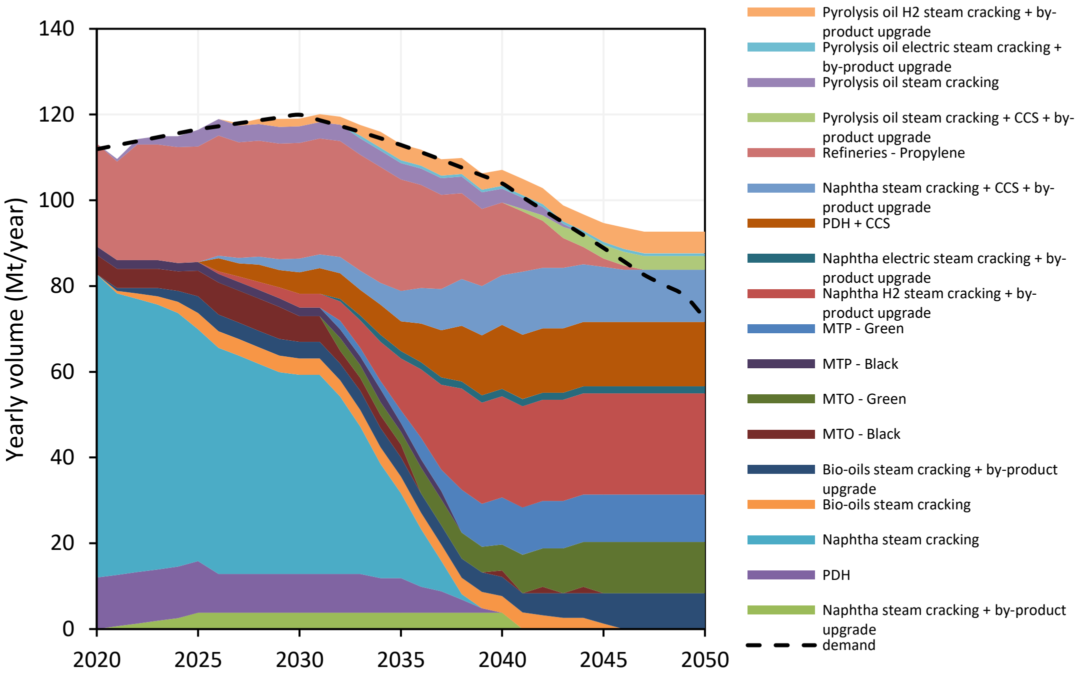

Fig. S55.
Technology over time for toluene demand and volumes under HC-ME scenario

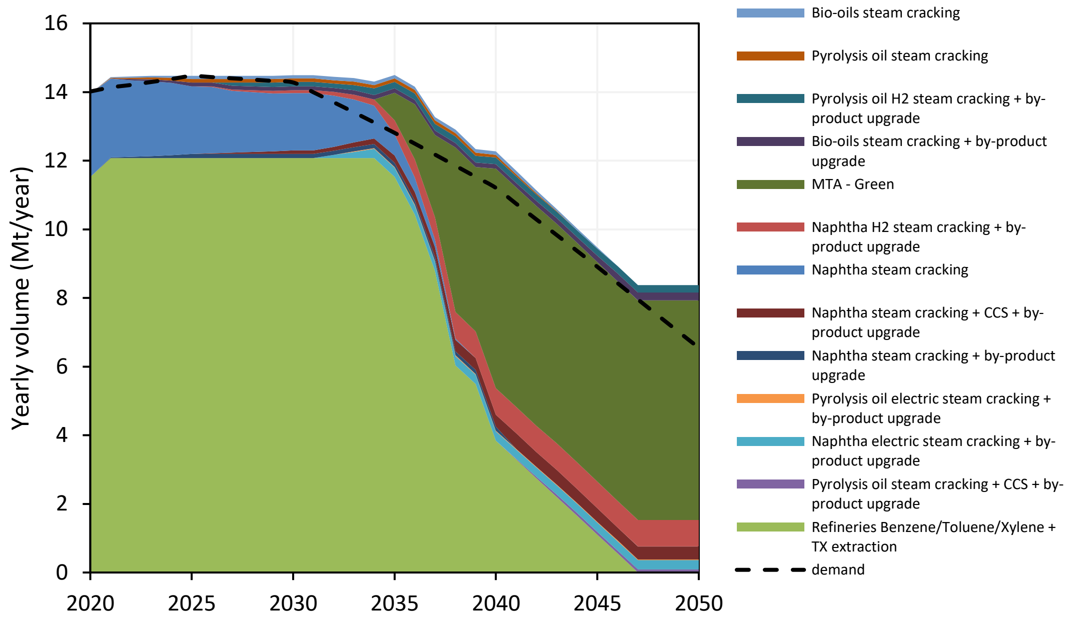

Fig. S56.
Technology over time for urea demand and volumes under HC-ME scenario

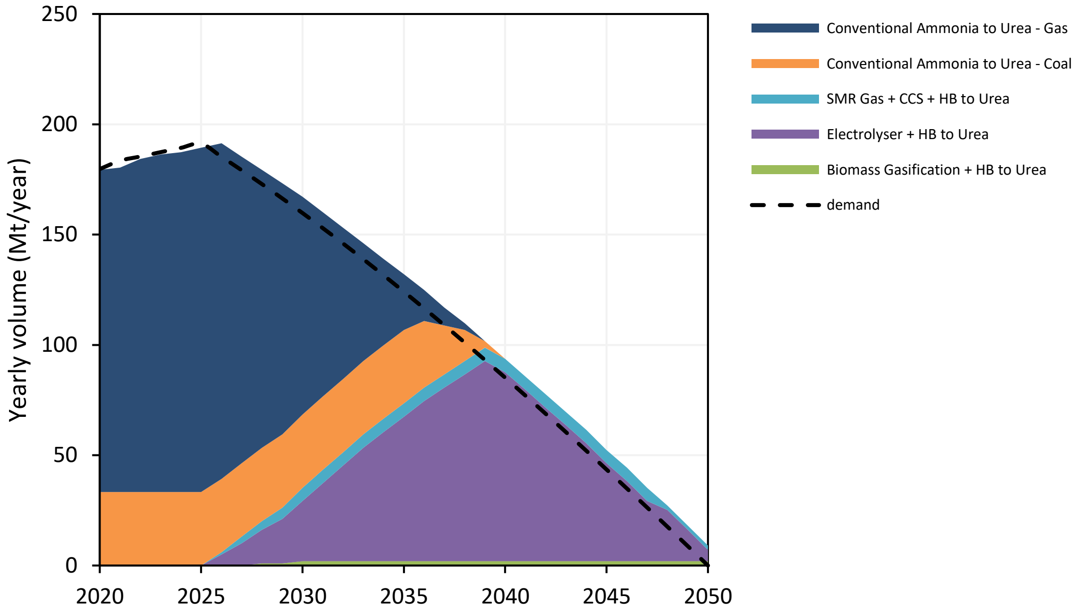

Fig. S57.
Technology over time for xylene demand and volumes under HC-ME scenario

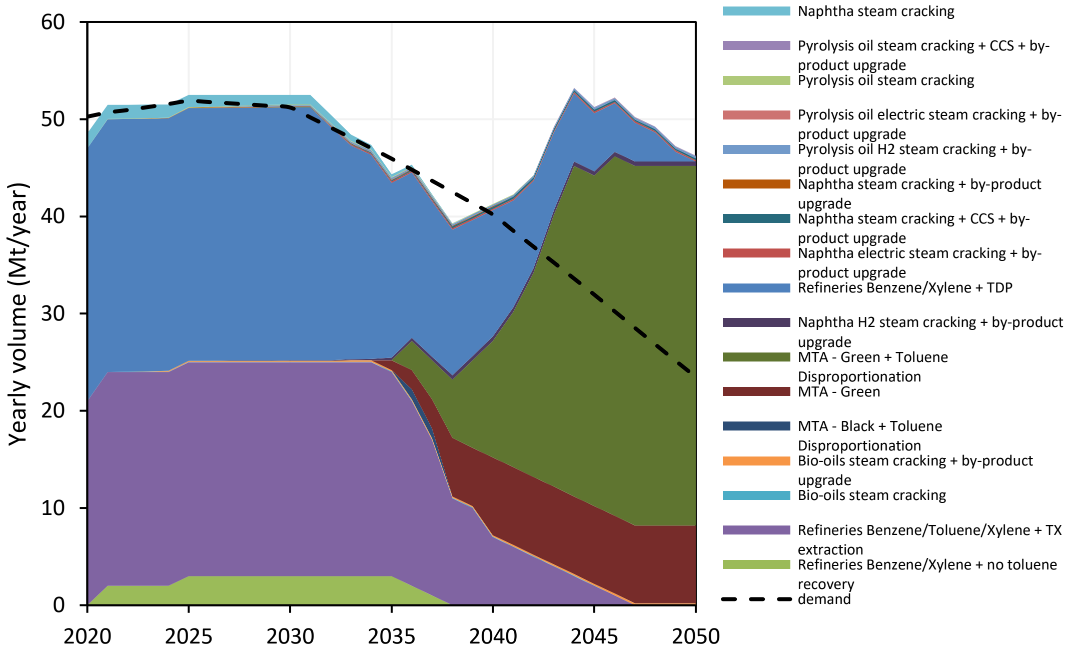

Fig. S58.
Technology over time for ammonia demand and volumes under BDEM – BAU scenario

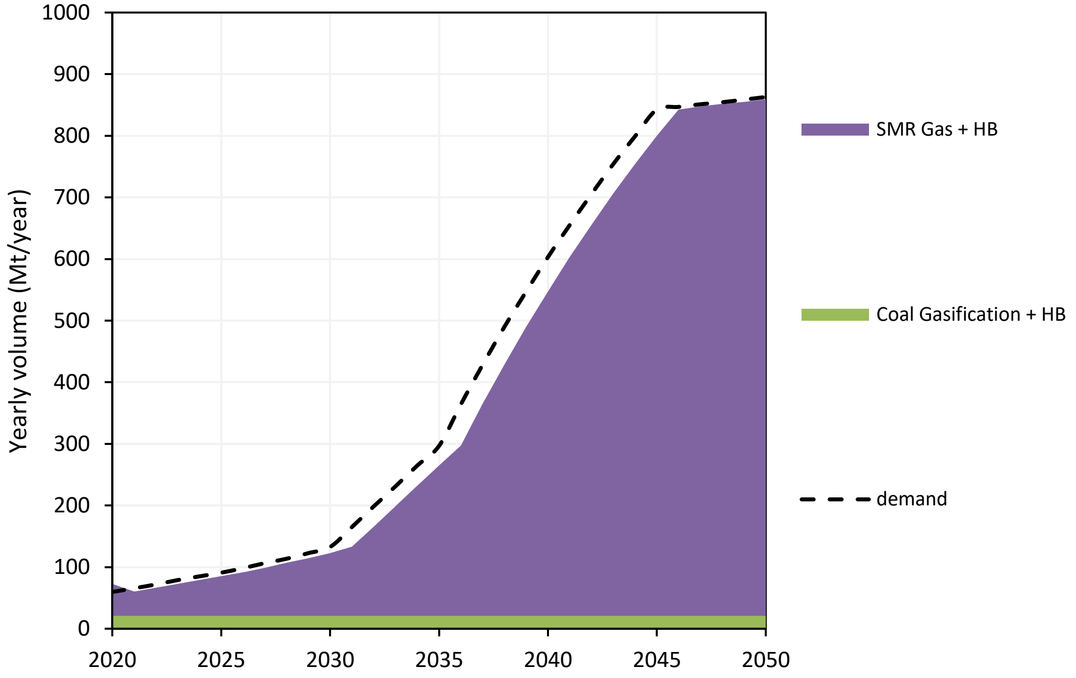

Fig. S59.
Technology over time for ammonium nitrate demand and volumes under BDEM – BAU scenario

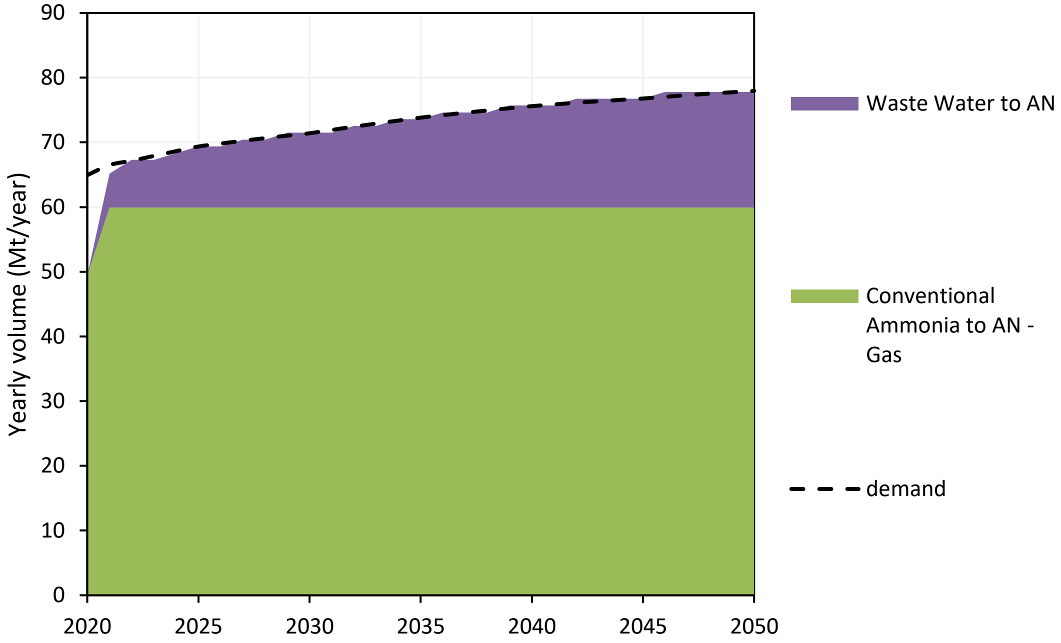

Fig. S60.
Technology over time for benzene demand and volumes under BDEM – BAU scenario

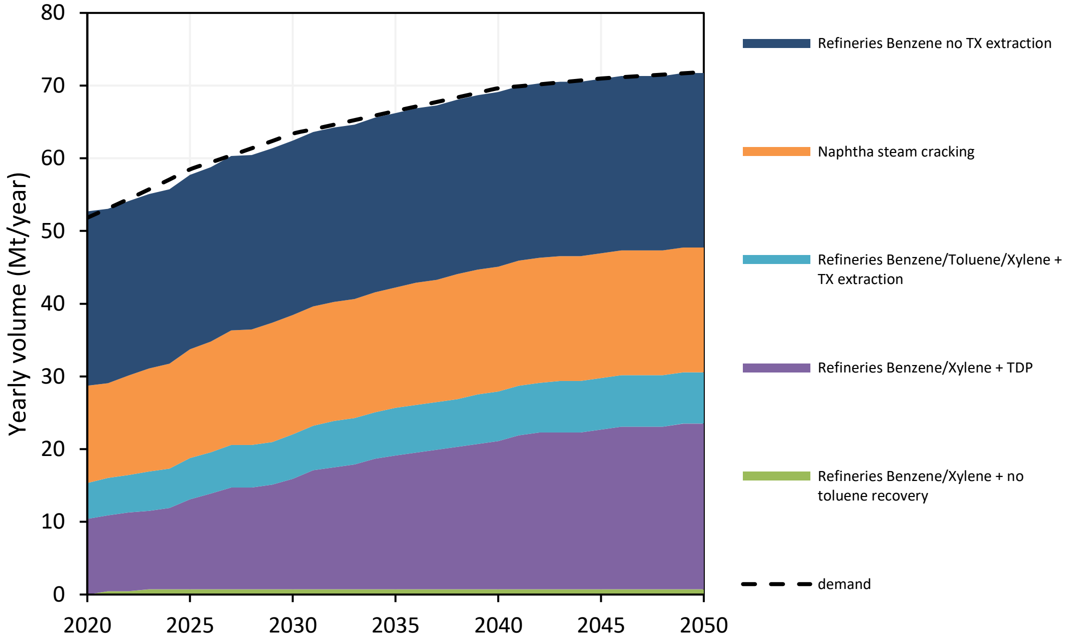

Fig. S61.
Technology over time for butadiene demand and volumes under BDEM – BAU scenario

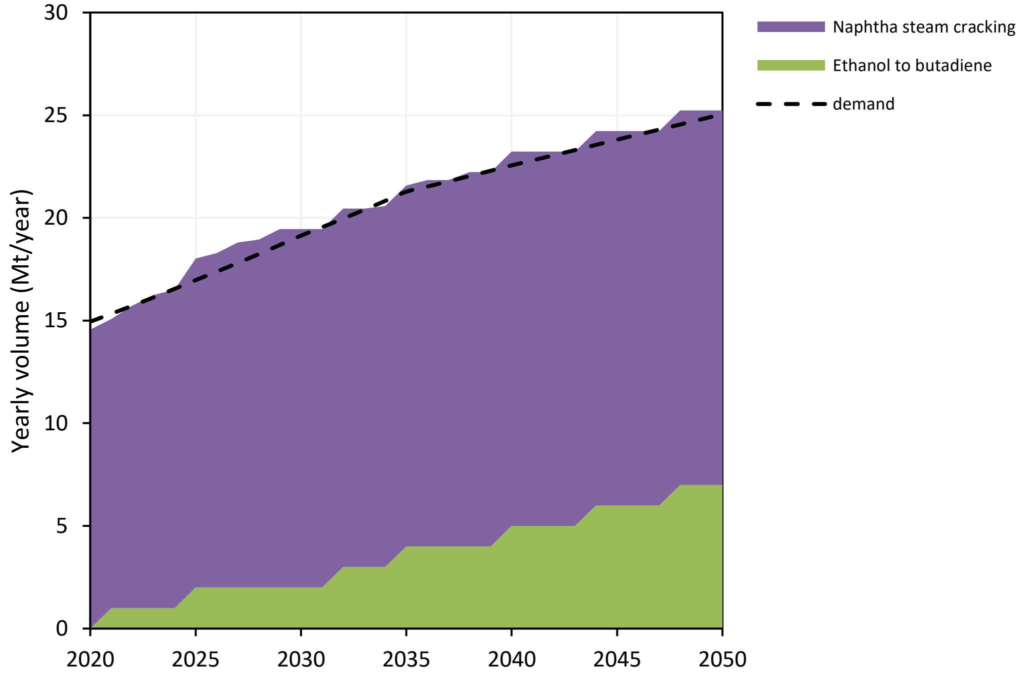

Fig. S62.
Technology over time for ethylene demand and volumes under BDEM – BAU scenario

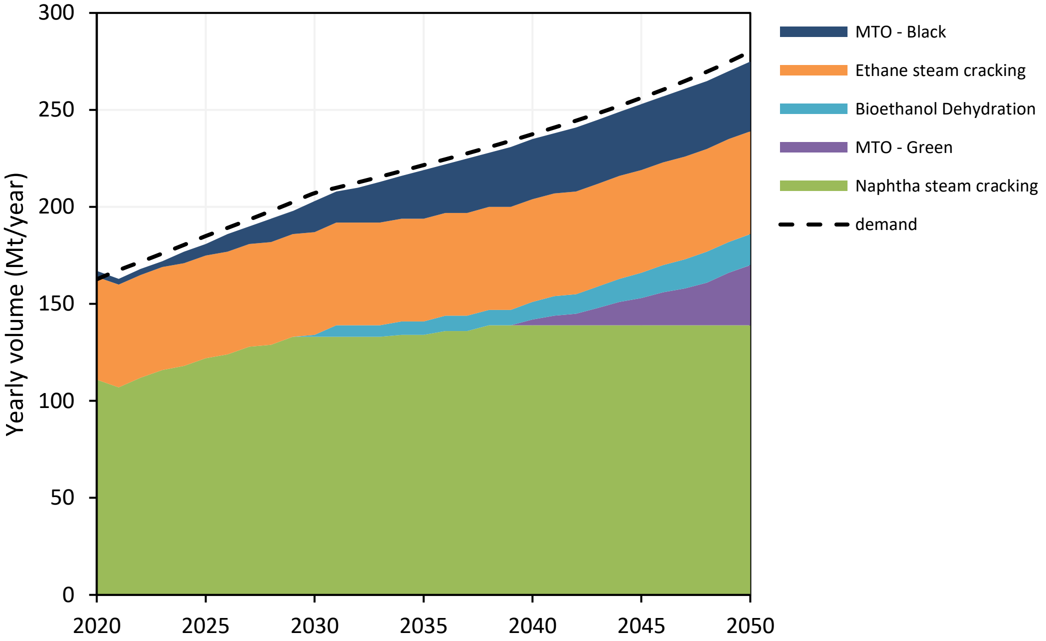

Fig. S63.
Technology over time for methanol demand and volumes under BDEM – BAU scenario

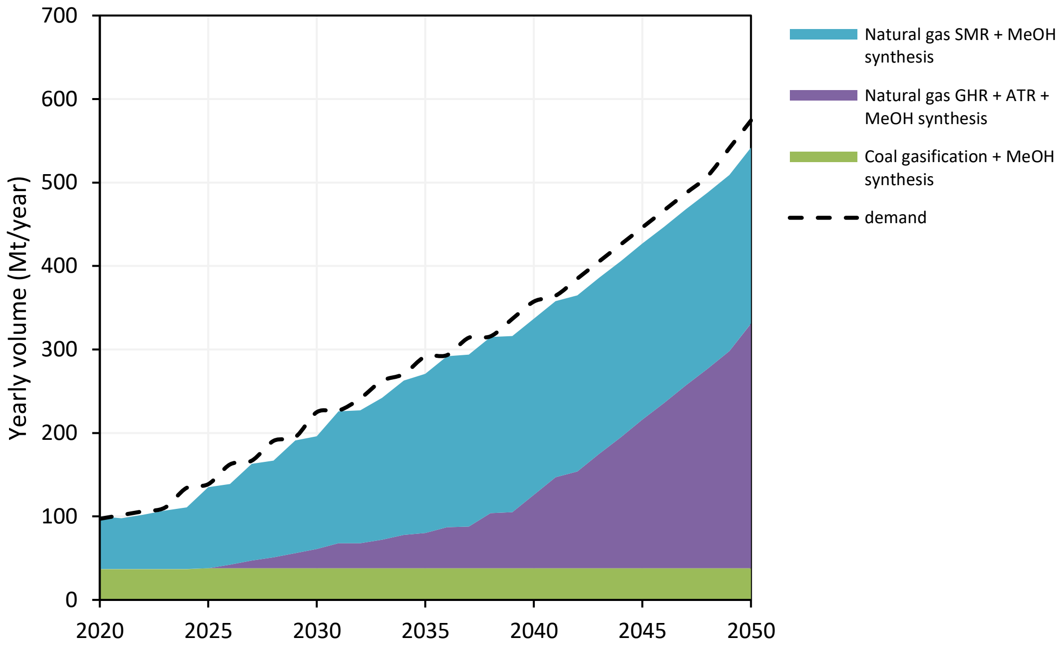

Fig. S64.
Technology over time for propylene demand and volumes under BDEM – BAU scenario

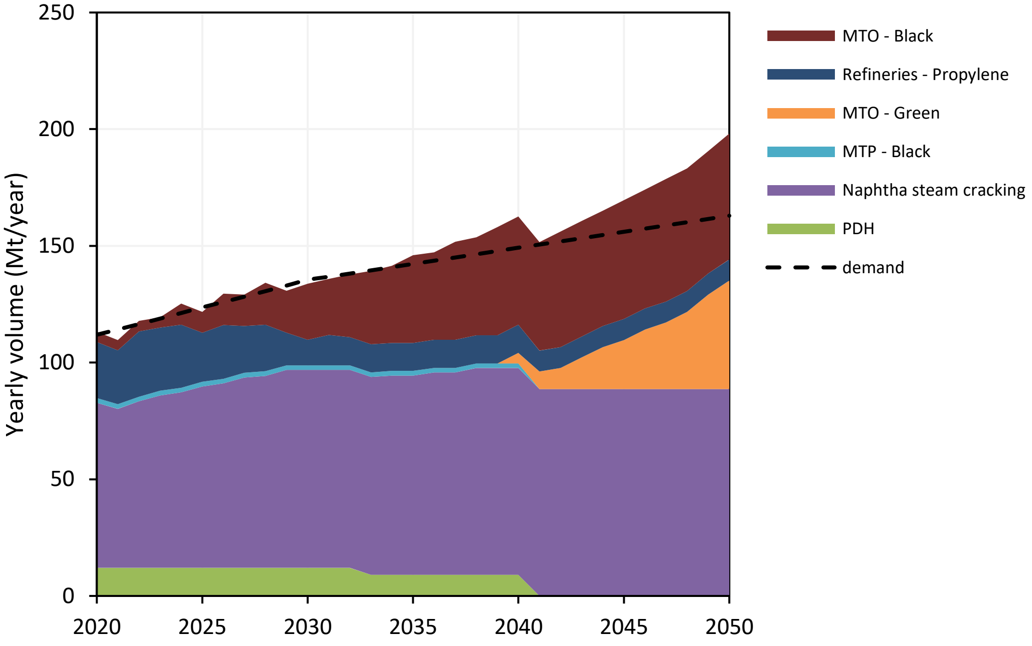

Fig. S65.
Technology over time for toluene demand and volumes under BDEM – BAU scenario

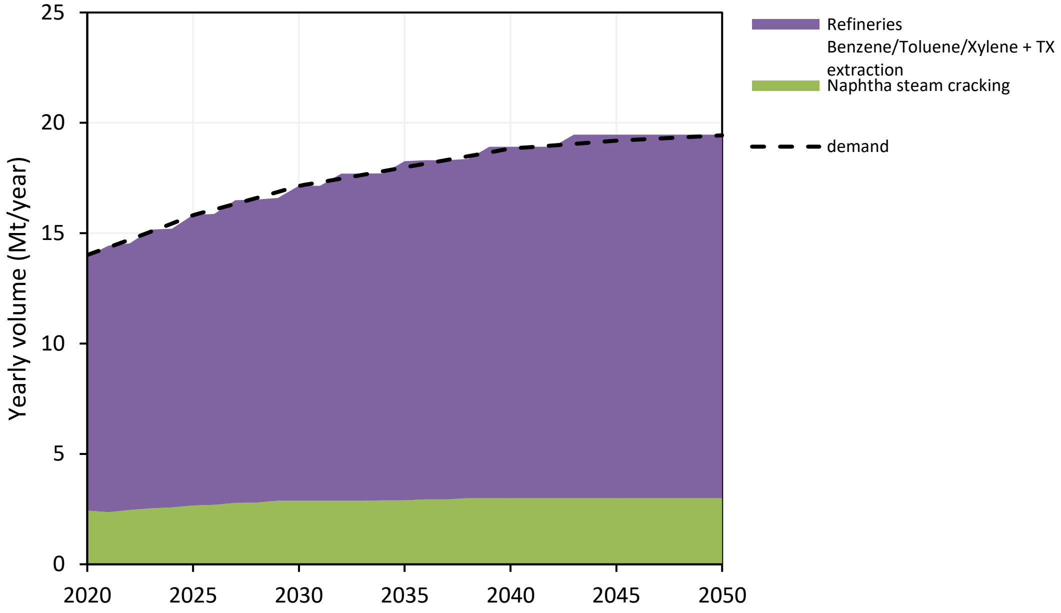

Fig. S66.
Technology over time for urea demand and volumes under BDEM – BAU scenario

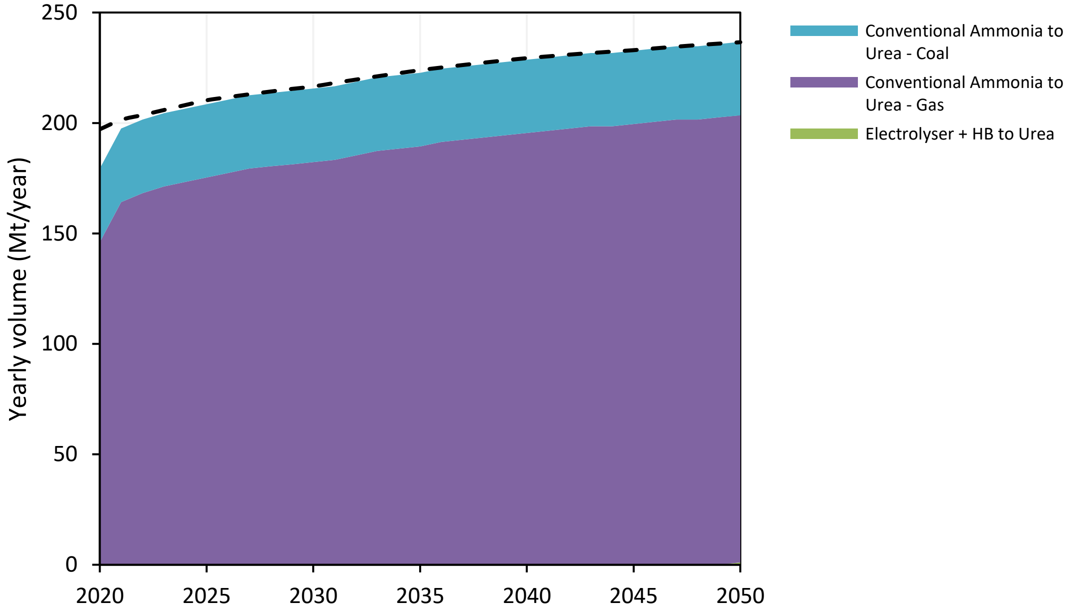

Fig. S67.
Technology over time for xylene demand and volumes under BDEM – BAU scenario

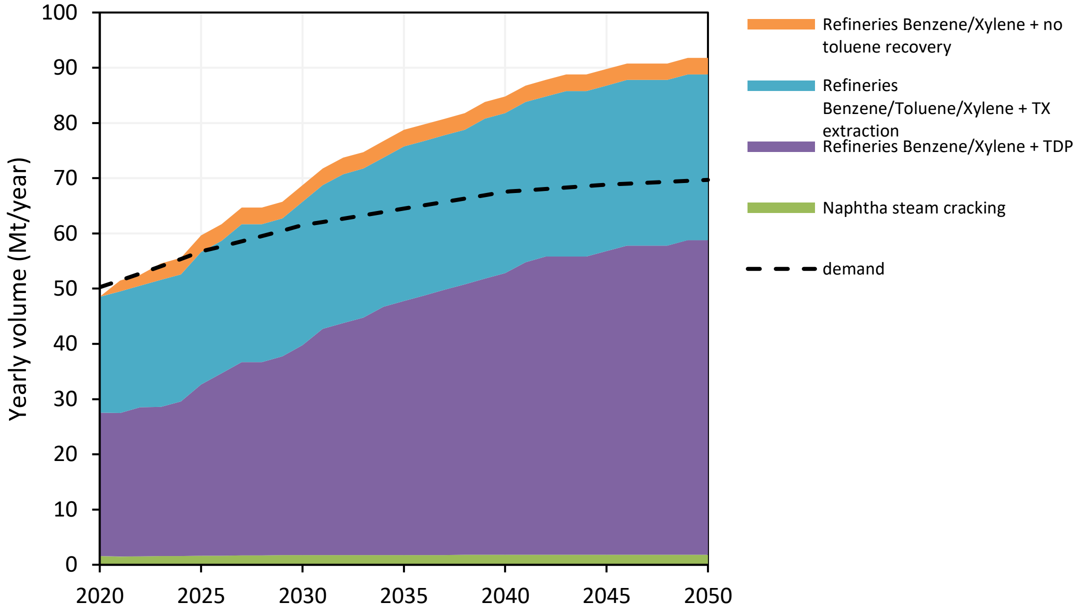

Fig. S68.
Technology over time for ammonia demand and volumes under LC-NFAX scenario

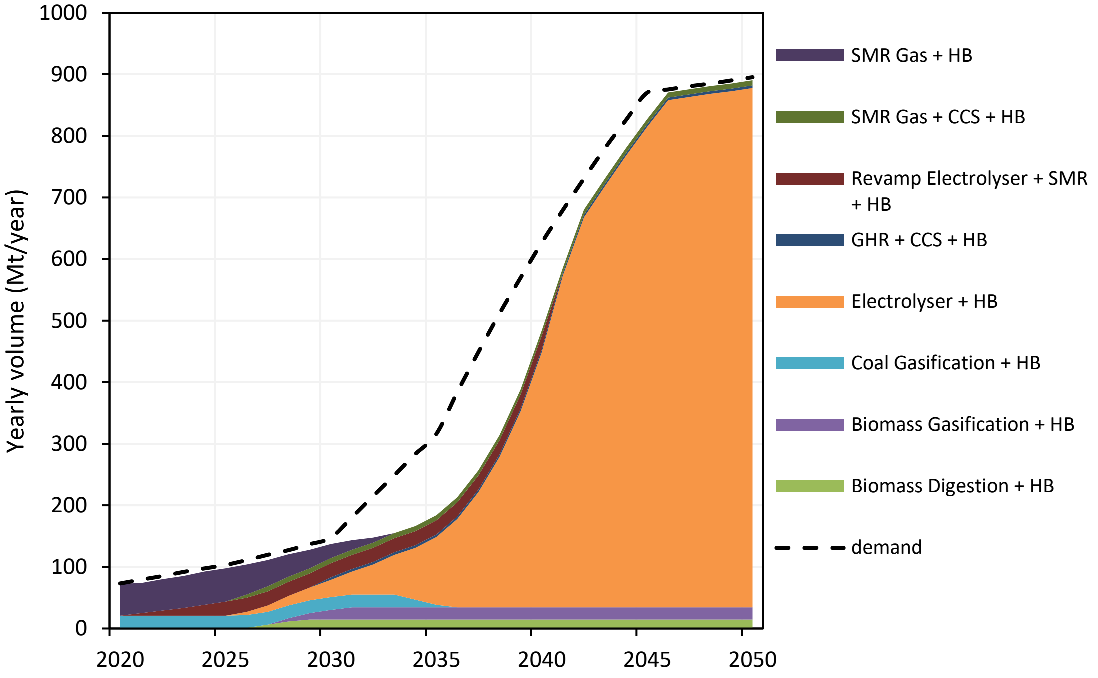

Fig. S69.
Technology over time for ammonium nitrate demand and volumes under LC-NFAX scenario

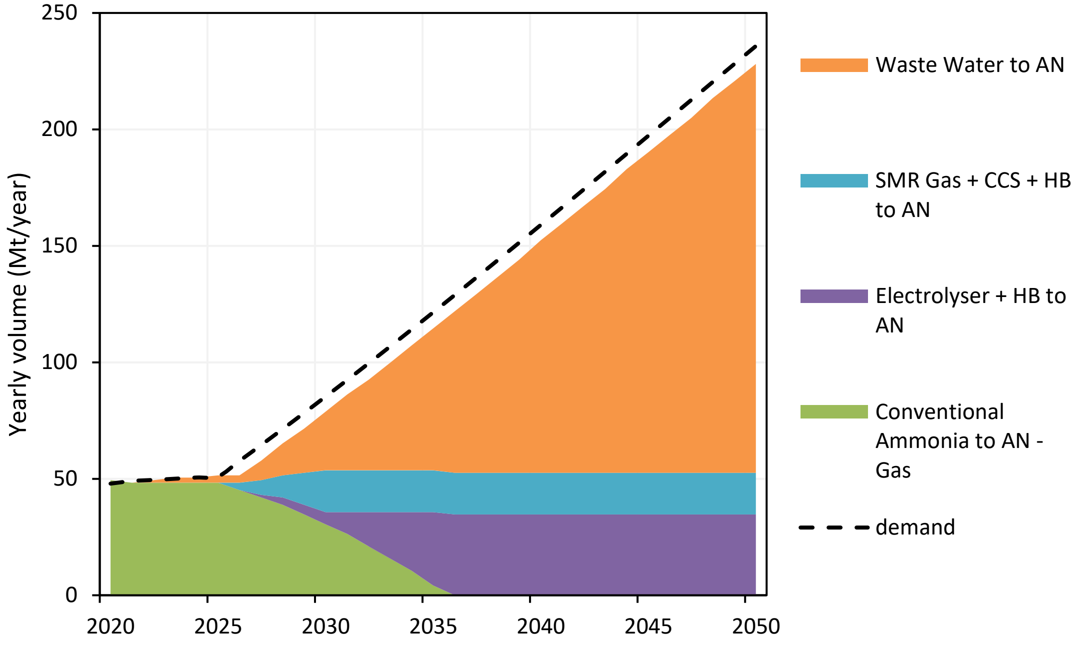

Fig. S70.
Technology over time for benzene demand and volumes under LC-NFAX scenario

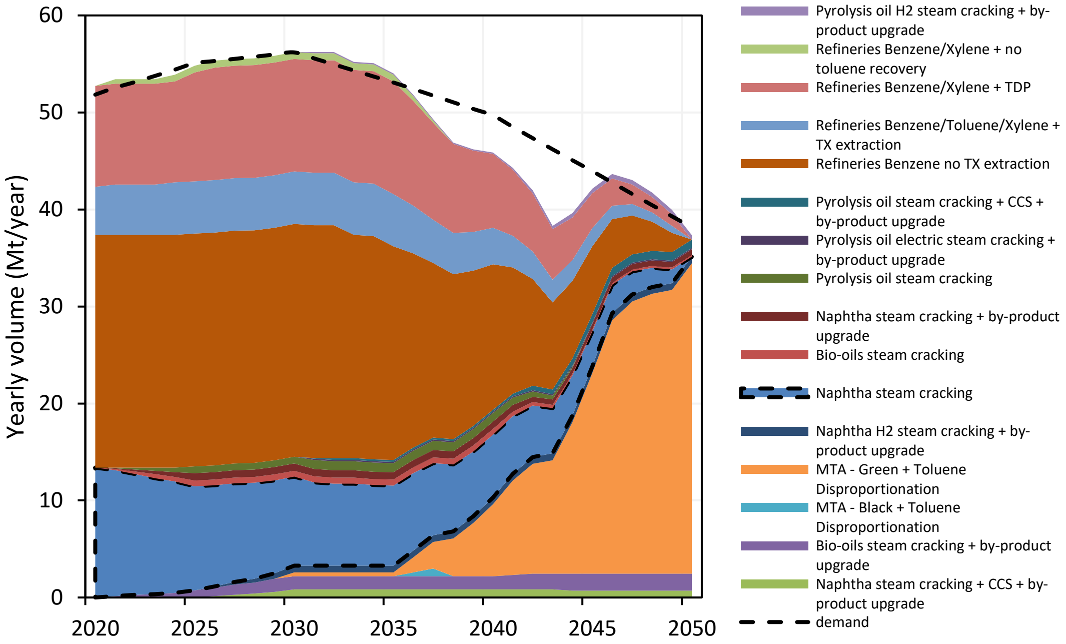

Fig. S71.

Technology over time for butadiene demand and volumes under LC-NFAX scenario

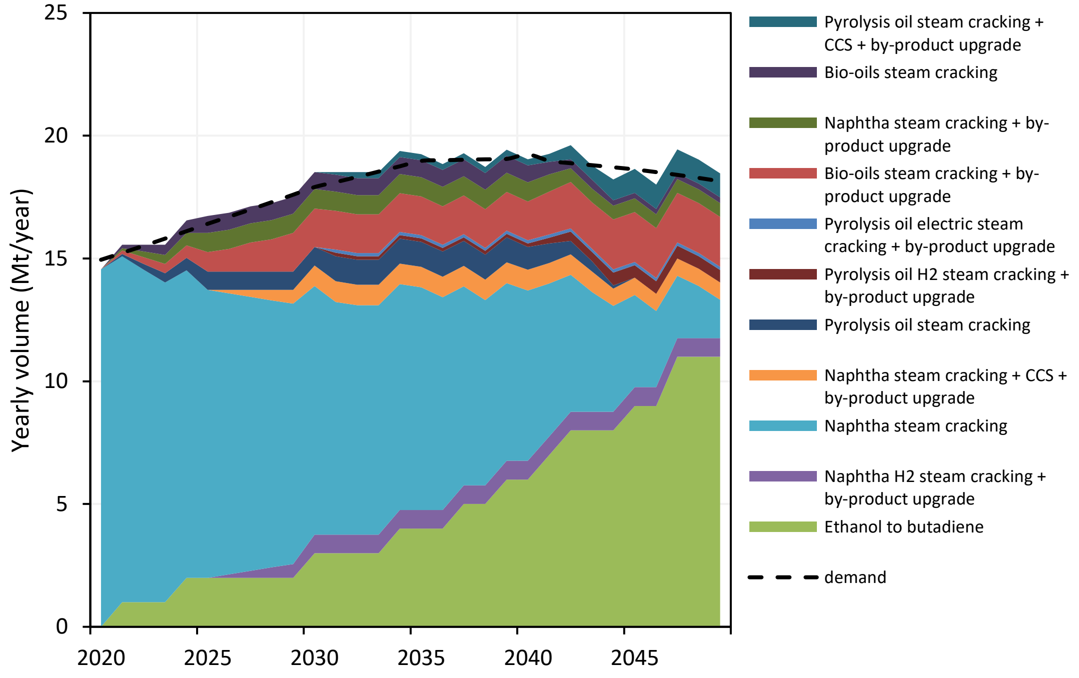

Fig. S72.
Technology over time for ethylene demand and volumes under LC-NFAX scenario

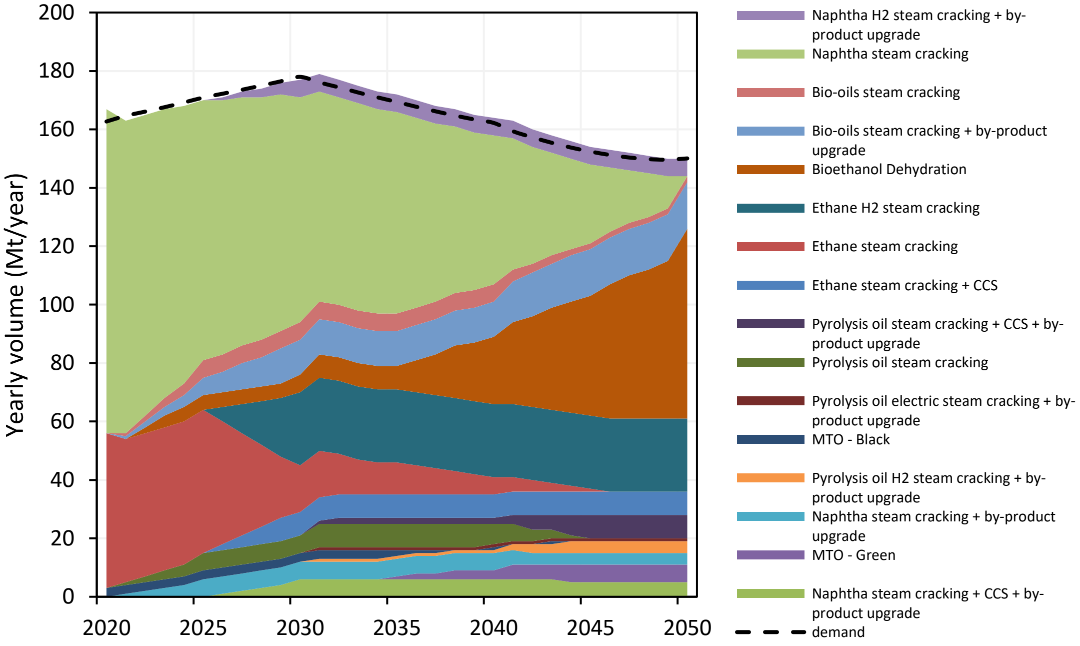

Fig. S73.
Technology over time for methanol demand and volumes under LC-NFAX scenario

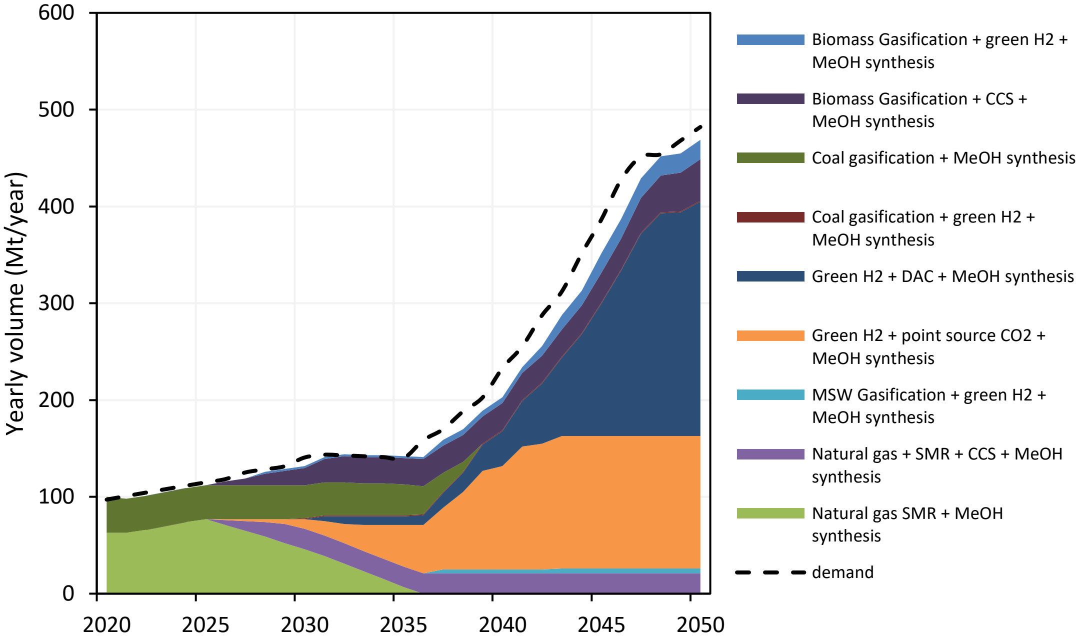

Fig. S74.
Technology over time for propylene demand and volumes under LC-NFAX scenario

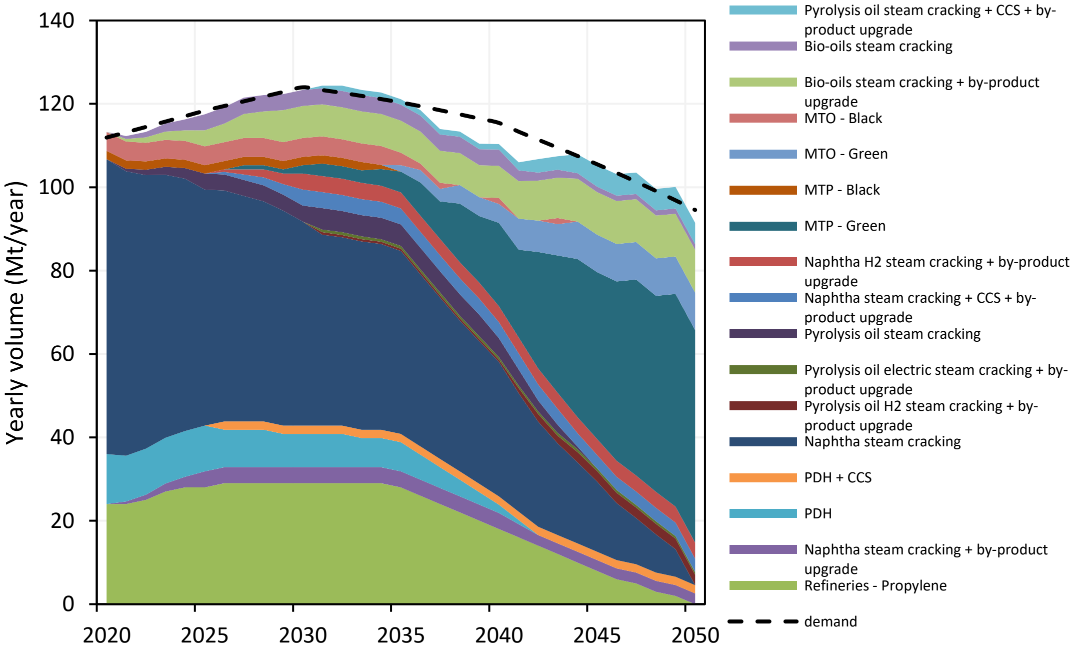

Fig. S75.
Technology over time for toluene demand and volumes under LC-NFAX scenario

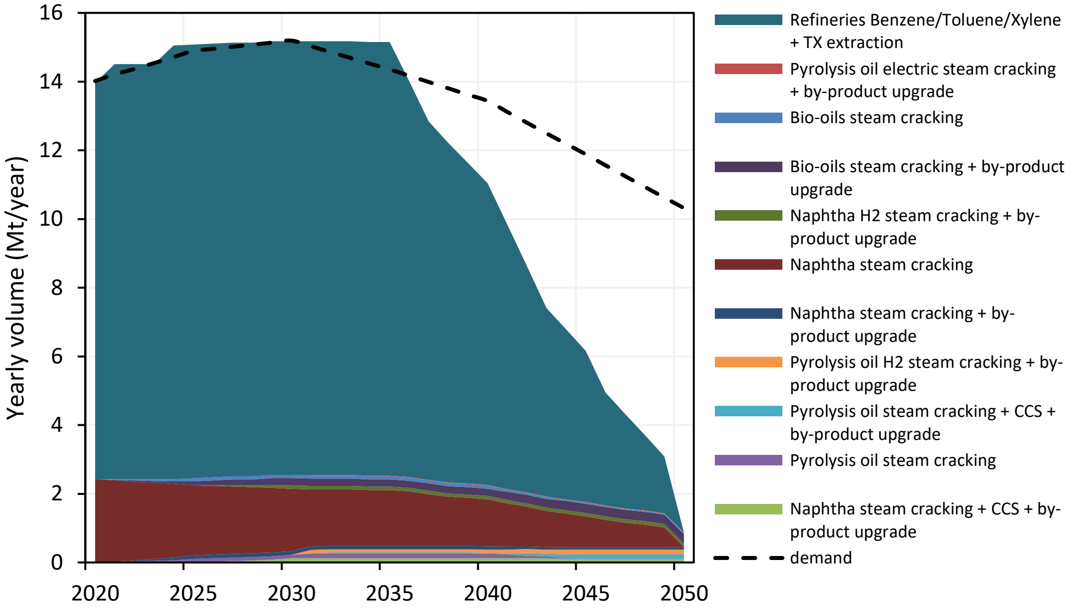

Fig. S76.
Technology over time for urea demand and volumes under LC-NFAX scenario

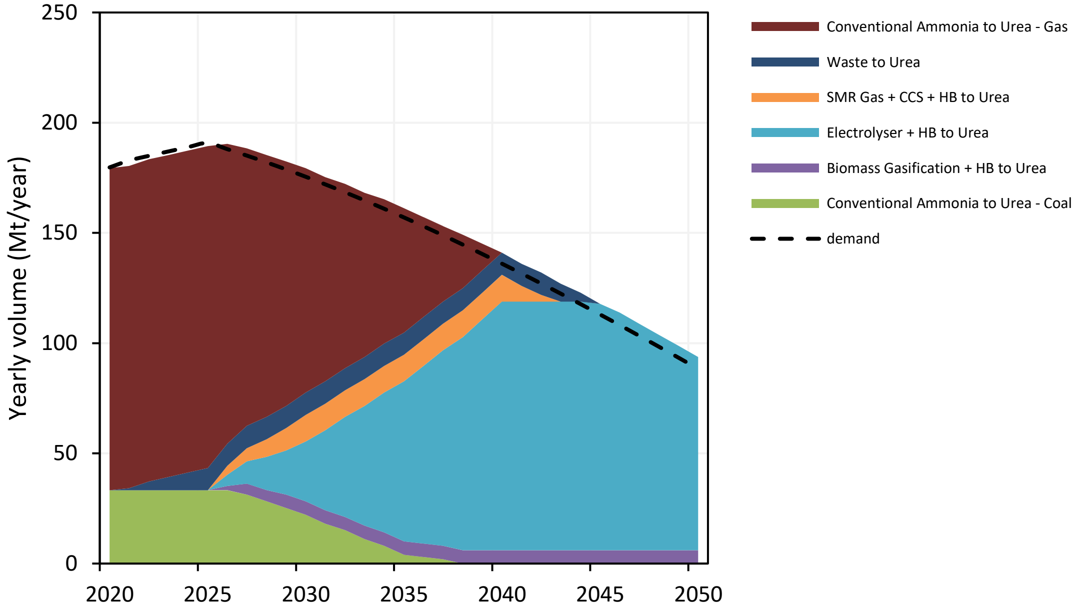

Fig. S77.
Technology over time for xylene demand and volumes under LC-NFAX scenario

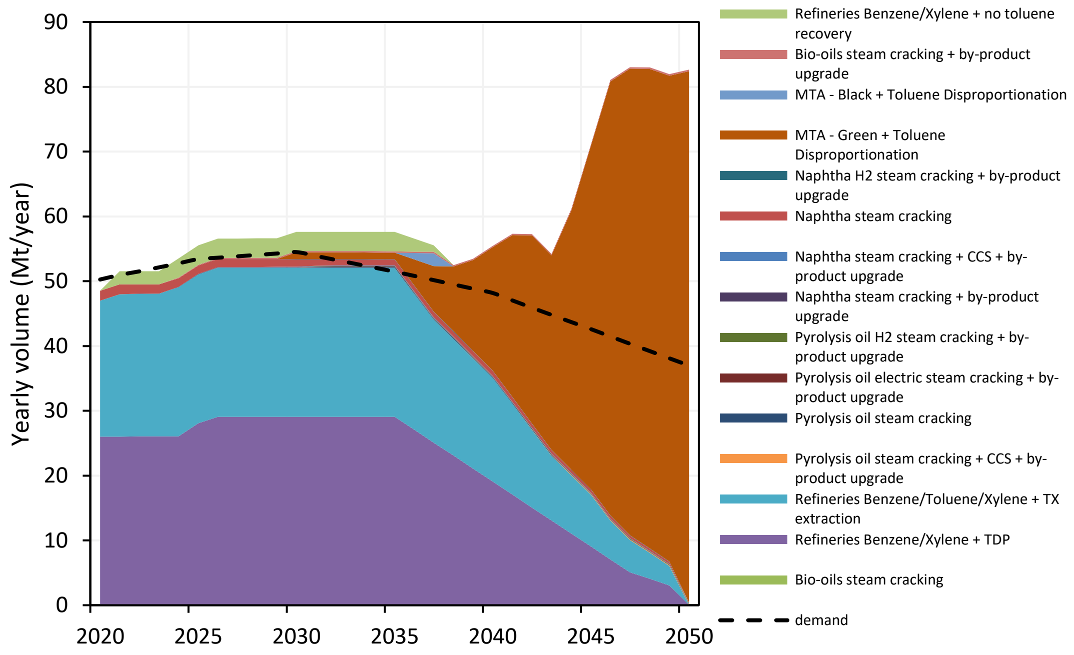

Fig. S78.
Technology over time for ammonia demand and volumes under HC-NFAX scenario

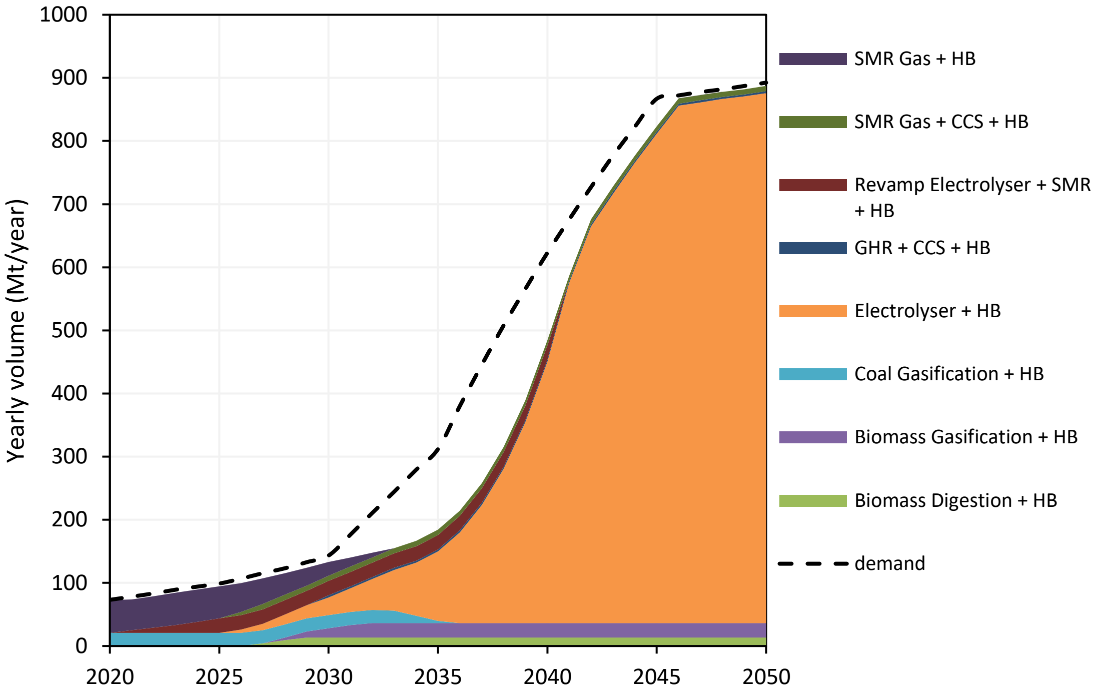

Fig. S79.
Technology over time for ammonium nitrate demand and volumes under HC-NFAX scenario

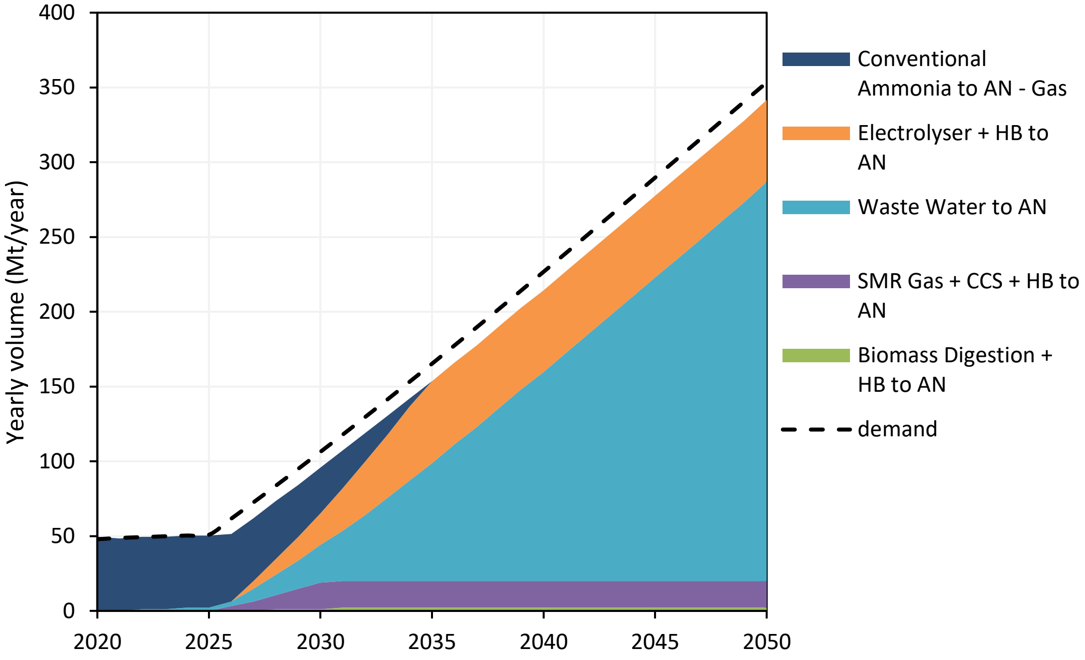

Fig. S80.

Technology over time for benzene demand and volumes under HC-NFAX scenario

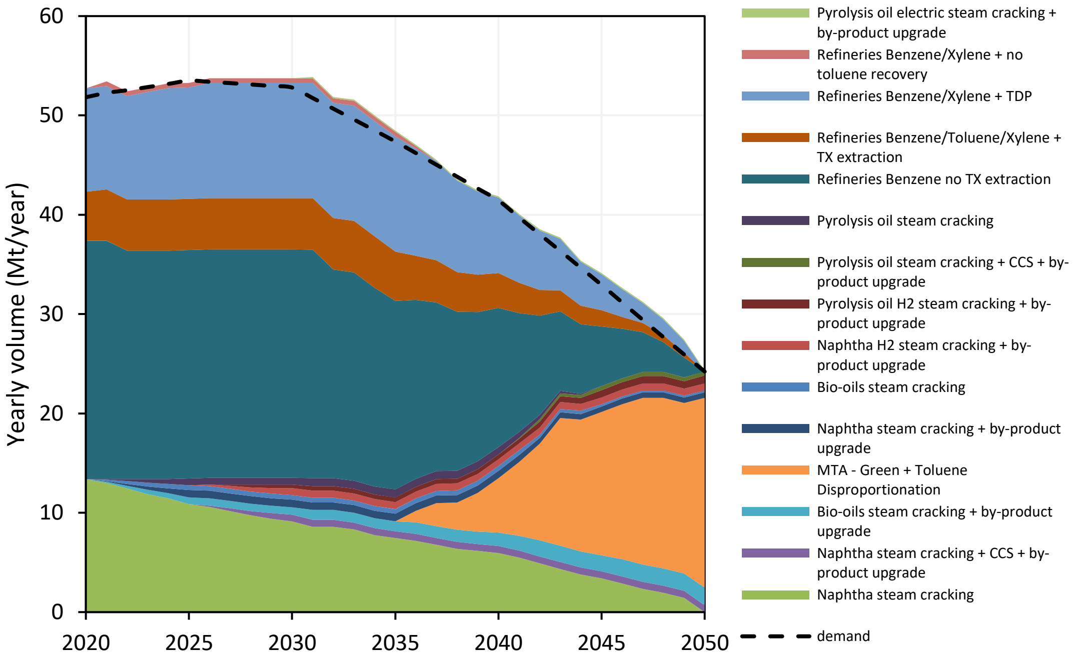

Fig. S81.
Technology over time for butadiene demand and volumes under HC-NFAX scenario

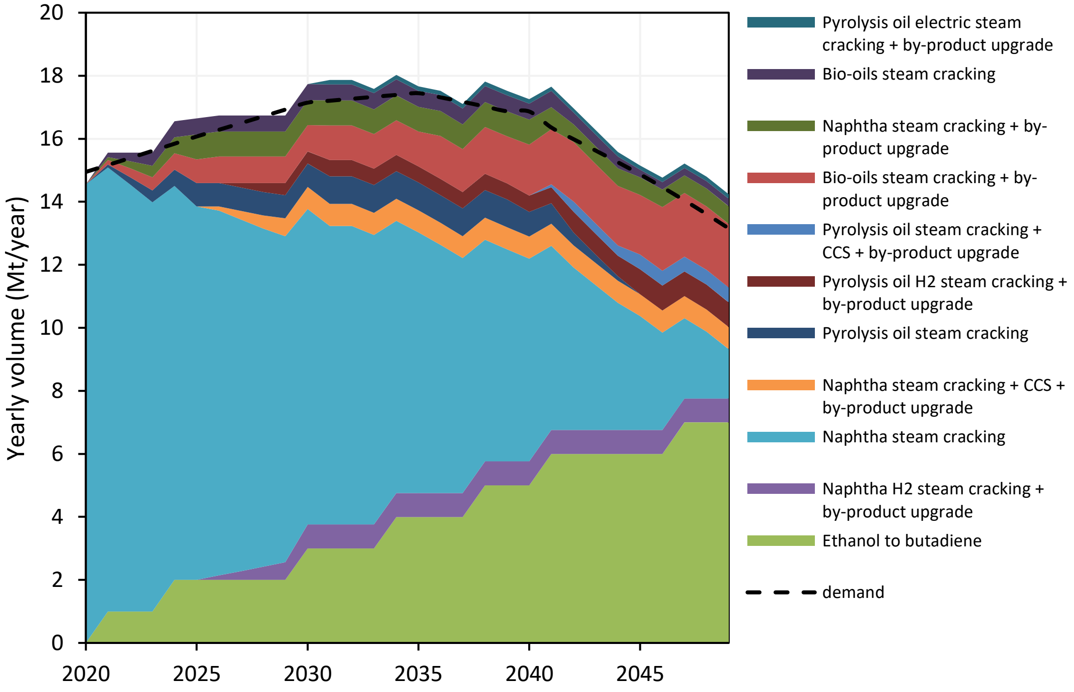

Fig. S82.
Technology over time for ethylene demand and volumes under HC-NFAX scenario

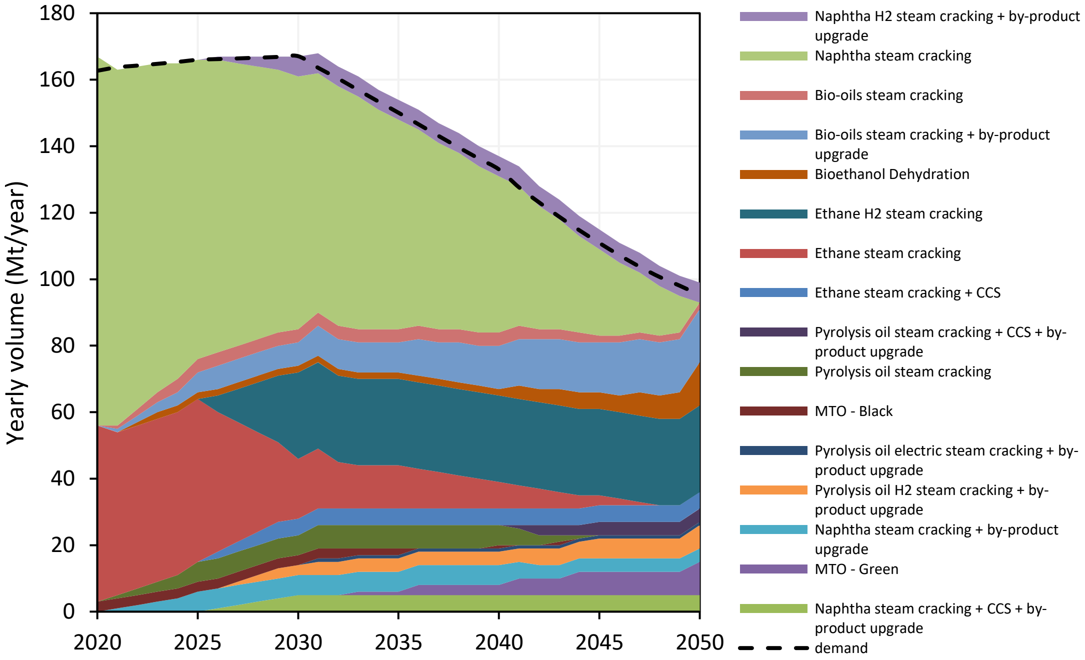

Fig. S83.
Technology over time for methanol demand and volumes under HC-NFAX scenario

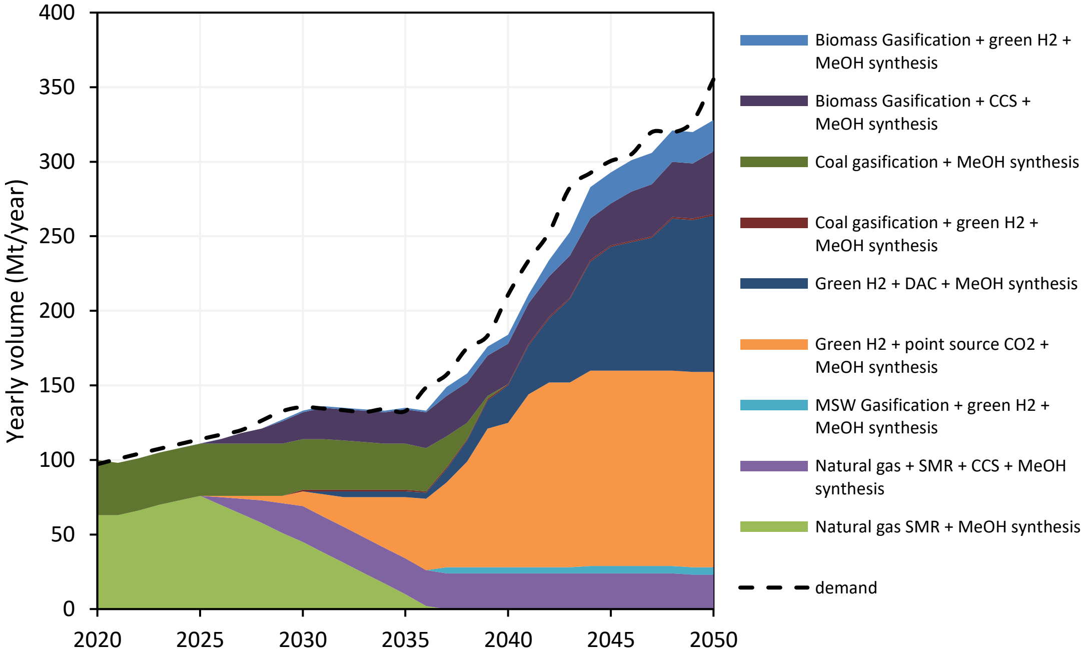

Fig. S84.
Technology over time for propylene demand and volumes under HC-NFAX scenario

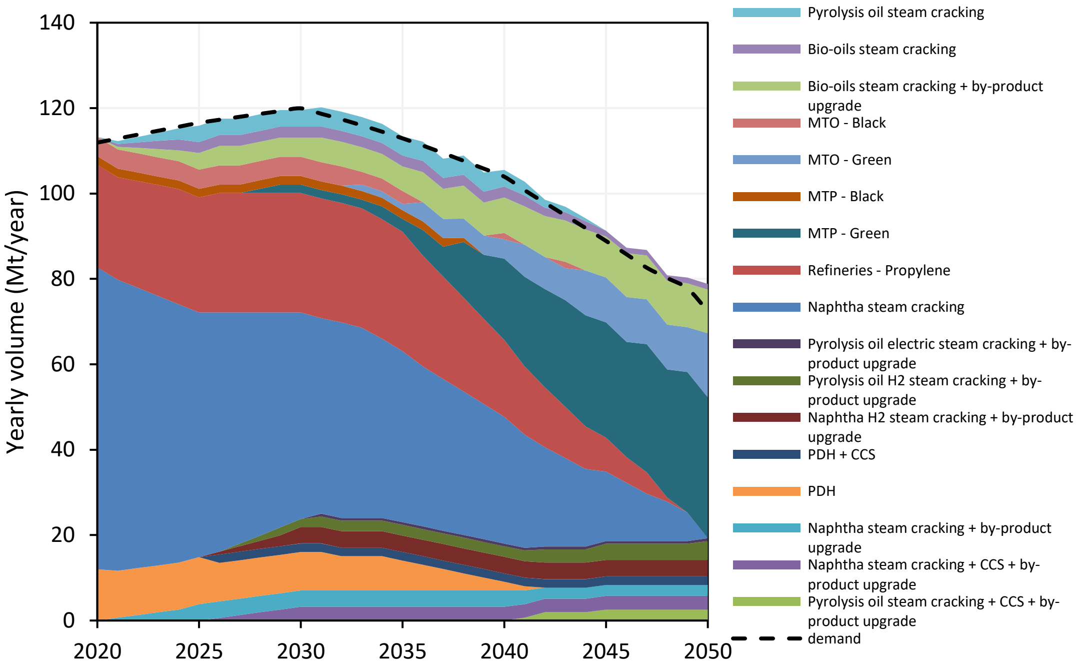

Fig. S85.
Technology over time for toluene demand and volumes under HC-NFAX scenario

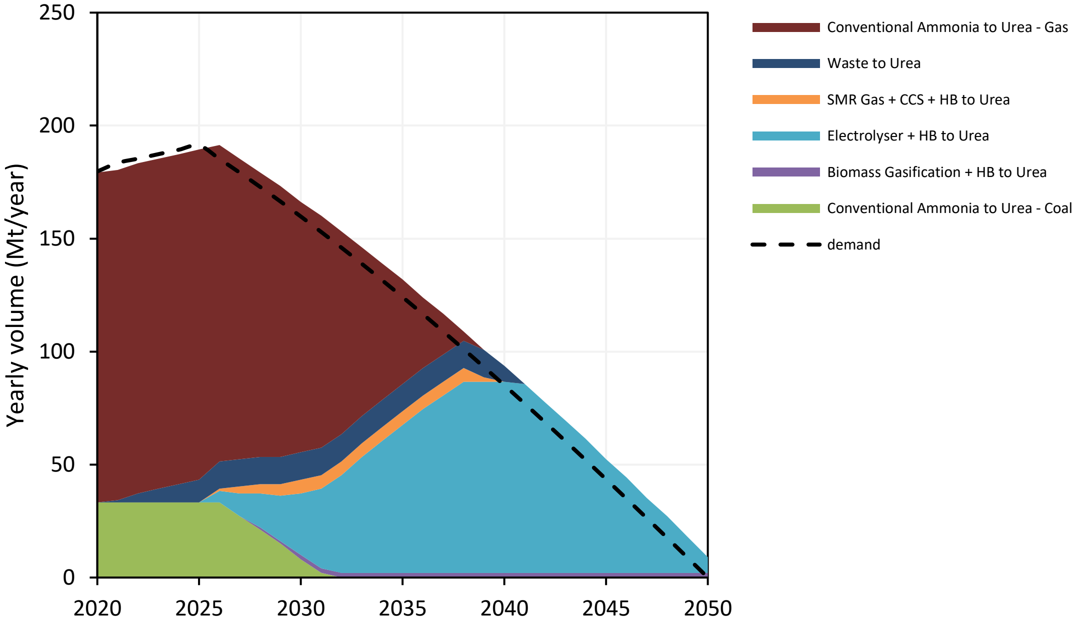

Fig. S86.
Technology over time for urea demand and volumes under HC-NFAX scenario

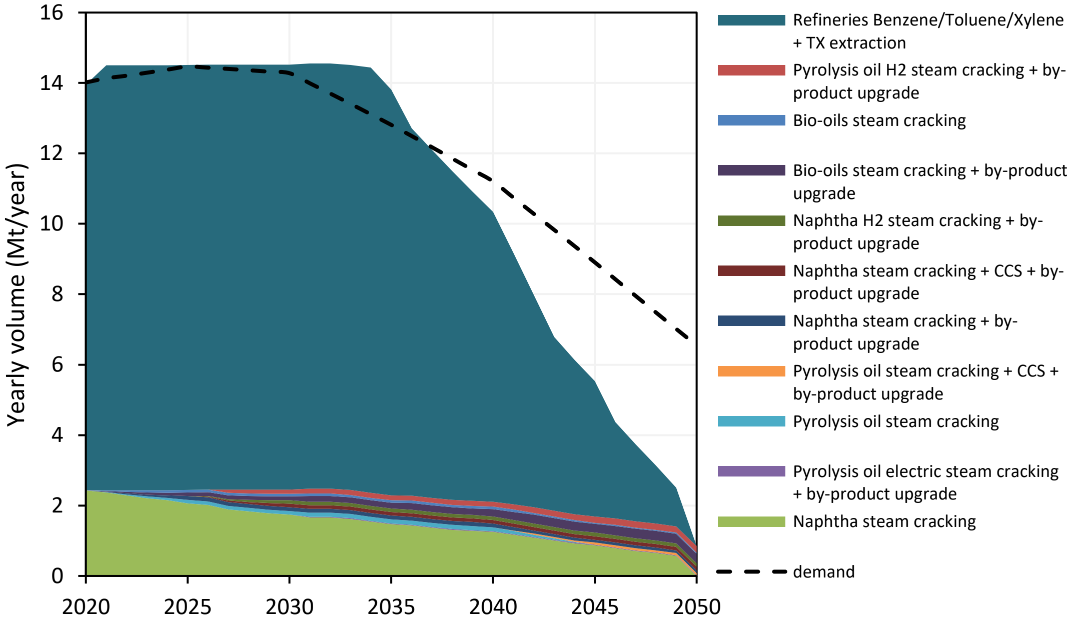

Fig. S87.
Technology over time for xylene demand and volumes under HC-NFAX scenario

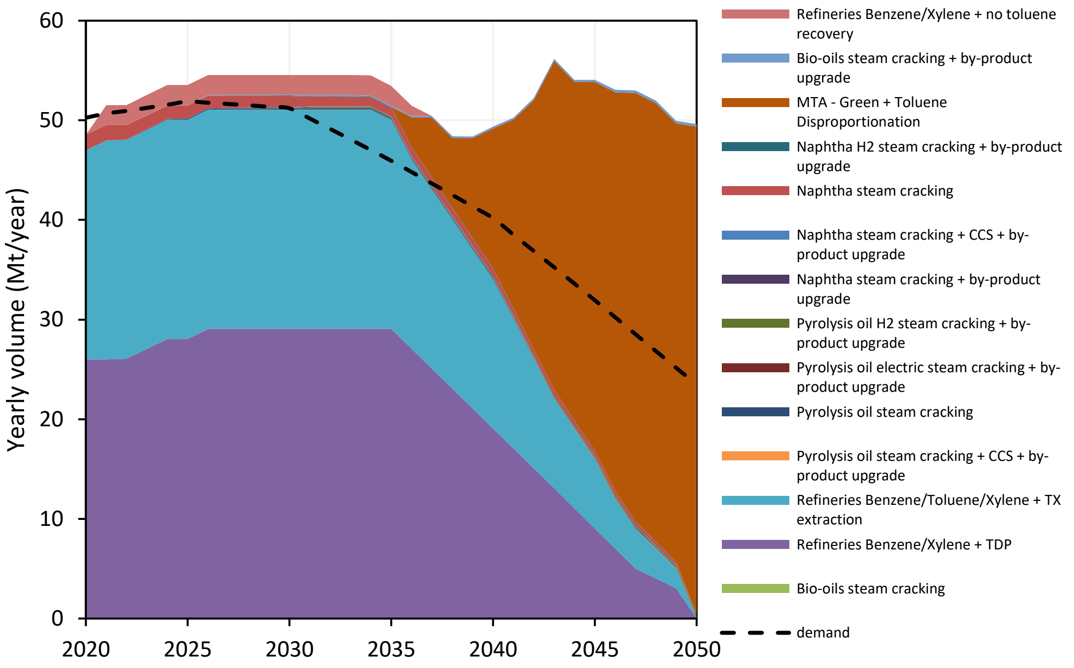

Fig. S88.
Technology over time for ammonia demand and volumes under HC-NFS scenario

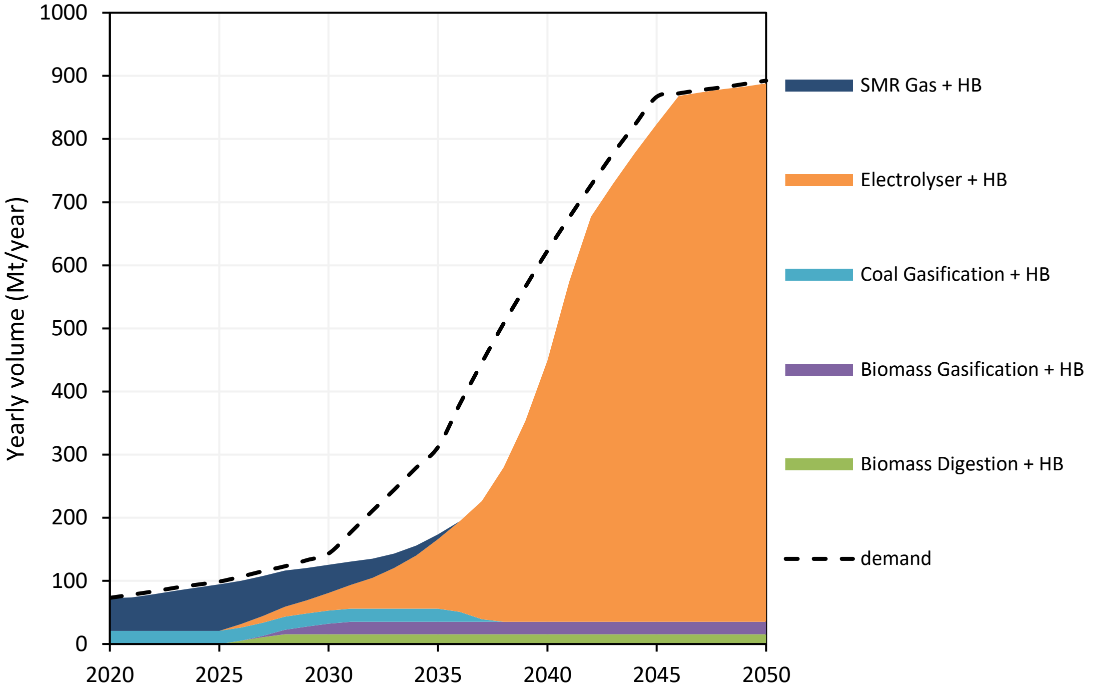

Fig. S89.
Technology over time for ammonium nitrate demand and volumes under HC-NFS scenario

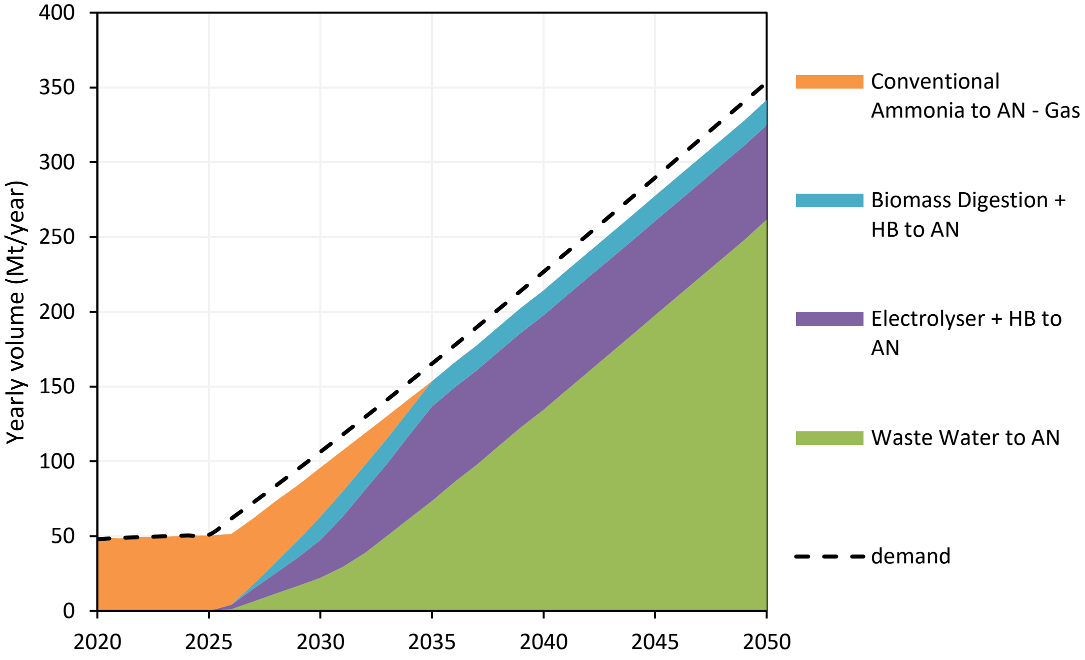

Fig. S90.
Technology over time for benzene demand and volumes under HC-NFS scenario

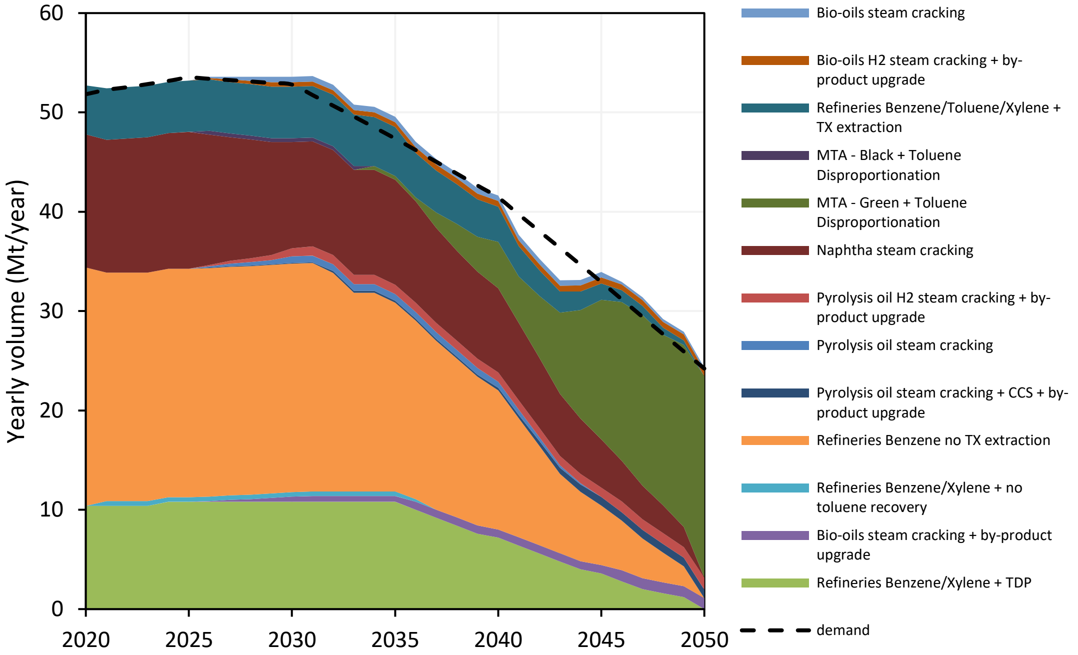

Fig. S91.
Technology over time for butadiene demand and volumes under HC-NFS scenario

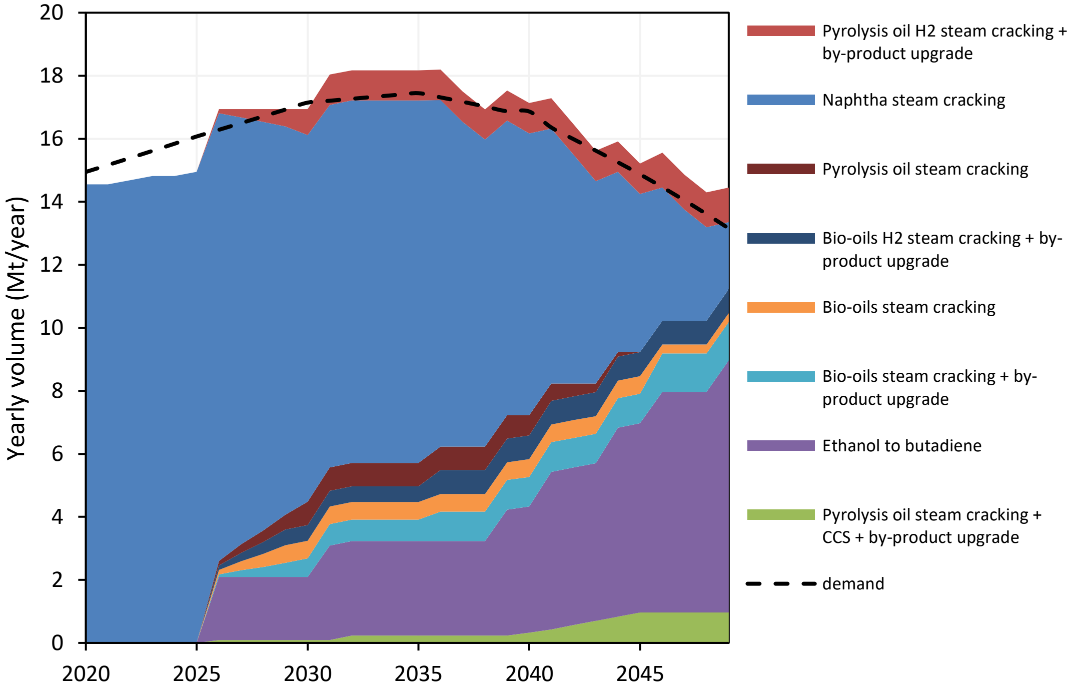

Fig. S92.
Technology over time for ethylene demand and volumes under HC-NFS scenario

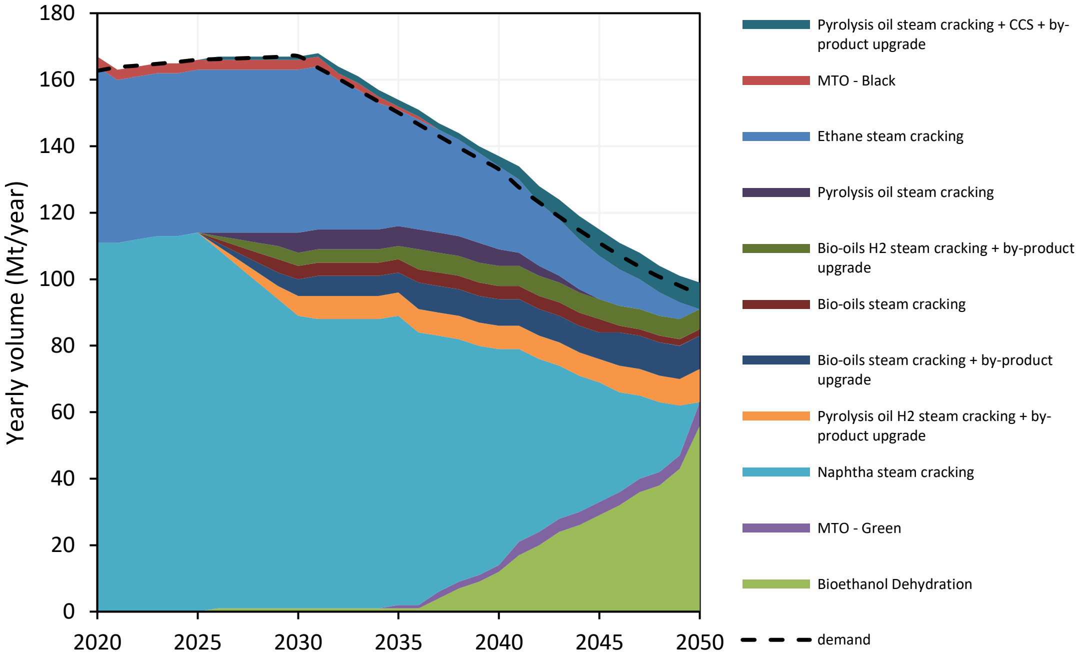

Fig. S93.
Technology over time for methanol demand and volumes under HC-NFS scenario

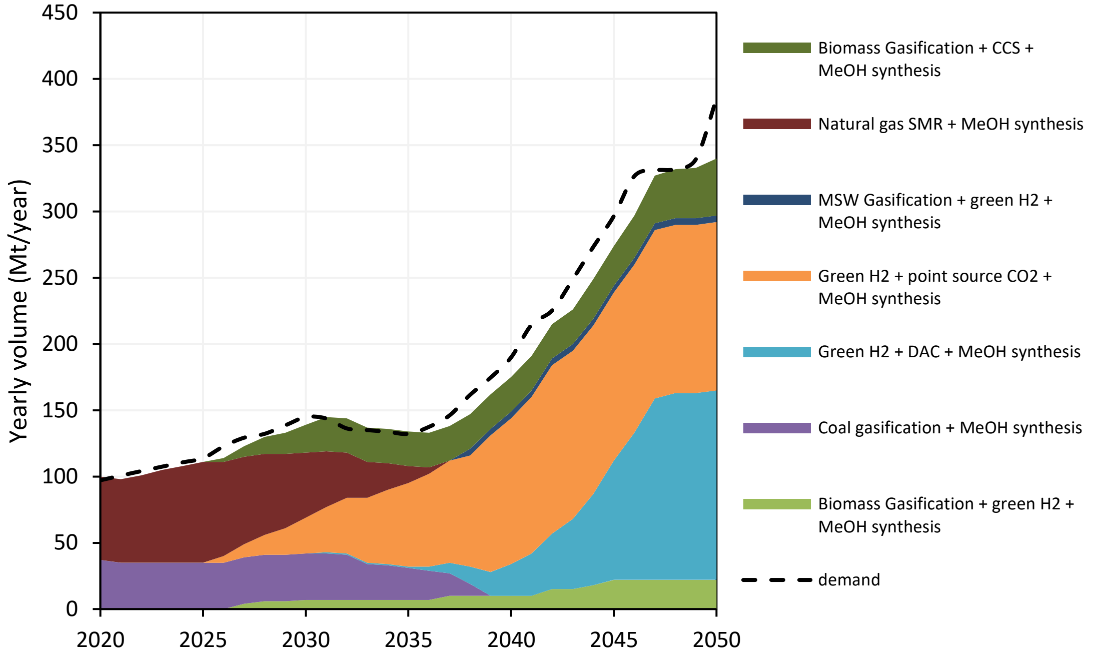

Fig. S94.
Technology over time for propylene demand and volumes under HC-NFS scenario

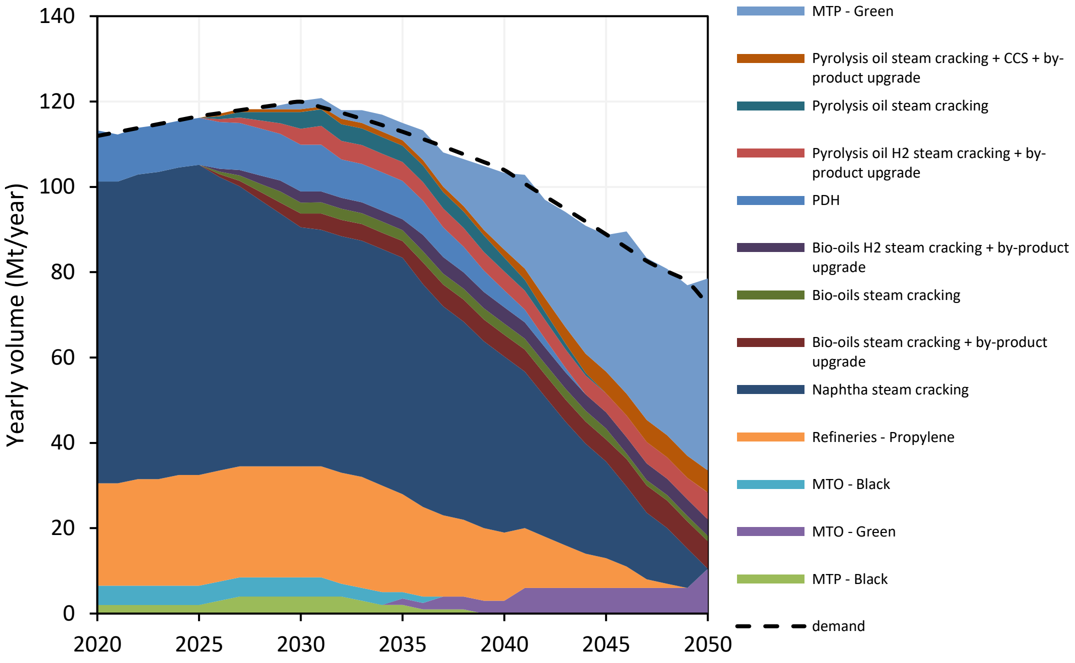

Fig. S95.
Technology over time for toluene demand and volumes under HC-NFS scenario

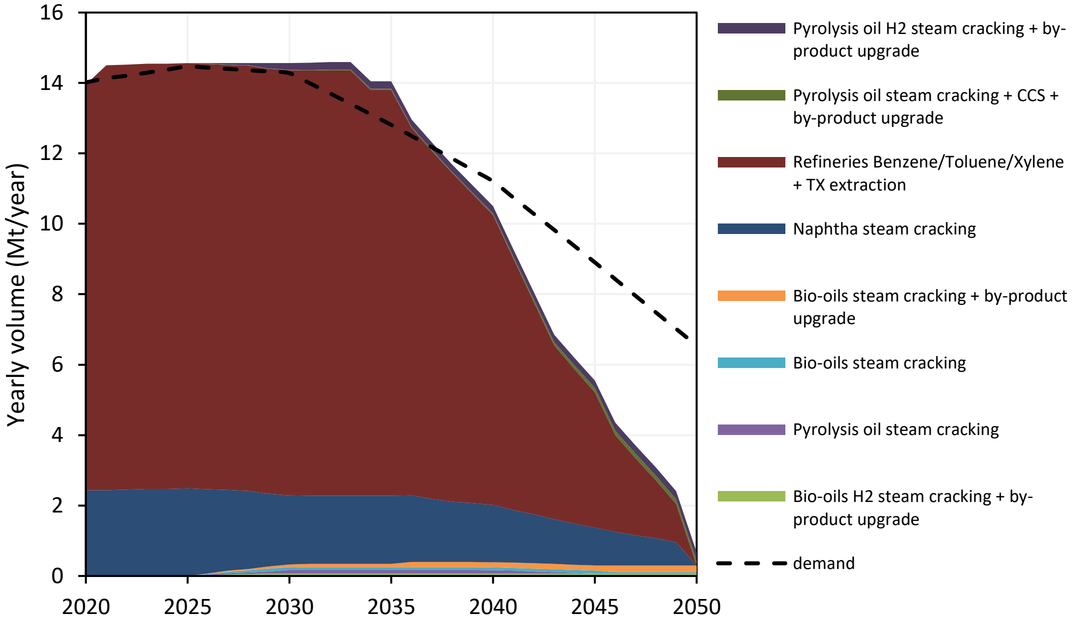

Fig. S96.
Technology over time for urea demand and volumes under HC-NFS scenario

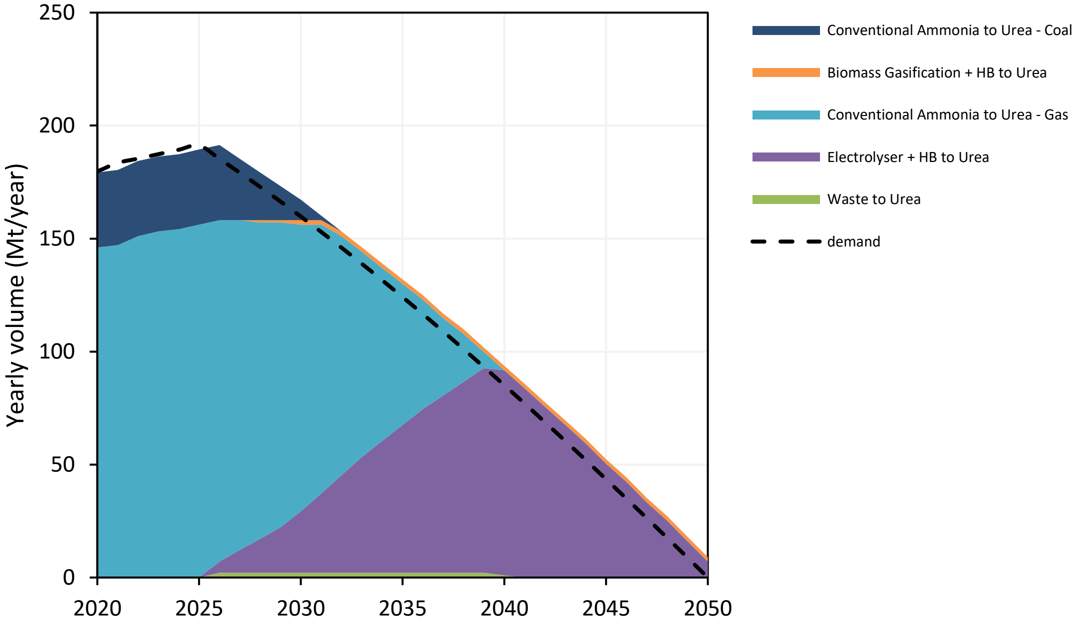

Fig. S97.
Technology over time for xylene demand and volumes under HC-NFS scenario

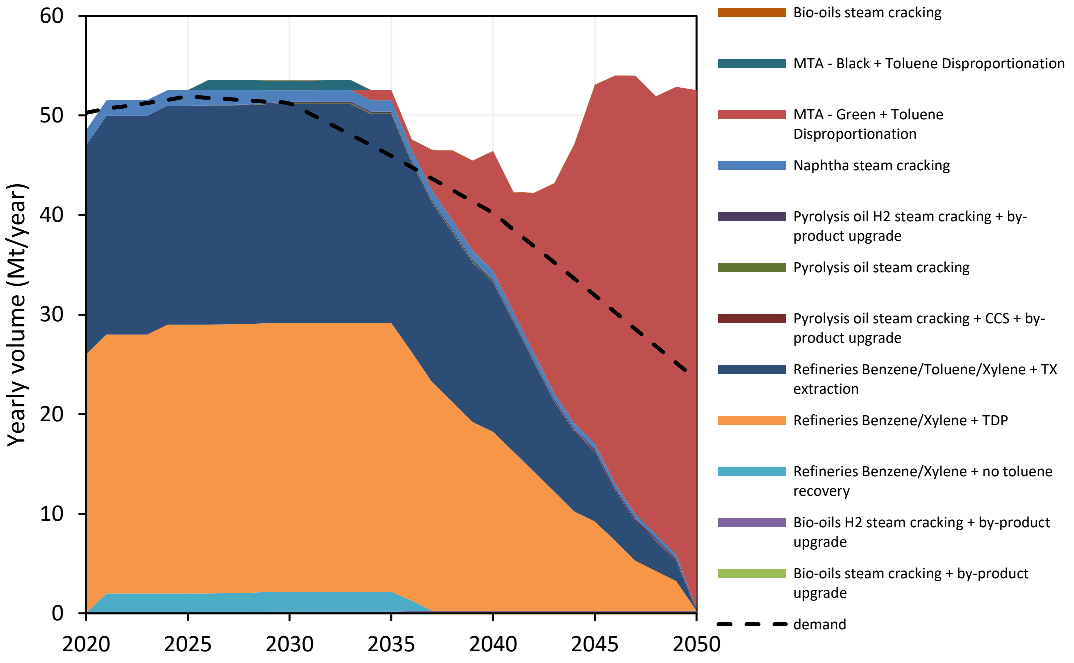

Supplementary Tables

Table S1.
Breakdown of open loop vs closed loop recycling across sectors

**GHG Protocol**		**This Study**	
**Scope & Category**	**Explanation**	**Explanation**	**In Scope (Y/N)**
Scope 1	Direct GHG emissions	Production of chemical products	Y
Scope 2	Electricity indirect GHG emissions		
Scope 3 Category 1	Purchased goods and services	Feedstock extraction	Y
Scope 3 Category 2	Capital goods	NA[9]	N
Scope 3 Category 3	Fuel and energy-related activities	NA	N
Scope 3 Category 4	Upstream transportation and distribution	NA	N
Scope 3 Category 5	Waste generated in operations	NA	N
Scope 3 Category 6	Business travel	NA	N
Scope 3 Category 7	Employee commuting	NA	N
Scope 3 Category 8	Upstream leased assets	NA	N
Scope 3 Category 9	Downstream transportation and distribution	NA	N
Scope 3 Category 10	Processing of sold products	NA	N
Scope 3 Category 11 (for fertilizers)	Use of sold products	Use-phase of chemical products	Y
Scope 3 Category 12	End-of-life treatment of sold products	End-of-life treatment of chemical products	Y
Scope 3 Category 13	Downstream leased assets	NA	N
Scope 3 Category 14	Franchises	NA	N
Scope 3 Category 15	Investments	NA	N

[9] NA=Not available

Table S2.
Breakdown of open loop vs closed loop recycling across sectors

		2020	2050
**Packaging and household goods**	% closed loop	33%	43%
	% open loop	67%	57%
**Transportation**	% closed loop	8%	80%
	% open loop	92%	20%
**Apparel**	% closed loop	5%	30%
	% open loop	95%	70%
**Buildings and Construction**	% closed loop	100%	100%
	% open loop	0%	0%
**Other sectors**	% closed loop	100%	100%
	% open loop	0%	0%

Table S3.

Breakdown of open loop recycling by industry in 2020

		TO					
		**Packaging and household goods**	**Transportation**	**Buildings and Construction**	**Apparel**	**Others and/or unspecified**	**Total**
**FROM**	**Packaging and household goods**	N/a	11%	63%	12%	14%	**100%**
	**Transportation**	12%	N/a	54%	0%	36%	**100%**
	**Buildings and Construction**	N/a	N/a	N/a	N/a	N/a	**N/a**
	**Apparel**	0%	0%	50%	N/a	50%	**100%**
	**Others**	N/a	N/a	N/a	N/a	N/a	**N/a**

Table S4.
Plastic waste management assumptions in 2020 and 2050

		**2020**	**BDEM-2050**	**LC&HC-2050**
**Collection rate**		84%	71%	96%
**Fate of remaining plastic waste post recycling**	**Incineration (no CCS)**	16%	22%	0%
	**Incineration (with CCS)**	0%	0%	32%
	**Landfill**	37%	15%	42%
	**Dumpsite**	8%	8%	1%
	**Open Burning**	27%	37%	16%
	**Environmental leakage**	7%	11%	6%
	**Ocean Leakage**	5%	7%	3%

Table S5.
Investment cost for waste management infrastructure

	**CAPEX (*$/ton of capacity*)**
**Mechanical Recycling**	1,172
**Chemical Recycling**	3,062
**Waste management (collection + MRF)**	1,341
**Incineration Unit (without CSS)**	548
**CCS unit for incineration retrofit**[1]	67
**Engineering landfill**	23

Note: (1) In tons of CO_2. Correspond to the investment cost of the CCS unit only, excluding the investment cost of incineration.

Table S6.
Emission factors for waste management treatments

	**2020** *tCO_2/t of plastic*	**2050 - BDEM** *tCO_2/t of plastic*	**2050 – LC/HC** *tCO_2/t of plastic*
**Mechanical Recycling**	0.48	0.48	0
**Chemical Recycling**	3.00	3.00	0
**Landfill**	0.01	0.01	0
**Dumpsite**	0.00	0.00	0
**Open Burning**	2.89	2.89	2.89
**Leakage Environment**	0	0	0
**Leakage Ocean**	0	0	0

Table S7.
Emission factors for waste management treatments

	**2020**<br>*tCO_2/t of chemical*	**2050 – BDEM (Incineration without CCS)**<br>*tCO_2/t of chemical*	**2050 – LC/HC (Incineration with CCS)**<br>*tCO_2/t of chemical*
**Ethylene**	1.35	2.8	0.07
**Propylene**	1.42	2.8	0.07
**Methanol**	1.38	2.1	0.07
**Benzene**	1.83	2.5	0.09
**Toluene**	1.39	2.5	0.07
**Xylene**	1.37	2.5	0.07
**Butadiene**	1.42	2.8	0.07

Table S8.
Emission factors for waste management treatments

	**2020** *tCO_2/t of NH_3*	**2050 - BDEM** *tCO_2/t of NH_3*	**2050 – LC/HC** *tCO_2/t of NH_3*
**Urea (as fertilizer)**	4.16	4.16	3.12
**Ammonium Nitrate (as fertilizer)**	1.80	1.80	1.35
**Other fertilizers**	2.98	2.98	2.24
**Power generation/Energy carrier**	0.01	0.01	0.01
**Others**	0	0	0

Table S9.
Supply Model Constraints

Constraining Variable	Description	Current Value	What it impacts
**Retrofit rate**	Assumption of the number of plants that could be converted in a year (either via retrofit or via decommissic + new build on-site), in the absence of plant age and investment cycle data	5% of chemical's previous year's capacity	The speed at which existing plants can undergo a technology switch
**Technology ramp up rate**	Speed at which technologies less than TRL 9 are implemented	Maximum of 4 plants per year or 30% increase of previous year's global capacity	Share of annual demand met by new technologies
**Forced decommission rate**	Speed at which remaining initial state technologies are phased out between 2035 and 2050 if no other aforementioned drivers in the model can achieve this	Increasing rate, see "Decommission" in "Master template.xlsx"	Share of 2050 demand met by new technologies
**Geographic cap**	Assumption to take into account geopolitics of chemical industry market	30% of all chemicals in one region	The maximum share of global demand that one geographic region can meet
**Available feedstock and energy sources**	Assumption that particular resources are finite in their availability (e.g., biomass)	See Section 5.7	Share of demand that can be met by technologies that require finite feedstock/energy sources
**Available CO_2 storage**	Assumption that build out of ready-to-use CO_2 transport and storage infrastructure will take time to scale and therefore must be capped	See Section 5.7	Share of demand that can be met by technologies that have CCS built in

Table S10.
Key equations for calculations in supply model. Emissions scope are translated into lifecycle emissions stages according to Table S1.

	Output	Output unit	Equation
1	Decommissioning cost	USD/t product/ annum	5% * (CAPEX of new build plant)
2	Cost CCS Transport and Storage (T&S)	USD/t product/ annum	(Price, CCS Transport &Storage) * (Captured carbon (calculated below))
3	Variable OPEX	USD/t product/ annum	Cost CCS T&S (calculated above) + $\sum^{\text{All feedstock inputs}}$ {Amount of feedstock x} ∗ {Price of feedstock x} + $\sum^{\text{All energy inputs}}$ {Amount of energy source x} ∗ {Price of energy source x}]
	Scope 1 emissions	t CO_2/t product	($\sum^{\text{All feedstock inputs}}$ {Amount of feedstock x} ∗ {Scope 1 emission factor of feedstock x} + $\sum^{\text{All energy inputs}}$ {Amount of energy source x} ∗ {Scope 1 emission factor of energy source x}) * (1 – {CCS Capture rate})
4	Scope 2 emissions	t CO_2/t product	(Amount of grid power) * (Scope 2 emission factor of grid power)
	Scope 3 upstream emissions	t CO_2/t product	$\sum^{\text{All feedstock inputs}}$ {Amount of feedstock x} ∗ {Scope 3 upstream emission factor of feedstock x} + $\sum^{\text{All energy inputs}}$ {Amount of energy sourcex} ∗ {Scope 3 upstream emission factor of energy source x})
	Captured carbon	t CO_2/t product	($\sum^{\text{All feedstock inputs}}$ {Amount of feedstock x} ∗ {Scope 1 emission factor of feedstock x} + $\sum^{\text{All energy inputs}}$ {Amount of energy source x} ∗ {Scope 1 emission factor of energy source x}) * (CCS Capture rate)
5	Total emissions	t CO_2/t product	Scope 1 emissions (calculated above) + Scope 2 emissions (calculated above) + Scope 3 upstream emissions (calculated above)
6	Total Cost of Ownership (TCO)	USD	[(Annualised CAPEX) * (Daily plant capacity) * 365 * (Plant capacity factor %)] + ∑ Discounted {[(O&M) + (Variable OPEX)] * [(Daily plant capacity) * 365 * (Plant capacity factor %)]}
7	Levelized cost of chemical X (LCOX)	USD/t product	TCO/ Discounted [(Daily plant capacity) * 365 * (Plant capacity factor %)]
8	CO2 abated	t CO_2/t product	Total emissions of business case **X** – Total emissions of this business case

Table S11.
CCS Transport and Storage cost

Region	Tiers according to Smith et al. (48)	CCS Transport and Storage cost (USD/t CO_2)
North America	Tier 1 – low cost	11.22
Latin America	Tier 2 – medium cost	15.92
Africa	Tier 3 – high cost	25.86
Middle East	Tier 1 – low cost	11.22
Southeast Asia	Tier 2 – medium cost	15.92
South Asia	Average of Tier 2 – medium cost and Tier 3 – high cost	20.89
Oceania	Tier 2 – medium cost	15.92
China	Tier 2 – medium cost	15.92
Russia	Tier 1 – low cost	11.22
Europe	Tier 4 – shipping cost	35.80

Table S12.
Production volume and share of old plants in 2020 by chemical, technology, and region

Region	Technology	Chemical	Year	Production volume (Mt/year)	Percentage of "old" plants
North America	SMR Gas + HB	Ammonia	2020	5.7	100%
Latin America	SMR Gas + HB	Ammonia	2020	0.0	100%
Africa	SMR Gas + HB	Ammonia	2020	2.8	100%
Middle East	SMR Gas + HB	Ammonia	2020	11.7	100%
Russia	SMR Gas + HB	Ammonia	2020	14.5	100%
Europe	SMR Gas + HB	Ammonia	2020	9.8	100%
China	SMR Gas + HB	Ammonia	2020	5.7	100%
China	Coal Gasification + HB	Ammonia	2020	21.3	100%
India	SMR Gas + HB	Ammonia	2020	0.6	100%
Japan	SMR Gas + HB	Ammonia	2020	1.0	100%
Rest of Asia and Pacific	SMR Gas + HB	Ammonia	2020	0.3	100%
North America	Conventional Ammonia to Urea - Gas	Urea	2020	27.0	100%
Latin America	Conventional Ammonia to Urea - Gas	Urea	2020	12.0	100%
Africa	Conventional Ammonia to Urea - Gas	Urea	2020	10.0	100%
Middle East	Conventional Ammonia to Urea - Gas	Urea	2020	9.6	100%
Russia	Conventional Ammonia to Urea - Gas	Urea	2020	2.9	100%
Europe	Conventional Ammonia to Urea - Gas	Urea	2020	16.0	100%
China	Conventional Ammonia to Urea - Gas	Urea	2020	5.6	100%
China	Conventional Ammonia to Urea - Coal	Urea	2020	33.7	100%
India	Conventional Ammonia to Urea - Gas	Urea	2020	25.8	100%
Japan	Conventional Ammonia to Urea - Gas	Urea	2020	0.0	100%
Rest of Asia and Pacific	Conventional Ammonia to Urea - Gas	Urea	2020	35.8	100%
North America	Conventional Ammonia to AN - Gas	Ammonium Nitrate	2020	1.9	100%
Latin America	Conventional Ammonia to AN - Gas	Ammonium Nitrate	2020	1.9	100%
Africa	Conventional Ammonia to AN - Gas	Ammonium Nitrate	2020	5.6	100%
Middle East	Conventional Ammonia to AN - Gas	Ammonium Nitrate	2020	1.7	100%
Russia	Conventional Ammonia to AN - Gas	Ammonium Nitrate	2020	10.8	100%

Europe	Conventional Ammonia to AN - Gas	Ammonium Nitrate	2020	21.7	100%
China	Conventional Ammonia to AN - Gas	Ammonium Nitrate	2020	0.0	100%
China	Conventional Ammonia to AN - Coal	Ammonium Nitrate	2020	0.0	100%
India	Conventional Ammonia to AN - Gas	Ammonium Nitrate	2020	0.0	100%
Japan	Conventional Ammonia to AN - Gas	Ammonium Nitrate	2020	0.0	100%
Rest of Asia and Pacific	Conventional Ammonia to AN - Gas	Ammonium Nitrate	2020	5.7	100%
North America	Ethane steam cracking	Ethylene	2020	36.6	38%
North America	Naphtha steam cracking	Ethylene	2020	2.1	73%
Latin America	Ethane steam cracking	Ethylene	2020	2.6	100%
Latin America	Naphtha steam cracking	Ethylene	2020	3.7	100%
Europe	Ethane steam cracking	Ethylene	2020	2.2	100%
Europe	Naphtha steam cracking	Ethylene	2020	18.3	67%
Russia	Naphtha steam cracking	Ethylene	2020	4.9	100%
Africa	Naphtha steam cracking	Ethylene	2020	1.6	100%
Middle East	Ethane steam cracking	Ethylene	2020	9.5	0%
Middle East	Naphtha steam cracking	Ethylene	2020	19.2	44%
China	Naphtha steam cracking	Ethylene	2020	26.7	37%
India	Naphtha steam cracking	Ethylene	2020	4.3	71%
India	Ethane steam cracking	Ethylene	2020	1.1	0%
Japan	Naphtha steam cracking	Ethylene	2020	6.4	0%
Rest of Asia and Pacific	Naphtha steam cracking	Ethylene	2020	24.1	52%
China	MTO - Black	Ethylene	2020	3.5	0%
North America	Refineries - Propylene	Propylene	2020	7.5	93%
North America	PDH	Propylene	2020	3.4	56%
Europe	Refineries - Propylene	Propylene	2020	3.9	80%
Russia	PDH	Propylene	2020	0.5	100%
Middle East	Refineries - Propylene	Propylene	2020	2.2	100%
China	Refineries - Propylene	Propylene	2020	6.0	100%
China	PDH	Propylene	2020	6.8	0%
India	Refineries - Propylene	Propylene	2020	0.6	100%
Japan	Refineries - Propylene	Propylene	2020	1.9	100%
Rest of Asia and Pacific	Refineries - Propylene	Propylene	2020	2.3	73%
Rest of Asia and Pacific	PDH	Propylene	2020	1.7	100%
China	MTP - Black	Propylene	2020	2.5	32%
North America	Refineries Benzene/Xylene + TDP	Benzene	2020	1.0	100%

Latin America	Refineries Benzene/Xylene + TDP	Benzene	2020	0.0	100%
Africa	Refineries Benzene/Xylene + TDP	Benzene	2020	0.1	100%
Middle East	Refineries Benzene/Xylene + TDP	Benzene	2020	1.5	100%
Russia	Refineries Benzene/Xylene + TDP	Benzene	2020	0.1	100%
Europe	Refineries Benzene/Xylene + TDP	Benzene	2020	0.4	100%
China	Refineries Benzene/Xylene + TDP	Benzene	2020	3.1	100%
India	Refineries Benzene/Xylene + TDP	Benzene	2020	0.1	100%
Japan	Refineries Benzene/Xylene + TDP	Benzene	2020	2.1	100%
Rest of Asia and Pacific	Refineries Benzene/Xylene + TDP	Benzene	2020	2.5	100%
North America	Refineries Benzene no TX extraction	Benzene	2020	3.9	100%
Latin America	Refineries Benzene no TX extraction	Benzene	2020	0.4	100%
Africa	Refineries Benzene no TX extraction	Benzene	2020	0.0	100%
Middle East	Refineries Benzene no TX extraction	Benzene	2020	0.6	100%
Russia	Refineries Benzene no TX extraction	Benzene	2020	0.7	100%
Europe	Refineries Benzene no TX extraction	Benzene	2020	3.7	100%
China	Refineries Benzene no TX extraction	Benzene	2020	5.6	100%
India	Refineries Benzene no TX extraction	Benzene	2020	1.5	100%
Japan	Refineries Benzene no TX extraction	Benzene	2020	0.7	100%
Rest of Asia and Pacific	Refineries Benzene no TX extraction	Benzene	2020	5.2	100%
North America	Refineries Benzene/Toluene/Xylene + TX extraction	Toluene	2020	2.0	100%
Latin America	Refineries Benzene/Toluene/Xylene + TX extraction	Toluene	2020	0.6	100%
Africa	Refineries Benzene/Toluene/Xylene + TX extraction	Toluene	2020	0.0	100%
Middle East	Refineries Benzene/Toluene/Xylene + TX extraction	Toluene	2020	1.2	100%
Russia	Refineries Benzene/Toluene/Xylene + TX extraction	Toluene	2020	0.1	100%

Europe	Refineries Benzene/Toluene/Xylene + TX extraction	Toluene	2020	1.0	100%
China	Refineries Benzene/Toluene/Xylene + TX extraction	Toluene	2020	4.3	100%
India	Refineries Benzene/Toluene/Xylene + TX extraction	Toluene	2020	0.0	100%
Japan	Refineries Benzene/Toluene/Xylene + TX extraction	Toluene	2020	0.7	100%
Rest of Asia and Pacific	Refineries Benzene/Toluene/Xylene + TX extraction	Toluene	2020	1.7	100%
North America	Refineries Benzene/Xylene + no toluene recovery	Xylene	2020	0.0	100%
Latin America	Refineries Benzene/Xylene + no toluene recovery	Xylene	2020	0.0	100%
Africa	Refineries Benzene/Xylene + no toluene recovery	Xylene	2020	0.0	100%
Middle East	Refineries Benzene/Xylene + no toluene recovery	Xylene	2020	0.0	100%
Russia	Refineries Benzene/Xylene + no toluene recovery	Xylene	2020	0.0	100%
Europe	Refineries Benzene/Xylene + no toluene recovery	Xylene	2020	0.0	100%
China	Refineries Benzene/Xylene + no toluene recovery	Xylene	2020	0.0	100%
India	Refineries Benzene/Xylene + no toluene recovery	Xylene	2020	0.0	100%
Japan	Refineries Benzene/Xylene + no toluene recovery	Xylene	2020	0.0	100%
Rest of Asia and Pacific	Refineries Benzene/Xylene + no toluene recovery	Xylene	2020	0.0	100%
China	Coal gasification + MeOH synthesis	Methanol	2020	36.8	100%
North America	Natural gas SMR + MeOH synthesis	Methanol	2020	9.6	100%
Latin America	Natural gas SMR + MeOH synthesis	Methanol	2020	9.9	100%
Africa	Natural gas SMR + MeOH synthesis	Methanol	2020	2.3	100%
Middle East	Natural gas SMR + MeOH synthesis	Methanol	2020	20.4	100%

China	Natural gas SMR + MeOH synthesis	Methanol	2020	9.2	100%
Rest of Asia and Pacific	Natural gas SMR + MeOH synthesis	Methanol	2020	4.1	100%
Russia	Natural gas SMR + MeOH synthesis	Methanol	2020	5.5	100%
Japan	Natural gas SMR + MeOH synthesis	Methanol	2020	1.5	100%
India	Natural gas SMR + MeOH synthesis	Methanol	2020	0.2	100%
Europe	Natural gas SMR + MeOH synthesis	Methanol	2020	2.7	100%

References

1. L. Persson, *et al.*, Outside the Safe Operating Space of the Planetary Boundary for Novel Entities. *Environ. Sci. Technol.* **56**, 1510–1521 (2022).

2. ChemSec – the International Chemical Secretariat, "What goes around - Enabling the circular economy by removing chemical roadblocks" (2021).

3. International Energy Agency, "The Future of Petrochemicals - Towards more sustainable plastics and fertilisers" (2018).

4. D. Saygin, D. Gielen, Zero-Emission Pathway for the Global Chemical and Petrochemical Sector. *Energies*, 28 (2021).

5. R. Meys, *et al.*, Achieving net-zero greenhouse gas emission plastics by a circular carbon economy. *Science* **374**, 71–76 (2021).

6. DECHEMA & FutureCamp, "Roadmap Chemie 2050: Auf dem Weg zu einer treibhausgasneutralen chemiscshen Industrie in Deutschland" (2019).

7. DECHEMA, "Low carbon energy and feedstock for the European chemical industry" (2017).

8. International Energy Agency, International Council of Chemical Associations, Dechema Gesellschaft für chemische Technik und Biotechnologie e.V., Technology Roadmap - Energy and GHG Reductions in the Chemical Industry via Catalytic Processes (2013).

9. , *Greenhouse gas protocol: corporate value chain (Scope 3) accounting and reporting standard: supplement to the GHG protocol corporate accounting and reporting standard.* (World Resources Institute; World Business Council for Sustainable Development, 2011).

10. Energy Transition Commission, "Bioresources within a Net-Zero Emissions Economy: Making a Sustainable Approach Possible" (2021).

11. The Food and Land Use Coalition, "Growing Better: Ten Critical Transitions to Transform Food and Land Use" (2019) (April 25, 2022).

12. International Energy Agency, *Ammonia Technology Roadmap: Towards more sustainable nitrogen fertiliser production* (OECD, 2021) https:/doi.org/10.1787/f6daa4a0-en (October 20, 2021).

13. SYSTEMIQ & The Pew Charitable Trust, "Breaking the Plastic Wave: A comprehensive assessment of pathways towards stopping ocean plastic pollution" (2020).

14. P. G. Levi, J. M. Cullen, Mapping Global Flows of Chemicals: From Fossil Fuel Feedstocks to Chemical Products. *Environ. Sci. Technol.* **52**, 1725–1734 (2018).

15. R. Geyer, J. R. Jambeck, K. L. Law, Production, use, and fate of all plastics ever made. *Sci. Adv.* **3**, e1700782 (2017).

16. Saskia Manshoven, Anse Smeets, Mona Arnold, Lars Fogh Mortensen (last), Plastic in textiles: potentials for circularity and reduced environmental and climate impacts (2021).

17. Dr Tristan Smith, Dr Aly Shaw, Jesse Fahnestock, The Role of the Energy Sector in Shipping's Fuel Transition (2021).

18. , Japan's Top Power Producer Seeks Long-Term Clean Ammonia Deal. *Bloomberg* (2022).

19. Korean Ministry of Trade Industry and Energy, "Generate electricity from carbon-free fuels hydrogen and ammonia" (2022).

20. Energy Transition Commission, "Making Clean Electrification Possible: 30 Years to Electrify the Global Economy" (2021) (April 25, 2022).

21. European Commission. Joint Research Centre., "Raw materials demand for wind and solar PV technologies in the transition towards a decarbonised energy system." (Publications Office, 2020) (April 21, 2022).

22. Fertilisers Europe, Carbon Footprint Reference Values - Energy efficiency and greenhouse gas emissions in European mineral fertiliser production and use.

23. Plastics Europe, "Plastics - the Facts 2020" (2020).

24. SYSTEMIQ, "XaaS - Everything-as-a-Service: How businesses can thrive in the age of climate change." (2021).

25. Renilde Becqué, Samuel Sharp, "Phasing out plastics: The automotive sector" (ODI, 2020) (April 25, 2022).

26. S. Pickard, S. Sharp, "Phasing out plastics: The construction sector" (ODI, 2020) (April 25, 2022).

27. B. Oberle, *et al.*, Global resources outlook: 2019 (2019).

28. McKinsey & Company, Fashion on climate: How the fashion industry can be urgently act to reduce its greenhouse gas emissions (2020) (April 25, 2022).

29. OECD, *Improving Markets for Recycled Plastics: Trends, Prospects and Policy Responses* (OECD, 2018) https:/doi.org/10.1787/9789264301016-en (April 22, 2022).

30. The Ellen MacArthur Foundation, "A New Textiles Economy: Redesigning fashion's future" (2017).

31. SYSTEMIQ, ReShaping Plastics: Pathways to a Circular, Climate Neutral Plastics System in Europe (2022) (April 25, 2022).

32. Waste & Resources Action Programme (WRAP), "Plastics Market Situation Report" (2019).

33. Closed Loop Partners, "Accelerating circular supply chains for plastics: a landscape of transformational technologies that stop plastic waste, keep materials in play and grow markets" (2019).

34. Energy Transition Commission, "Making the green hydrogen economy possible: accelerating green hydrogen in an electrified economy" (2021).

35. U.S. Environmental Protection Agency, "Documentation for Greenhouse Gas Emission and Energy Factors Used in the Waste Reduction Model (WARM)" (2019).

36. Intergovernmental Panel on Climate Change (IPCC), "Climate Change 2022 - Mitigation of Climate Change (Working Group III contribution to the Sixth Assessment Report of the Intergovernmental Panel on Climate Change)" (2022) (April 21, 2022).

37. R. J. Lempert, D. G. Groves, S. W. Popper, S. C. Bankes, A General, Analytic Method for Generating Robust Strategies and Narrative Scenarios. *Management Science* **52**, 514–528 (2006).

38. W. E. Walker, M. Haasnoot, J. H. Kwakkel, Adapt or Perish: A Review of Planning Approaches for Adaptation under Deep Uncertainty. *Sustainability* **5**, 955–979 (2013).

39. Energy Transitions Commission (ETC), "Making the Hydrogen Economy Possible: Accelerating Clean Hydrogen in an Electrified Economy" (2021) (November 24, 2021).

40. Intratec, "Benzene Production from Naphtha - Benzene E51A Advanced Cost Analysis" (2021).

41. Intratec, "Butadiene Production from Ethanol - Butadiene E31A Extended Cost Analysis" (2021).

42. K. Girod, H. Lohmann, S. Schlüter, S. Kaluza, Methanol Synthesis with Steel-Mill Gases: Simulation and Practical Testing of Selected Gas Utilization Scenarios. *Processes* **8**, 1673 (2020).

43. S. Khalafalla, *et al.*, Conceptual Design Development of Coal-to-Methanol Process with Carbon Capture and Utilization. *Energies* **13**, 6421 (2020).

44. M. Worley, J. Yale, "Biomass Gasification Technology Assessment: Consolidated Report" (2012).

45. IEAGHG, "Techno-Economic Evaluation of HYCO Plant Integrated to Ammonia/Urea or Methanol Production with CCS" (2017).

46. IEAGHG, "Towards Zero Emissions CCS in Power Plants Using Higher Capture Rates or Biomass" (2019).

47. International Energy Agency, "Net Zero by 2050" (2021).

48. E. Smith, *et al.*, The Cost of CO2 Transport and Storage in Global Integrated Assessment Modeling in (2021).

49. Global CCS Institute, Global Status of CCS 2021 - CCS Accelerating to Net Zero (2021).

50. , Handbook on Supply and Use Tables and Input-Output Tables with Extensions and Applications. 735.

51. K. Miyazawa, *Sangyo renkan bunseki nyumon.* (2002).

52. Bureau of Economic Analysis, US Leontief inverse of input-output matrix (2012).

53. e-stat, Japan's Leontief inverse of input-output matrix (2015) (May 27, 2022).
